# Barriers to and facilitators of healthcare access among people experiencing homelessness: a systematic scoping review

**DOI:** 10.1186/s12939-026-02840-z

**Published:** 2026-04-03

**Authors:** Daniela Gorski, Filipa Alves da Costa, Ricardo Fuertes, João Gama-Marques, Lara Tavoschi, Fernanda S. Tonin

**Affiliations:** 1 Pharmaceutical Sciences Postgraduate Program, Health Sciences Sector, Federal University of Paraná, Paraná, Curitiba Brazil; 2 Public Health and Medicines Use Research Lab, Research Institute for Medicines (iMED.ULisboa), University of Lisbon, Faculty of Pharmacy, Lisbon, Portugal; 3 Department of Translational Research and New Technologies in Medicine and Surgery, University of Pisa, Pisa, Italy; 4 Homeless Outreach Psychiatric Engagement for Lisboa (HOPE 4 Lisboa) & Consulta de Esquizofrenia Resistente (CER), Hospital Júlio de Matos (HJM), Unidade Local de Saúde de São José (ULSSJ), Centro Clínico Académico de Lisboa (CCAL), Lisboa, PT Portugal; 5 Pharmacy and Pharmaceutical Technology Department, Social and Legal Pharmacy Section, University of Granada, Granada, Spain

**Keywords:** Homeless, Unhoused, Ill-housed persons, Patient care, Health services accessibility, Social stigma

## Abstract

**Background:**

People experiencing homelessness (PEH) face complex social and economic challenges that increase their risk of poor health. This study aimed to identify and synthesize key barriers to and facilitators of healthcare access from the perspective of PEH to inform more equitable and sustainable health strategies.

**Methods:**

A systematic scoping review was conducted in accordance with the guidelines of the Cochrane Collaboration and the Joanna Briggs Institute and was reported following the Preferred Reporting Items for Scoping Reviews (PRISMA-ScR). A PubMed, Embase and Web of Science search was conducted in January 2025 using terms related to homelessness, healthcare, and interventional and observational studies. A manual search of the reference lists of the included studies was also performed via conventional search engines. Two reviewers independently classified the relevance of the extracted studies according to predefined eligibility criteria. Any discrepancies were solved by a third reviewer. The final list of studies enabled extraction of barriers to and facilitators of access to healthcare, subsequently classified according to the socioecological model.

**Results:**

A total of 79 studies (*n* = 51,110 PEH) published between 1989 and 2024, mostly from the United States of America (USA) (*n* = 48; 63.3%), were included. Interventions most frequently addressed general healthcare (*n* = 25; 31.6%), treatment of specific conditions (*n* = 23; 29.1%) and sexual and reproductive healthcare (*n* = 7; 8.9%). Common individual-level barriers included health related-beliefs and concerns (*n* = 43), cognitive and behavioral health challenges (*n* = 28), and substance use (*n* = 18), whereas increased health awareness (*n* = 13) facilitated healthcare utilization. At the interpersonal level, social stigma (*n* = 51) and negative provider attitudes (*n* = 31) were prominent barriers, whereas strong social networks (*n* = 34) supported engagement. Institutional-level barriers included bureaucracy (*n* = 21) and lack of service integration (*n* = 20); conversely, continuity of care (*n* = 8) and process simplification (*n* = 7) acted as facilitators. Community-level barriers involved limited-service availability (*n* = 22), direct (*n* = 34) and indirect costs (*n* = 19); facilitators were centered on structure of the health service (*n* = 16). At the policy level, limited resources and service offer (*n* = 7), as well as undocumented situations by PEH (*n* = 4) were key barriers; strategically located services (*n* = 7) facilitated healthcare utilization.

**Conclusion:**

Improving healthcare for PEH requires multilevel, person-centered strategies that address structural, interpersonal, and individual barriers while streamlining access through inclusive public policy.

**Review registration:**

International Prospective Register of Systematic Reviews (PROSPERO): CRD42025635835.

**Supplementary Information:**

The online version contains supplementary material available at 10.1186/s12939-026-02840-z.

## Background

Homelessness is a pressing global issue at the intersection of social justice, public health, and human rights [1–3]. This includes people who are roofless, meaning they live without a shelter of any kind (such as in public places, including parks, streets, bus/metro stops, viaducts, etc.), or with a place to sleep but temporarily in institutions or shelters (including emergency ones). It also includes houseless people, meaning they do not have a house and are hosted by social programs, which may include temporary accommodation centers while they wait for referral to the most appropriate program, and those who live in insecure housing, with the possibility of being threatened with severe exclusion due to insecure tenancies, eviction or domestic violence. Moreover, it may also include people living in inadequate housing, such as caravans on illegal campsites, abandoned cars, in unfit or precarious housing, or in extreme overcrowding [4–8]. Between 2023 and 2024, the number of people without adequate housing increased by 40% in Europe [9] and by 13% in the United States of America [10]. The most current figure from the United Nations, dated 2024, suggests that globally, there are 2.8 billion people without access to adequate housing [11]. However, reliable data on the extent of homelessness in low- and middle-income countries remain scarce and fragmented [1, 2].

People experiencing homelessness (PEH) face multiple social and structural vulnerabilities that substantially increase their risk for a wide range of acute and chronic health conditions, including infectious diseases [12], cardiovascular illnesses [13], substance use disorders [14], and severe mental health disorders [15]. Mortality rates among PEH can be up to ten times higher than those reported in the housed population [16, 17]. Unmet health needs (reported by approximately 73% of PEH) and delayed care contribute to high rates of emergency department visits and avoidable hospitalizations, resulting in disproportionately high public healthcare expenditures [18, 19]. In the USA, the annual healthcare costs for individuals experiencing chronic homelessness can exceed $30,000 per person, largely because of fragmented and reactive care delivery [1, 20]. In addition to bearing a disproportionately high burden of disease, PEH often face multiple compounding barriers to healthcare utilization. Studies indicate that only around half of this population has regular access to health services, and less than 40% are covered by any form of health insurance [18, 21].

Although several healthcare interventions–defined as planned actions designed to improve health outcomes for a specific condition (e.g. therapeutic approaches, vaccinations)–, as well as support services such as shelters and mobile clinics have been implemented and described in the literature, evidence of their access and utilization remains limited and inconsistent [1, 22–24]. The complex interplay of individual, social, and structural factors such as financial constraints, substance use, and prior experiences of stigma and discrimination, in addition to fragile relationships with healthcare professionals, are recognized as potential barriers in healthcare. These factors often contribute to inefficiencies and unmet outcomes [25]. However, these factors are often addressed in isolation or within narrowly defined contexts–such as studies focusing on single interventions (e.g. vaccination) [26], on specific settings (e.g. emergency care) [27] or on social determinants and attitudes that may hinder willingness to use healthcare services [28], result in a fragmented understanding of the broader dynamics that affect healthcare access and utilization in this population.

Although some systematic reviews have investigated access-related issues within healthcare intervention, such as palliative care [29], otolaryngology [30], oral health [31], hepatitis C screening [32], and antenatal and postnatal care [3], their scope is typically limited to a single clinical area or service type. Moreover, some of these reviews were published over a decade ago, before major shifts in housing policy and service delivery for PEH, and may vary in methodological rigor (e.g. lack of protocol registration, incomplete search strategies, limited critical appraisal). As a result, literature still lacks an integrated and up-to-date synthesis of the cross-cutting factors that shape healthcare access and utilization across services for PEH.

To address this gap, we aim to systematically map the available evidence on healthcare access and utilization, whether delivered independently or as part of broader strategies promoting health and well-being for PEH, and identify the associated key barriers and facilitators.

## Methods

This systematic scoping review is part of the HOME Project, which aims to explore the barriers and facilitators that PEH face when accessing and utilizing healthcare. The review was conducted according to the Cochrane Collaboration [33] and Joanna Briggs recommendations [34] and reported following the Preferred Reporting Items for Scoping reviews (PRISMA-ScR) [35]. The study protocol was registered in the International Prospective Register of Systematic Reviews (PROSPERO) (CRD42025635835). All the main steps of this review, including study screening (title/abstract reading), full-text evaluation, and data extraction, were performed independently by two reviewers (D. G and F. S. T.); discrepancies were addressed through discussion involving a third reviewer (F.A.C).

### Search strategy and eligibility criteria

A systematic search of the PubMed, Embase, and Web of Science electronic databases (January 2025) was performed without restrictions on time or language. Descriptors related to the population (e.g. PEH) and healthcare access and utilization were combined with the Boolean operators AND and OR. See complete search strategies in Supplementary Material Table S1. Additionally, a manual search of the reference lists of the included studies and conventional search engines (i.e. Google) was conducted. The retrieved registers were organized into EndNote X21^®^, where duplicate records were removed.

Registers were then uploaded to the Rayyan^®^ web application [36] for screening (title and abstract reading) and eligibility (full-text reading) processes. Peer-reviewed primary scientific studies (interventional or observational studies of any design) that met all the following criteria (PCC [population, concept, context] acronym) were included for analysis:

- Population: studies including PEH - defined as marginalized or socially excluded groups/populations who lack a fixed, regular, secure and adequate night-time residence. This includes individuals who are unsheltered, as well as those living in shelters, other temporary housing and those living in insecure or inadequate housing [4–8].
- Concept: studies assessing general healthcare services delivered in different settings and with varying degree of urgency (e.g. primary care, routine medical consultations, and unplanned care, such as emergency care) and healthcare interventions (e.g. targeted disease management, mental health or substance-use treatment) targeting health and well-being among PEH, delivered independently or guided by health-related strategies or frameworks (i.e. preventive measures, treatment protocols, therapeutic approaches including palliative care, support services, educational and behavioral interventions);
- Context: Studies providing data on barriers and facilitators to accessing and utilizing healthcare interventions from the perspective of PEH in any clinical setting.

We excluded research involving individuals in permanent housing programs, as well as those that did not distinguish results for the target population or reported data from the perspective of health professionals. Pilot projects, study protocols, conference abstracts, proposals of untested intervention tools, and publications in non-Roman characters (e.g. Chinese) were also excluded.

### Data extraction and analysis

Standardized data collection forms (Microsoft Excel) were developed to extract data on study metadata (e.g. authors, year of publication, geographical region/country); participant characteristics (e.g. sample size, age, sex, clinical condition/diagnosis, shelter condition); type of intervention and controls (e.g. vaccinations, medications, educational programs); and main reported experiences in accessing and utilizing services (barriers/facilitators).

The findings from the included studies were narratively synthesized and presented in tables and graphs organized by intervention type and target population characteristics. The identified barriers and facilitators were classified according to the socioecological model, which comprises five levels: individual, interpersonal, institutional, community/organizational, and policy/structural [37–39]. At the individual level, barriers such as personal attributes, lived experiences, and health history were considered. The interpersonal level included barriers related to relationships with family, friends, and professionals. At the institutional level, the organization and standards of health and social services were included. The community/organizational level encompassed factors related to the organization of healthcare services and the availability of community resources. The policy/structural level addresses issues such as funding priorities, cultural norms, structural inequities, legal frameworks, and local/national strategic/operational plans. While recognizing the interaction between different levels, the classification considers the main domain.

Additionally, an evidence map was developed to synthesize the barriers and facilitators identified across the included studies and to associate the barriers and facilitators reported for each healthcare intervention. For analytical purposes, findings were grouped into broader thematic categories based on conceptual similarity. The thematic framework was developed inductively by the research team through iterative discussion and consensus seeking (Supplementary Material Table S2). For instance, issues such as the perception of insufficient space to express oneself and the perception of not being listened to were categorized as “communication” barriers. The reported numbers reflect the total frequency of specific mentions across studies; a single study could contribute multiple mentions to the same category if it reported different related barriers or facilitators. This approach offers a visual summary of the scope and depth of available evidence, helping to identify knowledge gaps and inform future research and policy development.

As this is a scoping review, a risk of bias assessment was not required and therefore was not conducted at this stage. Methodological guidelines primarily recommend focusing on mapping the breadth and nature of available evidence rather than appraising the quality of individual studies [34].

## Results

**Fig. 1:**
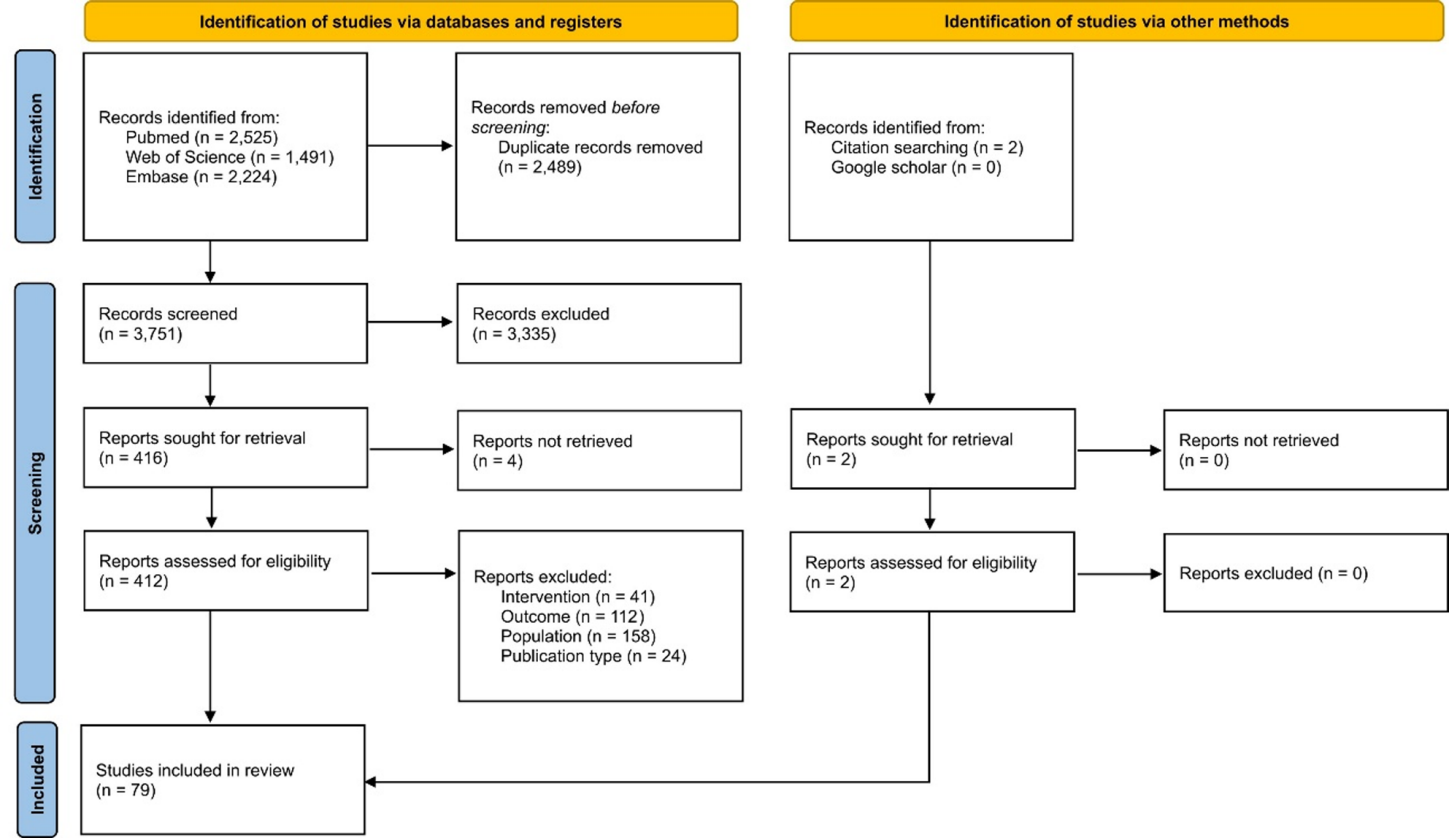
PRISMA 2020 flow diagram

The search yielded 3,751 records after duplicate removal. Of these, 3,335 were excluded during screening, leaving 416 records for full-text evaluation. Following full-text appraisal, four studies could not be retrieved, and 335 articles were excluded (see complete list of excluded studies with reasons for exclusion in Supplementary Material Table S3), resulting in 77 registers eligible for data extraction and analysis. Two additional records were identified through manual search, totaling 79 studies for synthesis. See Fig. 1 (complete list of included studies is available in Supplementary Material Table S4).

The included studies comprised a total of 5,110 PEH and were published between 1989 and 2024. Most studies were conducted in the United States of America (*n* = 48; 60.8%), followed by Canada (*n* = 10; 12.7%) and the United Kingdom (*n* = 10; 12.7%). A variety of study designs were employed, with most being of qualitative nature (*n* = 65; 82.3%). Across all the studies, 55.7% of the participants were female. The mean age of the participants, where reported, ranged from 15.6 to 59 years, with an overall mean of 40.8 years. The shelter’s status was reported inconsistently. Among the 35 studies that explicitly provided housing information, 55.2% of the participants (*n* = 2,820) were classified as unsheltered, whereas the remaining 44.8% were either sheltered or in transitional housing (see Supplementary Table S5 for detailed information).

**Fig. 2:**
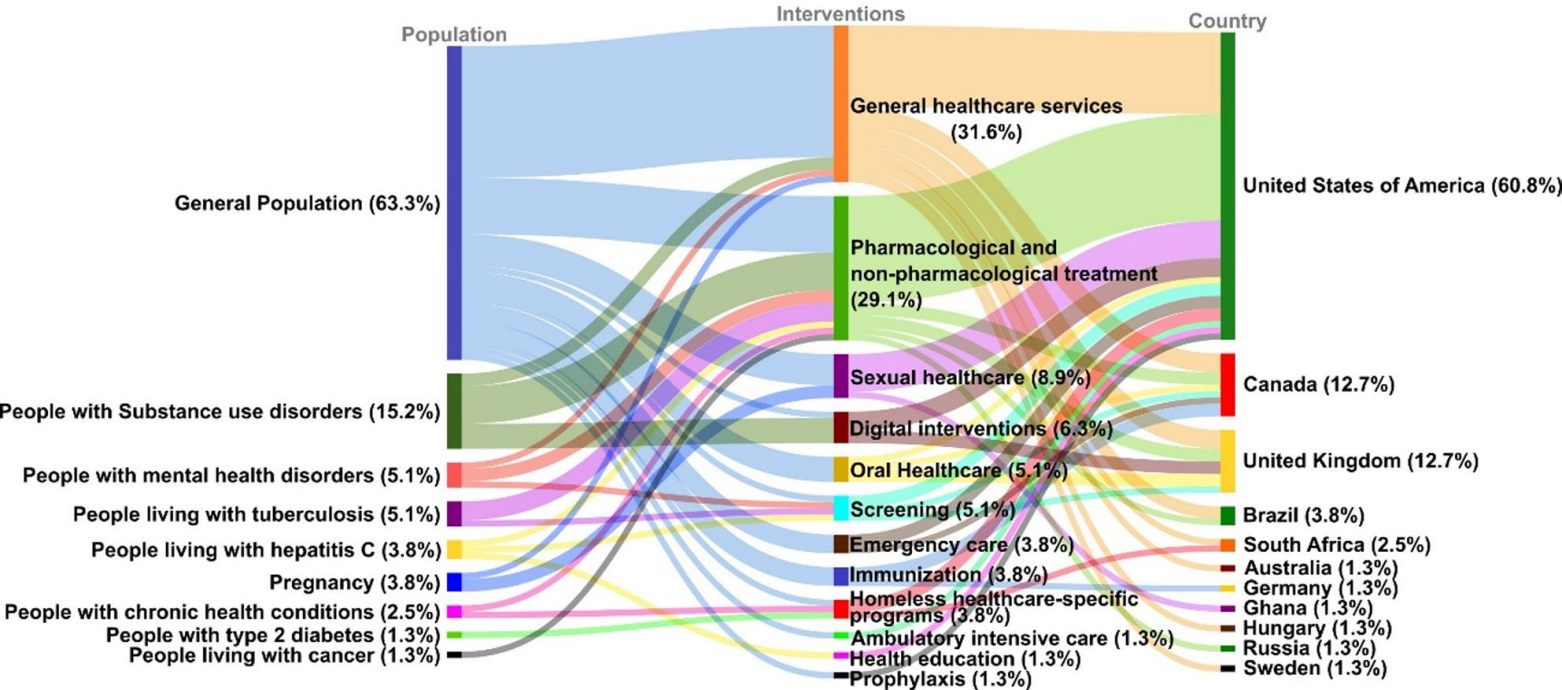
Health conditions and interventions of the included studies and the countries where they were conducted

With respect to the health conditions assessed, most studies (*n* = 50; 63.3%) did not restrict their evaluation to a specific diagnosis or clinical category; instead, they addressed health needs among the general population of PEH. Among the studies that targeted specific conditions, substance use disorders (including nicotine, alcohol and illicit drugs) were the most frequently assessed (*n* = 12; 15.2%), including four studies that focused specifically on tobacco use. Other conditions included infections (e.g. tuberculosis, human immunodeficiency virus [HIV], and viral hepatitis), mental health disorders, cancer and other conditions, such as pregnancy. General healthcare services were the most frequently studied healthcare interventions (*n* = 25; 31.6%), followed by pharmacological and non-pharmacological treatment (*n* = 23; 29.1%) and sexual and reproductive healthcare (*n* = 7, 8.9%) (see Fig. 2).

**Fig. 3:**
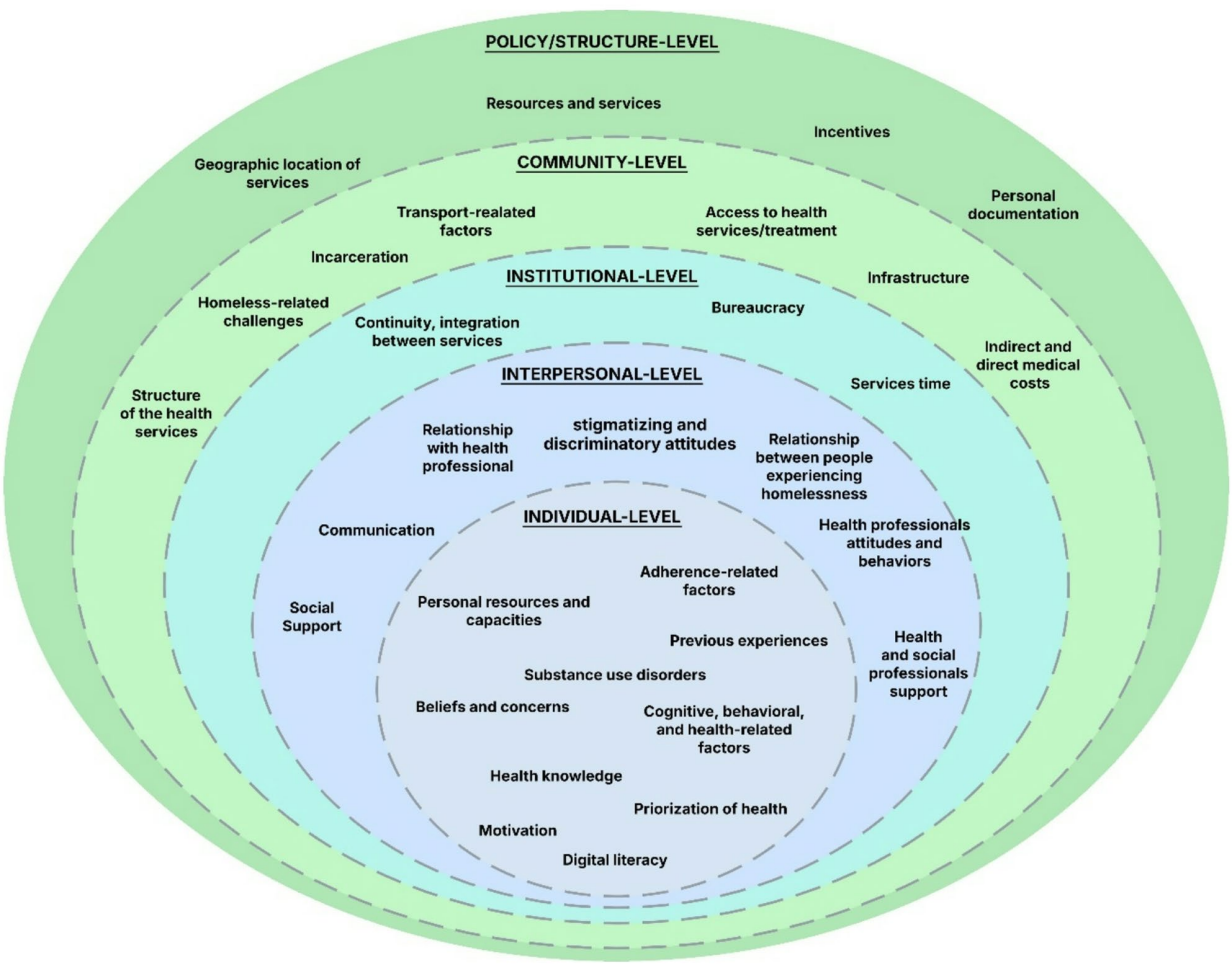
Socioecological model classification of the barriers and facilitators identified in the included studies

Most barriers and facilitators identified in the included studies were classified within the individual-level, followed by the interpersonal-level domain of the socioecological model (see Fig. 3; Tables 1 and 2; and full data in Supplementary Table S6).

**Table 1:** Evidence barrier mapping following the Socioecological model

		General healthcare services	Emergency care	Substance use disorders treatment	Text messaging smoking cessation	Computer-assisted therapy	MY- RID app	Health technology	Video telehealth	HCV screening and treatment	HCV education	PrEP	Adherence LTBI treatment	Tuberculosis screening	Tuberculosis treatment	Cancer screening	Otolaryngology care	Immunization	Mental health services	Psychosocial services	Contraception/reproductive care	Prenatal care	Oral health Care	Pain management	Medication management	Treatment navigation	Nurse-led mobile clinic	D-HOMES program	GPS-mHealth	Trinity Health Services (Student-run clinics)	Overall
Individual	Substance Use Disorder	5	1	1						1	2		1		1								2	2		1		1			18
	Health-related beliefs and concerns	5	2	4						1		1	2		1	2		7	5		4		2	2	5						42
	Cognitive, behavioral, health challenges	9	1	2		1		1	1	2	2					1					2		4	1	1			1			28
	Fear of bad news	2														1					1										4
	Previous negative experiences from using healthcare	2	2					1													1	1	1		1			1		1	11
	Lack of Motivation for health seeking behaviors	1						1															1								3
	Low prioritization of health	2	1	1								1	1						1		2		1	1							11
	Technology challenges					2		1	1					1															1		6
	Challenges with medication adherence	2										1							1		1				3						8
	Low health-related knowledge	5								1		1	1			1		1	1		2		1								14
Interpersonal	Negative attitudes (e.g. hostile or dehumanized)	18		2											2	1			2		2		2	1							29
	Difficulties in the relationship with health professionals	10	1	2						1			1						1			2	2	1		1		1			23
	Difficulties in communication	6	1										1	1	1			2													11
	Stigmatizing and discriminatory attitudes	25	2	1						1		2	1	1	1	1			1		3	1	6			1		1	1	2	50
	Lack of Social support	1		1																	3										5
	Lack of Healthcare and social Providers support	1		1		1			1									1			1			1		1					6
	Behaviors of university hospital staff																						2								2
	Difficult relationship between PEH	1		1																											2
Institutional	Bureaucracy	0	1	1						1								1			3	1	2				1				20
	Lack of Continuity and integration between services	9		1											1				2	1	1				1		2	1			18
	Long waiting time and intervention duration	7	1	1									1						1		1	1		1	1		1				15
Community	Lack of access to health service/treatment	5		2									1			1		2	3	2	1		1	2	2						22
	Structure of the health service	1			1		1																		2			1			6
	Infrastructures	1			2	4			2			2									1				3					1	16
	Direct medical costs*	15		2								2						1	3		1	1	3	1	2	1	2				**34**
	Indirect medical costs**	10	1	1												1	1		1			2		1	1		1				19
	Homelessness-related challenges	6										1	1		1				1				1	3	4			1			19
	Incarceration	1		1																											2
Policy/Structural	Insufficient resources and services	4											1								1							1			7
	Lack of public security				2		1	1														1			1				1		7
	Geographic location of service	1		1																										1	3
	Education																	1					1								2
	Lack of personal documentation	2														1		1													4

The classification of studies considered the main domain while acknowledging the interaction between the different levels, e.g. a policy may exist that will be reflected at an institutional level. The numbers represent the number of studies citing each specific barrier. * Direct costs include not having insurance and having difficulty paying for medication and other healthcare services; ** Indirect costs include difficulty paying for transportation to health services; D-HOMES: Diabetes HOmeless MEdication Support; GPSmHeath: GPS mobile health apps (mobile application that monitors geofences of PEHs and notifies health professionals of hospital entries, allowing follow-up after the visit); HCV: hepatitis C virus; MY-RID: Motivating Youth to Reduce Infection and Disconnection; PrEP: pre-exposure prophylaxis

**Table 2:** Evidence facilitators mapping following the Socioecological model

		General healthcare services	Emergency care	Ambulatory Intensive Care	Substance use disorders treatment	Text messaging smoking cessation	Computer-assisted therapy	MY- RID app	Health technology	Video Telehealth	HCV education	HCV screening and treatment	PrEP	Tuberculosis treatment	Adherence for LTBI treatment	Tuberculosis screening	Cancer screening	Otolaryngology care	Immunization	Mental health services	Contraception/reproductive care	Prenatal care	Oral health Care	Medication	Treatment navigation	Nurse-led mobile clinic	Nurse-led outreach healthcare	D-HOMES program	GPS-mHealth	Trinity Health Services (Student-run clinics)	Overall
Personal	Personal resources, capacities	3	1		1		1												1		2			2							11
	Emotional, psychological states	1	1		1		1																						1		4
	Health-related beliefs and perceptions	3																	2					3							7
	Health knowledge	4			2						1	2			1		1		1	1	1			2							15
	Motivation, perceived benefits	2																						2				1			5
	Previous experiences											1					1														2
	Not having any addictions																							1							1
Interpersonal	Good communication with the providers	5	1	1		1									1	1					1					1					11
	Social relationships, support	11	1	1	3		1			1	1		5	2			1		1					2		2	1		2		33
	Health professionals’ attitudes and characteristics	17	1	4	1										2								2			1	1	1		3	33
	Relationship with health professionals	7		1	1									1	1					1	1	1				1					14
	Labeling identity	1																													1
Institutional	Bureaucracy	1					3												1				1			1					7
	Continuity, integration between services	5													1													2			7
	Timely service delivery	3					1								1											1		1			7
Community	Access to service/treatment	3		1	1							1	2				1				1			1		2	1				14
	Structure of the health service	3	1				4	1		1			1		1			1			1				2						16
	Transport support	2																1													3
	Infrastructures								1																						1
Policy/Structural	Resources and services																1				1									1	3
	Geographic location of service	8			2								1		1								1	1			1				14
	Incentives											1		1	1						1										4

The classification of studies adopted valued the most prominent domain while acknowledging the interaction between the different levels, e.g. a policy may exist that will be reflected at an institutional level. The numbers represent the number of studies citing each specific facilitator. D-HOMES: Diabetes HOmeless MEdication Support; GPSmHealth: GPS mobile health apps (mobile application that monitors the geofences of PEHs and notifies health professionals of hospital entries, allowing follow-up after the visit); HCV: hepatitis C virus; MY-RID: Motivating Youths to reduce infection and disconnection; PrEP: pre-exposure prophylaxis

Among the individual-level barriers to accessing and utilizing healthcare, beliefs and concerns (*n* = 42) were frequently identified, particularly with respect to medication safety (*n* = 15), perceived efficacy (*n* = 10), and mistrust in healthcare institutions or providers (*n* = 8). Substance use disorders (*n* = 18) were also one of the most frequent individual challenges. Among interpersonal barriers, stigmatizing and discriminatory attitudes (*n* = 50) were the most frequently reported, including experiences of stigma (*n* = 21), discrimination (*n* = 9), and moral judgment (*n* = 9), often arising in interactions with health professionals and services. Other interpersonal barriers involved health professionals’ attitudes and behaviors (*n* = 29), including disrespect (*n* = 6), lack of empathy (*n* = 4), disinterest (*n* = 4), and negligent care (*n* = 4). At the institutional level, barriers were primarily related to bureaucratic processes (*n* = 20) and lack of continuity of care (*n* = 18). Community-level barriers included limited access to services (*n* = 22), homelessness-related challenges (*n* = 19), direct medical costs (such as lack of insurance [*n* = 13] and payment difficulties [*n* = 21]), and indirect medical costs related to transportation (*n* = 19). At the policy level, the most frequently cited barriers were insufficient resources and services (*n* = 7) and lack of documentation (*n* = 4). Individual-level facilitators included health knowledge (*n* = 13) and personal resources and capabilities (e.g. motivation, self-efficacy) (*n* = 11). The most frequently reported facilitators at the interpersonal level were related primarily to health professionals’ attitudes and characteristics (*n* = 33) (including respectful approaches and comprehensive or holistic care) and to social relationships and support networks (*n* = 33). Institutional-level facilitators included simplified administrative processes (*n* = 7), continuity and integration of healthcare services (*n* = 7), and timely service delivery (*n* = 7), which included short waiting times (*n* = 4), brief intervention duration (*n* = 2), and appointment reminders (*n* = 1). At the community level, the organization and structure of health services were recurrent themes (*n* = 16), often linked to the use of health technology tools such as virtual care (*n* = 4), improved service availability (*n* = 3) and user-friendly digital interfaces (*n* = 3). Digital interventions were identified as barriers in some studies, as they required a certain level of digital literacy. However, other studies reported them as facilitators, given the capacity of telehealth to increase access and utilization and overcome transportation barriers (*n* = 4). At the policy level, key facilitators included aspects of service location and accessibility, influenced by legislative measures (*n* = 14), with a focus on geographic proximity and easily accessible sites (*n* = 7), as well as on-site care delivery models (*n* = 5).

## Discussion

This scoping review, which included 79 studies of various designs over three decades, comprehensively mapped the available evidence on barriers and facilitators for PEH to access healthcare. It also identified the complex, multilevel barriers and facilitators shaping healthcare engagement among PEH, underscoring the importance of context specific, person-centered approaches. These findings align with previous systematic reviews highlighting the diversity of healthcare approaches targeting PEH, particularly in high-income settings where complex interventions, such as integrated primary care models (e.g. H-PACT), mobile health clinics, Housing First programs (providing permanent housing and support services), and harm reduction strategies, are commonly implemented [2, 25]. Multidisciplinary and context-adapted strategies are generally considered more effective in addressing the multifaceted needs of marginalized and vulnerable populations [40–43].

These reviews highlight that the most frequently reported barriers to intervention use among PEH occur at the interpersonal and individual levels. These findings align with previous research showing that PEH face significant stigma when accessing healthcare and social care services, which not only reduces engagement but is also associated with poorer physical and mental health outcomes [28, 44]. A systematic review found that perceived stigma was associated with a 44% lower likelihood of using primary care services and a 30% higher risk of mental distress among PEH [28]. Another meta-analysis reported that approximately 70% of individuals experiencing homelessness described their interactions with healthcare professionals as negative or dismissive, discouraging further care-seeking behavior [44]. Stigma and marginalization also exacerbate difficulties in meeting basic needs (e.g. food, water, employment, and housing), contributing to chronic economic instability and reinforcing the vicious cycle of homelessness [45]. Supporting this, the *Fédération Européenne d’Associations Nationales Travaillant avec les Sans-Abri* (FEANTSA) [European Federation of National Organizations Working with the Homeless] reported that over 70% of individuals experiencing long-term homelessness lack access to a general practitioner or basic healthcare coverage [9]. Limited access often leads to overuse of emergency care, which was mentioned in several studies but rarely examined as a primary focus.

Positive attitudes by healthcare professionals (including a respectful, compassionate, and nonjudgmental approach) are recognized as facilitators of interventions’ access and use [28, 44]. Previous studies have shown that the implementation of trauma-informed care models, psychosocial services, and permanent supportive housing programs is associated with improved interpersonal interactions and individual health behaviors, including a 25–40% increase in follow-up appointment attendance, reductions in hospital readmissions, and improved medication adherence among marginalized populations [46–48]. Moreover, qualitative evidence indicates that PEH often value consistency, autonomy, and feeling ‘seen and heard’ by providers, elements rarely captured in adherence-focused outcomes [22, 44]. These findings highlight the importance of incorporating lived experience and user feedback into the design and evaluation of healthcare interventions for PEH, as promoted by participatory research frameworks.

Many of the barriers reported in this review also have a political dimension. These structural barriers, consistently identified as impediments to healthcare equity and effective service delivery, are illustrated by data showing that 20–25% of low-income individuals without vehicle access miss medical appointments. Qualitative studies among unsheltered populations further highlight frequent missed visits caused by the high cost and unreliability of public transport [49–51]. Financial constraints also significantly reduce healthcare utilization, with many PEH and low-income individuals delaying or foregoing care because they cannot afford copayments, medications, or related expenses. In the US, more than 30% of uninsured low-income adults avoid clinician visits because of associated costs [52, 53]. Additionally, uninsured people tend to experience lower rates of preventive care and higher rates of emergency department use. Studies show that, compared with their insured counterparts, uninsured PEH are twice as likely to report unmet medical needs [16, 54]. Moreover, a survey in North America also found that the absence of personal identification or a health card limits access to primary care services among PEH [55], a barrier that could be mitigated through accessible identification support programs. Some of these barriers may be less context- specific than others. For example, many countries in Western Europe have a Beveridge healthcare model, in which all citizens and residents have access to healthcare at the point of delivery without the need for insurance. Nevertheless, lack of documentation is considered a more universal barrier and is likely to be exacerbated in the coming years given recent political trends.

In this scenario, factors closely aligned with these barriers included social relationships and support networks, health knowledge and literacy, and the accessibility and organization of healthcare services and treatments. Robust social support is associated with greater healthcare engagement, with PEH being up to 2.5 times more likely to attend scheduled medical appointments than those receiving usual care or without social support [56, 57]. Conversely, poor health literacy reduces the effectiveness of behavior change interventions, impairs disease management, and limits understanding of prevention and treatment [58, 59]. Tailored interventions that simplify health information are therefore essential for PEH and may improve self-rated health. Shelters and support agencies can provide practical health literacy education and navigation programs to support this population [58].

Financial and nonfinancial incentives have also emerged as powerful facilitators to improve healthcare adherence. During the COVID-19 pandemic, programs offering monetary incentives increased vaccine uptake by 15–20% among homeless populations [60, 61]. Similarly, the use of food vouchers in tuberculosis treatment improved completion rates from 55% to over 75% in marginalized groups [62, 63]. These findings highlight the importance of combining enhanced social support and education with strategically designed incentives to overcome structural and individual barriers, thereby optimizing healthcare engagement and treatment success in PEH. Recently, local initiatives have been developed to address living instability and transportation challenges. These initiatives aim to improve access to treatment by providing psychiatric assessments and institutionalization when appropriate and dedicated primary care appointments. They actively reach PEH throughout the city and are led collaboratively by municipalities, academic institutions, volunteers, and civil society organizations, exemplifying community-level facilitators [64].

Future interventions should adopt integrated care delivery models tailored to the unique needs of PEH. Evidence suggests that ‘low-barrier’ clinics, offering walk-in services, minimal paperwork, and harm-reduction support, significantly increase engagement among highly marginalized populations [40, 43]. Similarly, “one-stop-shop” and drop-in models that collocate psychiatric services, cognitive behavior interventions, and social services have been shown to reduce missed appointments and improve care continuity [65]. The deployment of mobile health units, particularly when combined with technological and digital tools such as georeferencing apps, short message service [SMS] reminders, and navigation systems, can overcome logistical and geographic access barriers [66–68]. These healthcare delivery models offer cost savings and improved access not only for PEH but also for other marginalized populations, addressing broader health inequalities [69]. Policymakers and implementers should prioritize scalable and flexible delivery frameworks that integrate these features. However, our review reveals that current health interventions remain fragmented, heterogeneous in design (e.g. varying intervention duration, clinical settings, and outcome measures), are often limited in scope, duration and follow-up engagement. These findings reinforce the need for robust public health policies and cross-sectoral frameworks to ensure continuity of care and facilitate the scale-up of successful models. National strategies, such as the pathway model in the United Kingdom and Housing First programs in Canada and Portugal, illustrate the potential of integrated and alternative approaches to addressing homelessness and health inequalities [70, 71]. The pathway model improves care coordination in hospitals by supporting housing referrals and reconnecting people with primary care services [71]. In contrast, the Housing First model reverses the traditional treatment-first approach, particularly in the context of PEH with drug use disorders, by providing immediate and unconditional housing, employment opportunities, and access to health and social services, ultimately promoting reintegration [70].

This review has several limitations that should be considered when interpreting its findings. The included studies have substantial heterogeneity in their design and implementation. Variations were observed in intervention types (e.g. one-time services vs. longitudinal programs), target populations (e.g. PEH with general health conditions vs. PEH with specific conditions), and clinical settings (e.g. shelters, emergency departments, mobile units). Furthermore, most studies were conducted in the United States, whose social, economic, and healthcare context differs from that of other countries. For example, the U.S. healthcare system differs from universal, government-funded systems, and despite high per-capita healthcare spending, the country consistently underperforms on a range of health outcomes compared with other Organization for Economic Cooperation and Development (OECD) nations. Methodological inconsistencies, including the use of unvalidated or poorly described data collection instruments, unclear definitions of key outcomes (e.g. adherence, engagement) and subgroups of PEH and inconsistent reporting of demographic characteristics, further challenge the synthesis and generalizability of the results.

These issues highlight the need for standardized research protocols, validated outcome measures, and transparent reporting practices in studies evaluating health interventions for PEH. Given the scoping nature of this review, the primary aim was to map and categorize existing evidence rather than to assess study quality; consequently, no formal risk of bias assessment was conducted. Future systematic reviews and meta-analyses should build on these findings through rigorous evaluation of intervention effectiveness.

## Conclusion

Our study suggests that healthcare inaccessibility among PEH is not primarily a clinical issue but largely reflects systemic design and policy shortcomings. While individual-level factors such as substance use and health beliefs influence care engagement, the most frequently reported and actionable barriers identified in this review were interpersonal (e.g. stigma and negative provider attitudes) and structural (e.g. administrative complexity, fragmented services, financial constraints, lack of documentation, and geographic inaccessibility). Addressing interpersonal barriers requires sustained investment in trauma-informed and stigma-sensitive training across healthcare education and practice. However, structural barriers demand policy-level reform, including strengthened interministerial collaboration to integrate health and social services, system redesign to simplify administrative processes and improve care continuity, and targeted social measures to reduce financial obstacles (e.g. free preventive care and transport support). Digital tools may support these reforms, provided they are co-designed with people with lived experience, and complemented by in-person navigation. Sustainable improvement in healthcare access for PEH therefore depends on coordinated structural reform rather than isolated clinical interventions.

## Supplementary Information

Below is the link to the electronic supplementary material.

Supplementary Material 1

Supplementary Material 2

## Data Availability

No additional data are available.

## Abbreviations

COVID-19: Corona virus disease 2019
D-HOMES: Diabetes HOmeless MEdication Support
FEANTSA: Fédération Européenne d’Associations Nationales Travaillant avec les Sans-Abri
GPS: Global Positioning System
GPSmHealth: GPS mobile health apps
HBV: Hepatitis B virus
HCV: Hepatitis C virus
HIV: Human immunodeficiency virus
MY-RID: Motivating Youth to Reduce Infection and Disconnection
OAT: Opioid Agonist Treatment
OECD: Organization for Economic Cooperation and Development
PCC: Population, concept, context
PEH: People experiencing homeless
PrEP: Pre-exposure prophylaxis
PRISMA-ScR: Preported following the Preferred Reporting Items for Scoping reviews
PROSPERO: International Prospective Register of Systematic Reviews
PSH: Permanent Supportive Housing
SMS: Short Message Service
USA: United States of America
