# Supplementary material for: Barriers to and facilitators of healthcare access among people experiencing homelessness: a systematic scoping review
Source: Int J Equity Health. 2026 Apr 3;25:99. doi: 10.1186/s12939-026-02840-z (PMC13077930; doi:10.1186/s12939-026-02840-z)
Supplement: Supplementary file 1 — Supplementary Material 1 [file 12939_2026_2840_MOESM1_ESM.docx]

**Supplementary Material**

**Barriers and facilitators to healthcare access among people experiencing homelessness: a systematic scoping review**

**Table S1. Search strategy**

| **Pubmed** | |
| --- | --- |
| #1 “Ill-Housed Persons”[MH] OR homeless*[TIAB] OR houseless[TIAB] OR “street people”[TIAB] OR unhoused[TIAB] OR roofless*[TIAB] OR shelterless*[TIAB] OR unshelter*[TIAB]  AND  #2 therapy[TIAB] OR therapies[TIAB] OR intervention*[TIAB] OR service*[TIAB] OR treatment*[TIAB] OR program*[TIAB] OR initiative*[TIAB] OR campaign*[TIAB] OR prevention[TIAB] OR preventive[TIAB] OR screening[TIAB] OR support[TIAB] OR aid[TIAB] OR “point of care”[TIAB] OR emergency[TIAB] OR urgent[TIAB] OR vaccin*[TIAB] OR immunization[TIAB] OR immunization[TIAB] OR care[TIAB] OR healthcare[TIAB]  AND  #3 “Health Services Accessibility”[MH] OR coverage[TIAB] OR access*[TIAB] OR utilization[TIAB] OR utilization[TIAB] OR acceptance[TIAB] OR adherence[TIAB] OR engagement[TIAB] OR acceptability[TIAB] OR barrier*[TIAB] OR facilitator*[TIAB]  AND  #4 “clinical trials as topic”[MH] OR “clinical trial”[TIAB] OR “clinical study”[TIAB] OR control*[TIAB] OR random*[TIAB] OR nonrandom*[TIAB] OR “cohort studies”[MH] OR cohort[TIAB] OR “case-control studies”[MH] OR case-control[TIAB] OR “real world”[TIAB] OR observational[TIAB] OR “before and after”[TIAB] OR “quasi-experimental”[TIAB] OR “pre-post”[TIAB] OR registry[TIAB] OR longitudinal[TIAB] OR prospective[TIAB] OR retrospective[TIAB] OR qualitative[TIAB] | 2,525 |
| **Embase** | |
| (homeless*:ab,ti OR houseless:ab,ti OR 'street people':ab,ti OR unhoused:ab,ti OR roofless*:ab,ti OR shelterless*:ab,ti OR unshelter*:ab,ti) AND (therapy:ab,ti OR therapies:ab,ti OR intervention*:ab,ti OR service*:ab,ti OR treatment*:ab,ti OR program*:ab,ti OR initiative:ab,ti OR campaign:ab,ti OR prevention:ab,ti OR preventive:ab,ti OR screening:ab,ti OR support:ab,ti OR aid:ab,ti OR 'point of care':ab,ti OR emergency:ab,ti OR urgent:ab,ti OR vaccin*:ab,ti OR immunization:ab,ti OR healthcare:ab,ti) AND (coverage:ab,ti OR access*:ab,ti OR utilization:ab,ti OR acceptance:ab,ti OR adherence:ab,ti OR engagement:ab,ti OR acceptability:ab,ti OR barrier*:ab,ti OR facilitator*:ab,ti) AND (('clinical trial':ab,ti OR 'clinical study':ab,ti OR control*:ab,ti OR random*:ab,ti OR nonrandom*:ab,ti OR cohort:ab,ti OR 'case control':ab,ti OR 'real world':ab,ti OR observational:ab,ti OR before:ab,ti) AND after:ab,ti OR 'quasi-experimental':ab,ti OR 'pre-post':ab,ti OR registry:ab,ti OR longitudinal:ab,ti OR prospective:ab,ti OR retrospective:ab,ti OR qualitative:ab,ti) | 1,491 |
| **Web of science** | |
| homeless* OR homeless OR “street people” OR unhoused OR roofless* OR shelterless* OR unshelter* (Title) and therapy OR therapies OR intervention* OR service* OR treatment* OR program* OR initiative OR campaign OR prevention OR preventive OR screening OR support OR aid OR “point of care” OR emergency OR urgent OR vaccin* OR immunization OR immunization OR healthcare (Topic) and coverage OR access* OR utilization OR acceptance OR adherence OR engagement OR acceptability OR barrier* OR facilitator* (Topic) and “clinical trial” OR “clinical study” OR control* OR random* OR nonrandom* OR cohort OR case-control OR “real world” OR observational OR “before and after” OR “quasi-experimental” OR “pre-post” OR registry OR longitudinal OR prospective OR retrospective OR qualitative (Topic) | 2,224 |

**Table S2. Thematic Framework of Barriers and Facilitators**

| **Personal level** | **Interpersonal level** | **Institutional level** | **Community level** | **Policy/structural level** |
| --- | --- | --- | --- | --- |
| **barriers** | | | | |
| - **Substance Use Disorder:** use of cigarettes, alcohol, and illegal substances. - **Health Beliefs and concerns:** beliefs about health, treatment, healthcare services, and institutions or personal beliefs. - **Cognitive, behavioral, health challenges:** health, cognitive, and behavioral conditions, such mental health issues, emotional challenges, and embarrassment - **Fear of bad news:** People fear going to health services for fear of discovering other health problems or interventions. - **Previous negative experiences from using healthcare:** history of negative prior experiences with medications or misdiagnosis. - **Lack of Motivation for health seeking behaviors:** lack of motivation or interest in seeking health services or managing health conditions or feeling frustrated with these services. - **Low prioritization of health:** lack of prioritization of health over other needs - **Technology challenges:** Limited digital literacy or lack of trust in health technologies. - **Challenges with medication adherence:** Problems with adherence, such as forgetting to take medication. - **Low health-related knowledge:** There is a lack of knowledge about health interventions and conditions, and a lack of understanding of the role of their medications. | - **Health professionals’ attitudes and behaviors:** Negative attitudes (e.g., hostility, negligence, or disrespect) or personal characteristics of providers (e.g., sex or age). - **Difficulties in the relationship with health professionals:** such lack of trust or fear of a lack of confidentiality. - **Difficulties in communication:** Issues with the language, lack of explanations, and speaking place. - **Stigmatizing and discriminatory attitudes:** Stigma, discrimination, disregard, exclusion, treatment, judgment, prejudice, and stereotypes. - **Lack of Social Support:** Lack of family and friend support. - **Lack of health and social professional’s support** - **Behaviors of university hospital staff:** Attended by health professors and students - **Difficult relationship between** **homeless** | - **Bureaucracy:** of health services and shelters, including logistics for access, eligibility criteria, the need to fill out multiple forms, restrictions on take-home doses, and vaccination policy. - **Lack of continuity, integration between services:** Such continuity with the same health professional, coordination between health and social services, and coordination between health services. Barriers to navigating the health system and wanting to return to specific providers. - **Long waiting and intervention duration:** long waiting time, time necessary for appointments, and time is needed for intervention. | - **Lack of access to health service/treatment:** lack of access to or scarcity of health services, medication, or health education programs. - **Structure of the health service:** Lack of tailored interventions and intervention structure and content. - **Infrastructures:** Such challenges include problems with storing medicines, internet access, cell phone charger availability, physical need satisfaction, privacy, and limited computer access. - **Direct medical cost:** such lack of healthcare insurance and health payment challenges - **Indirect medical cost:** The cost of necessities, transportation, and healthcare, - **Homeless-related challenges:** Transition or unstable situations, stress from shelter life, or lack of resources for basic needs. - **Incarceration:** History of recent incarceration or frequent incarcerations. | - **Insufficient resources and services:** That included the quality of health services, lack of incentives, few free services, lack of facilities, lack of available resources, and lack of research inclusion. - **Issues in public security:** including stolen and thefts. Criminal laws - **Geographic location of service:** lack of proximity to services, Distances to Services and Travel Time - **Education issues:** school education. - **Lack of personal documentation:** lack of identification documents is required to schedule appointments. |
| **Facilitators** | | | | |
| - **Personal resources, capacities:** Access to basic needs, such as food, water, and shelter; stable housing; a consistent daily routine; familiarity with the intervention; carrying medications with you; taking medications with food or water; and the ability to navigate digital tools. - **Emotional, psychological states:** Improve well-being, overcome the fear of health condition consequences, and feelings of self-worth - **Health-beliefs and perceptions:** Beliefs about the consequences of continuity of care and of not receiving healthcare, health interventions, religious beliefs, high-risk perception of the condition, and understanding the importance of health services or interventions. - **Health knowledge:** Knowledge of interventions/medications, and healthcare services. - **Motivation, perceived benefits:** Personal motivation for health reasons, such as the desire to recover or the perception of symptom relief or the benefits of medication, as well prevent transmission to others - **Previous experience:** Good previous experience with the health service. - **Not having any addictions:** Not having a problem with drinking | - **Good communication with health professionals:** good communication with health professionals, use of accessible language, and active listening by professionals or support from translators. - **Social relationships, support:** family, friends and provider’s support. - **Health professionals attitudes:** Positive attitudes include a nonjudgmental, empathetic, proactive, respectful, and comprehensive approach, as well as personal characteristics such as professional knowledge. - **Relationship with professionals**: a good relationship between PEH and professionals and building trust. - **Labelling identity** | - **Bureaucracy:** de-bureaucratization services, such no forms, flexibility, facilitated registrations, and single appointment for treatment. - **Continuity, integration between services:** Integration between social and health services, as well as between health services, allows providers to deliver more comprehensive care and ensure continuity. - **Timely delivery service:** little waiting time, and time is needed for intervention and appointment reminders. | - **Access to service/treatment:** accessible and free health services and medications. - **Structure of the health service:** alternative or nontraditional services, personalized services, convenience of the services, and health technologies. - **Transport support:** transportation support - **Infrastructures:** free wi-fi | - **Resources and services:** Access to shelters and social services. - **Services locations:** Accessible/proximity locations, centralized services care located on-site. - **Geographic location of service:** incentives for health care, such financial and food incentives |

**Table S3. Excluded studies after full-text reading with reasons for exclusion**

| **Reference** | **Reason** |
| --- | --- |
| Tadros, A. and Layman, S. M. and Brewer, M. P. and Davis, S. M..A 5-year comparison of ED visits by homeless and nonhomeless patients.Adm Policy Ment Health.2016;34(5):805-8. | No outcomes reported |
| Sajatovic, M. and Ramirez, L. F. and Fuentes-Casiano, E. and Cage, J. and Tatsuoka, C. and Aebi, M. E. and Bukach, A. and Cassidy, K. A. and Levin, J. B..A 6-Month Prospective Trial of a Personalized Behavioral Intervention + Long-Acting Injectable Antipsychotic in Individuals With Schizophrenia at Risk of Treatment Nonadherence and Homelessness.Women Health.2017;37(6):702-707. | No outcomes reported |
| Russolillo, A. and Moniruzzaman, A. and Parpouchi, M. and Currie, L. B. and Somers, J. M..A 10-year retrospective analysis of hospital admissions and length of stay among a cohort of homeless adults in Vancouver, Canada.Ann Surg Oncol.2016;16():60. | No outcomes reported |
| Sivertsen, N. and Parry, Y. and Willis, E. and Kendall, S. and Marriott, R. and Bell, A..Aboriginal children and family connections to primary health care whilst homeless and in high housing mobility: observations from a Nurse Practitioner-led service.Can J Diabetes.2022;23():. | No outcomes reported |
| Corker, E. and Lorencatto, F. and Anderson, N. and Gobin, M. and Scott, S. and Michie, S. and Angel, G..Acceptability and facilitators of and barriers to point-of-care HIV testing in a homeless-focused service in Gloucestershire: a qualitative evaluation.Qual Health Res.2022;23(3):237-248. | Population |
| Kibel, M. and Shah, P. and Ayuku, D. and Makori, D. and Kamaara, E. and Choge, E. and Nyairo, J. and Abuya, P. and Wahome, M. and Wachira, J. and Braitstein, P..Acceptability of a Pilot Intervention of Voluntary Medical Male Circumcision and HIV Education for Street-Connected Youth in Western Kenya.Health Soc Care Community.2019;64(1):43-48. | Population |
| Reid, N. and Buchman, D. and Brown, R. and Pedersen, C. and Kozloff, N. and Stergiopoulos, V..The acceptability of financial incentives to support service engagement of adults experiencing homelessness and mental illness: a qualitative study of key stakeholder perspectives Authorship.Open Forum Infect Dis.2022;49(6):1060-1071. | Population |
| Schiffler, T. and Carmichael, C. and Smith, L. and Doñate-Martínez, A. and Alhambra-Borrás, T. and Varadé, M. R. and Barrio Cortes, J. and Kouvari, M. and Karnaki, P. and Moudatsou, M. and Tabaki, I. and Gil-Salmeron, A. and Grabovac, I..Access to cancer preventive care and program considerations for people experiencing homelessness across four European countries: an exploratory qualitative study.Arch Intern Med.2023;62():102095. | Population |
| Hadland, S. E. and Kerr, T. and Li, K. and Montaner, J. S. and Wood, E..Access to drug and alcohol treatment among a cohort of street-involved youth.Aids.2009;101(1):1-7. | Population |
| Howard, A..Access to health services for people experiencing homelessness in Manchester, UK: a qualitative study.Journal of Social Distress and the Homeless.2024;74():. | Publication type |
| Wenzel, S. L. and Audrey Burnam, M. and Koegel, P. and Morton, S. C. and Miu, A. and Jinnett, K. J. and Greer Sullivan, J..Access to inpatient or residential substance abuse treatment among homeless adults with alcohol or other drug use disorders.Community Dent Oral Epidemiol.2001;39(11):1158-69. | No outcomes reported |
| de Veer, A. J. E. and Stringer, B. and van Meijel, B. and Verkaik, R. and Francke, A. L..Access to palliative care for homeless people: complex lives, complex care.Am J Public Health.2018;17(1):119. | Population |
| Bellen, B. A. and Leyba, K. and Johnson, A. V. and Grimm, E. and Bredenberg, E..Access to palliative care for individuals with cancer experiencing homelessness.Journal of Clinical and Translational Science.2024;33(9):. | Publication type |
| McGuire, J. and Gelberg, L. and Blue-Howells, J. and Rosenheck, R. A..Access to primary care for homeless veterans with serious mental illness or substance abuse: a follow-up evaluation of co-located primary care and homeless social services.J Res Pharm Pract.2009;36(4):255-64. | No outcomes reported |
| Silver, C. M. and Janczewski, L. M. and Royan, R. and Chung, J. W. and Bentrem, D. J. and Kanzaria, H. K. and Stey, A. M. and Bilimoria, K. Y. and Merkow, R. P..Access, Outcomes, and Costs Associated with Surgery for Malignancy Among People Experiencing Homelessness.Harm Reduct J.2024;31(3):1468-1476. | No outcomes reported |
| McSweeney, B. and Campbell, R. B. and Grewal, E. K. and Booth, G. L. and Tariq, H. and Campbell, D. J. T..Accessing Diabetes Specialty Care for Persons With Lived Experience of Homelessness in Canada: Challenges and Opportunities.Circ Cardiovasc Qual Outcomes.2022;46(6):594-601.e2. | Population |
| Christian, N. and McFall, C. and Suarez, J. and Ulack, C. and Wagen, B. and Williams, W. and Teisberg, E..Achieving Calm: A Study on the Health Care Experiences of People With Lived Experience of Homelessness in Central Texas.Ambul Pediatr.2022;32(14):2118-2125. | Population |
| Hutt, E. and Albright, K. and Dischinger, H. and Weber, M. and Jones, J. and O'Toole, T. P..Addressing the Challenges of Palliative Care for Homeless Veterans.AIDS Care.2018;35(3):448-455. | Population |
| Caban-Aleman, C. and Iobst, S. and Luna, A. M. and Foster, A..Addressing the Poverty Barrier in Collaborative Care for Adults Experiencing Homelessness: A Case-Based Report.PLoS One.2020;56(4):652-661. | No outcomes reported |
| Moczygemba, L. R. and Osborn, R. D. and Lapane, K. L..Adherence to behavioral therapy and psychiatry visits in a safety-net setting in Virginia, USA.Soc Work Public Health.2014;22(5):469-78. | Population |
| Tulsky, J. P. and Pilote, L. and Hahn, J. A. and Zolopa, A. J. and Burke, M. and Chesney, M. and Moss, A. R..Adherence to isoniazid prophylaxis in the homeless: a randomized controlled trial.Aten Primaria.2000;160(5):697-702. | Population |
| Chau, K. M. and Norris, J. and Heyworth, L..Advance care planning amongst the homeless: Perceptions of the physician orders for life sustaining treatment (POLST) form and barriers to its usage in the outpatient setting.J Addict Med.2018;33(2):107-108. | Publication type |
| Thurman, W. and Heitkemper, E. and Hutson, T. and Sinay, K. L. and Glaeser, H. and Trice, E. and Kidane, H. and Dolotina, I..Analyzing the CTOSH listserv to understand the needs, barriers, resources, and strategies used by homeless service providers in central Texas.Int J Equity Health.2024;100():. | Population |
| O'Toole, T. P. and Buckel, L. and Bourgault, C. and Blumen, J. and Redihan, S. G. and Jiang, L. and Friedmann, P..Applying the chronic care model to homeless veterans: effect of a population approach to primary care on utilization and clinical outcomes.J Palliat Med.2010;92(12):2493-9. | No outcomes reported |
| Santiago, R. E. and Wisniewski, J..Assessing Barriers to Retention in Care Continuum Among HCV Positive Homeless Adults of New Orleans.Advances in Dual Diagnosis.2020;19():123. | Publication type |
| Parkes, T. and Matheson, C. and Carver, H. and Foster, R. and Budd, J. and Liddell, D. and Wallace, J. and Pauly, B. and Fotopoulou, M. and Burley, A. and Anderson, I. and Price, T. and Schofield, J. and MacLennan, G..Assessing the feasibility, acceptability and accessibility of a peer-delivered intervention to reduce harm and improve the well-being of people who experience homelessness with problem substance use: the SHARPS study.Cmaj.2022;21(1):10. | Population |
| Sokoloff, L. J. and Wu, J. and Eberly, L. A. and Nathan, A. S. and Julien, H. M. and Kobayashi, T. J. and Damrauer, S. M. and Groeneveld, P. W. and Tsai, J. and Khatana, S. A. M..Association of Homelessness and Unstable Housing With Cardiovascular Care Utilization Among Veterans.Journal of Clinical Oncology.2024;189(11):e010993. | No outcomes reported |
| Ma, C. T. and Gee, L. and Kushel, M. B..Associations between housing instability and food insecurity with health care access in low-income children.Prof Case Manag.2008;8(1):50-7. | Population |
| Friedman, M. S. and Marshal, M. P. and Stall, R. and Kidder, D. P. and Henny, K. D. and Courtenay-Quirk, C. and Wolitski, R. J. and Aidala, A. and Royal, S. and Holtgrave, D. R..Associations between substance use, sexual risk taking and HIV treatment adherence among homeless people living with HIV.J Prev (2022).2009;71(6):692-700. | Population |
| Lefeuvre, D. and Delmas, M. C. and Marguet, C. and Chauvin, P. and Vandentorren, S..Asthma-Like Symptoms in Homeless Children in the Greater Paris Area in 2013: Prevalence, Associated Factors and Utilization of Healthcare Services in the ENFAMS Survey.J Am Board Fam Med.2016;51(4):e0153872. | No outcomes reported |
| Buu, M. C. and Carter, L. and Bruce, J. S. and Baca, E. A. and Greenberg, B. and Chamberlain, L. J..Asthma, tobacco smoke and the indoor environment: A qualitative study of sheltered homeless families.Open Forum Infectious Diseases.2014;79(2):142-148. | Population |
| Cusack, M. and Graham, F. and Metraux, S. and Metzger, D. and Culhane, D..At the Intersection of Homeless Encampments and Heroin Addiction: Service Use Barriers, Facilitators, and Recommendations from the City of Philadelphia's Encampment Resolution Pilot.J Obstet Gynecol Neonatal Nurs.2021;52(2):150-163. | Population |
| Sánchez Hidalgo, M. and Andrés, M. S. and Canadell Rusiñol, J. and Fürstenheim Milerud, L. P. and Gómez Palomar, E. and Moya Tena, M..[Barriers and facilitators in accessing and using primary health care centers for people experiencing homelessness].Journal of Investigative Medicine.2024;25(9):102949. | Population |
| Armstrong, M. and Shulman, C. and Hudson, B. and Stone, P. and Hewett, N..Barriers and facilitators to accessing health and social care services for people living in homeless hostels: a qualitative study of the experiences of hostel staff and residents in UK hostels.Br J Gen Pract.2021;38 Suppl 2(10):e053185. | Intervention |
| Gallant, K. and Nijjar, P. and DeBeck, K. and Cui, M. and Kerr, T..Barriers and Facilitators to Accessing Opioid Agonist Therapy for Street-involved Adolescents and Young Adults in Vancouver.Womens Health Issues.2024;59():. | Population |
| Carmichael, C. and Schiffler, T. and Smith, L. and Moudatsou, M. and Tabaki, I. and Doñate-Martínez, A. and Alhambra-Borrás, T. and Kouvari, M. and Karnaki, P. and Gil-Salmeron, A. and Grabovac, I..Barriers and facilitators to health care access for people experiencing homelessness in four European countries: an exploratory qualitative study.Journal of Clinical Oncology.2023;183(1):206. | Population |
| Youn, S. J. and Sauer-Zavala, S. and Patrick, K. A. and Ahles, E. M. and Silvan, Y. A. and Greig, A. and Marques, L. and Shtasel, D. L..Barriers and Facilitators to Implementing a Short-Term Transdiagnostic Mental Health Treatment for Homeless Persons.J Community Health.2019;189(7):585-594. | Population |
| Kaplan, L. M. and Sudore, R. L. and Arellano Cuervo, I. and Bainto, D. and Olsen, P. and Kushel, M..Barriers and Solutions to Advance Care Planning among Homeless-Experienced Older Adults.Med Care.2020;107(10):1300-1306. | Population |
| Zolnikov, T. R. and Hammel, M. and Furio, F. and Eggleston, B..Barriers for homeless with dual diagnosis: lessons learned from intensive mobile psychosocial assertive community treatment program.J Health Care Poor Underserved.2021;91(4):169-182. | Population |
| Ungurean, A. and Malic, A. and Lesnic, E..Barriers in accessing the specialised healthcare providers in homeless patients with tuberculosis.Ricerca e Pratica.2020;60():. | Publication type |
| Hawa, A. C. and Barr, D. and Sheikholeslami, H..Barriers to and supports for tobacco and alcohol use disorder treatment among California's homeless.Journal of Pain and Symptom Management.2023;1034(2):129-140. | Population |
| Hwang, S. W. and Bugeja, A. L..Barriers to appropriate diabetes management among homeless people in Toronto.Bmj Open.2000;3(2):161-5. | Intervention |
| Hurwitz, H. M. and Anderson, N. S. and McCaffrey, K. and Combs, P. and Jackson, R. D. and Kilic, S. S. and Mariner, M. and Onger, T. and Sanders, K. and Stimpert, T. and Suwarna, J. and Weleff, J. and Shah, C. S..Barriers to cancer prevention among women experiencing homelessness who receive onsite mammography, patient navigation, and education (HOPE).BMC Public Health.2022;9(28):111. | Publication type |
| Parker, R. D. and Albrecht, H. A..Barriers to care and service needs among chronically homeless persons in a housing first program.Circulation: Cardiovascular Quality and Outcomes.2012;54(6):278-84. | Intervention |
| Meehan, A. A. and Jeffers, A. and Barker, J. and Ray, C. M. and Laws, R. L. and Fields, V. L. and Miedema, S. S. and Cha, S. and Cassell, C. H. and DiPietro, B. and Cary, M. and Yang, M. and McLendon, H. and Marcus, R. and Mosites, E..Barriers to COVID-19 Prevention Measures Among People Experiencing Homelessness with Substance Use Disorder or Serious Mental Illness.J Gen Intern Med.2023;13(6):663-678. | Population |
| MacKenzie, M. and Purkey, E..Barriers to End-of-Life Services for Persons Experiencing Homelessness as Perceived by Health and Social Service Providers.Addict Behav Rep.2019;15(6):847-857. | Population |
| Parke, D. M. and Kenney, R. M. and Bogojevich, J. and El-Khoury, C. and Joshi, S. and Brar, S. and MacDonald, L. and Salib, C. and MacDonald, N. and Veve, M. and Suleyman, G..Barriers to Improving Outcomes among People Experiencing Homelessness and People Who Inject Drugs Hospitalized for Complicated Infections.BMJ Paediatr Open.2023;102():S864-S865. | Publication type |
| Hunter, C. E. and Palepu, A. and Farrell, S. and Gogosis, E. and O'Brien, K. and Hwang, S. W..Barriers to Prescription Medication Adherence Among Homeless and Vulnerably Housed Adults in Three Canadian Cities.JAMA Netw Open.2015;10(3):154-61. | Population |
| Santiago, R. and Wisniewski, J..Barriers to retention in hepatitis c care continuum among homeless people of new Orleans.American Journal of Public Health.2020;70(2):499-500. | Publication type |
| Hirst, V. and Cuthill, F..Benefits of GP care in outreach settings for people experiencing homelessness: a qualitative study.Journal of Primary Care and Community Health.2021;55(709):e596-e603. | Population |
| Asgary, R. and Garland, V. and Sckell, B..Breast cancer screening among homeless women of New York City shelter-based clinics.Am J Public Health.2014;31(5):529-34. | No outcomes reported |
| Kilic, S. S. and Mayo, Z. S. and Weleff, J. and Strzalka, C. and Hall, E. F. and Obi, E. E. and Anderson, N. and Phelan, M. P. and Cherian, S. S. and Tendulkar, R. D. and Suh, J. H. and Shah, C. S..Breast cancer screening in persons experiencing homelessness.J Gen Intern Med.2022;61(16):. | Publication type |
| Festa, K. and Hirsch, A. E. and Cassidy, M. R. and Oshry, L. and Quinn, K. and Sullivan, M. M. and Ko, N. Y..Breast Cancer Treatment Delays at an Urban Safety Net Hospital Among Women Experiencing Homelessness.BMC Prim Care.2020;60(3):452-457. | No outcomes reported |
| Bradford, D. W. and Gaynes, B. N. and Kim, M. M. and Kaufman, J. S. and Weinberger, M..Can shelter-based interventions improve treatment engagement in homeless individuals with psychiatric and/or substance misuse disorders?: a randomized controlled trial.BMC Public Health.2005;7(8):763-8. | No outcomes reported |
| Long, H. L. and Tulsky, J. P. and Chambers, D. B. and Alpers, L. S. and Robertson, M. J. and Moss, A. R. and Chesney, M. A..Cancer screening in homeless women: attitudes and behaviors.Lancet Public Health.1998;62(3):276-92. | No outcomes reported |
| Zrouki, A. and Pacciani, A. and Ledonne, G..Care questions and answers. Adherence to therapeutic treatments for chronic illness in the homeless: A qualitative evaluation in the Milanese context.Vaccines (Basel).2017;8(5):198-214. | Population |
| Hutt, E. and Jones, J. and Albright, K. and Weber, M. and O'Toole, T..Challenges of providing end-of-life care for homeless veterans.J Hosp Palliat Nurs.2016;9(2):440-441. | Population |
| Hudson, B. F. and Shulman, C. and Low, J. and Hewett, N. and Daley, J. and Davis, S. and Brophy, N. and Howard, D. and Vivat, B. and Kennedy, P. and Stone, P..Challenges to discussing palliative care with people experiencing homelessness: a qualitative study.J Gerontol Soc Work.2017;42(11):. | Population |
| Pendyal, A. and Rosenthal, M. S. and Keene, D..Challenges to self-management of heart failure among homeless individuals: A qualitative study.Front Public Health.2019;101 Suppl 3():. | Publication type |
| Bourne, D. S. and Xue, L. and Hollander, M. A. G. and Cole, E. S. and Donohue, J. M..Changes in Medication Utilization and Adherence Associated with Homeless Adults' Entry into Permanent Supportive Housing.Med Care.2024;67(9):1590-1596. | Intervention |
| Macleod, S. L. and MacRae, P. and Pimenta, J..Children in street situations' access to healthcare: qualitative findings from the Street Child World Cup 2022.J Health Care Poor Underserved.2023;23(1):. | Population |
| Morris, M. D. and McDonell, C. and Luetkemeyer, A. F. and Thawley, R. and McKinney, J. and Price, J. C..Community-Based Point-of-Diagnosis Hepatitis C Treatment for Marginalized Populations: A Nonrandomized Controlled Trial.Soc Work Public Health.2023;12(10):e2338792. | Population |
| Rodriguez, N. M. and Balian, L. and Ziolkowski, R. and Case, X. and Smith, K. and Tipton, J..Community-informed interventions to address cervical cancer disparities among people experiencing homelessness.Med Care.2024;40(9):. | Publication type |
| Kertesz, S. G. and Holt, C. L. and Steward, J. L. and Jones, R. N. and Roth, D. L. and Stringfellow, E. and Gordon, A. J. and Kim, T. W. and Austin, E. L. and Henry, S. R. and Johnson, N. K. and Granstaff, U. S. and O'Connell, J. J. and Golden, J. F. and Young, A. S. and Davis, L. L. and Pollio, D. E..Comparing Homeless Persons' Care Experiences in Tailored Versus Nontailored Primary Care Programs.Journal of the American Association of Nurse Practitioners.2013;79():S331-S339. | No outcomes reported |
| Zeitler, M. and Williamson, A. E. and Budd, J. and Spencer, R. and Queen, A. and Lowrie, R..Comparing the Impact of Primary Care Practice Design in Two Inner City UK Homelessness Services.Vaccine.2020;2():. | No outcomes reported |
| Gelberg, L. and Gallagher, T. C. and Andersen, R. M. and Koegel, P..Competing priorities as a barrier to medical care among homeless adults in Los Angeles.Int J Drug Policy.1997;72(2):217-20. | No outcomes reported |
| Knight, K. R. and Duke, M. R. and Carey, C. A. and Pruss, G. and Garcia, C. M. and Lightfoot, M. and Imbert, E. and Kushel, M..COVID-19 Testing and Vaccine Acceptability Among Homeless-Experienced Adults: Qualitative Data from Two Samples.J Community Health.2022;24(4):823-829. | Population |
| Gin, J. L. and Balut, M. D. and Dobalian, A..COVID-19 vaccination uptake and receptivity among veterans enrolled in homelessness-tailored primary health care clinics: provider trust vs. misinformation.Nurs Res.2024;67(1):24. | Population |
| Balma, B. and Vasilakos, L. and Osman, I. and Elgonda, A. and Gewirtz O'Brien, J. R..COVID-19 vaccine attitudes among youth experiencing homelessness: a qualitative analysis with opportunities for action.Journal of Investigative Medicine.2023;62(1):1672. | Population |
| Richard, L. and Liu, M. and Jenkinson, J. I. R. and Nisenbaum, R. and Brown, M. and Pedersen, C. and Hwang, S. W..COVID-19 Vaccine Coverage and Sociodemographic, Behavioural and Housing Factors Associated with Vaccination among People Experiencing Homelessness in Toronto, Canada: A Cross-Sectional Study.PLoS One.2022;69(8):. | No outcomes reported |
| Stone, W. and Mixer, S. J. and Mendola, A..Culturally Acceptable Advance Care Planning and Advance Directives for Persons Experiencing Homelessness.Public Health Rep.2019;200(5):350-357. | Population |
| Weldrick, R. and Canham, S. L. and Sussman, T. and Walsh, C. A. and Cormier, É and Mahmood, A..Delivering Services to Older Persons Experiencing Homelessness: Providers' Perspectives of What Does and Does Not Work.Health Soc Care Community.2023;26(1):29-42. | Intervention |
| Desai, M. M. and Rosenheck, R. A. and Kasprow, W. J..Determinants of receipt of ambulatory medical care in a national sample of mentally ill homeless veterans.J Prim Care Community Health.2003;41(2):275-87. | No outcomes reported |
| Yaphe, H. M. and Campbell, R. B. and Mancini, N. L. and Grewal, E. K. and Tibebu, T. and Saunders-Smith, T. and Hwang, S. W. and Campbell, D. J. T..The Development of a Conceptual Framework for Providing Tailored Diabetes Care for Individuals Experiencing Homelessness: A Qualitative Study.Health Expect.2023;15(1):309-325. | Population |
| Kertesz, S. and Jones, R. and Roth, D. and Gordon, A. and Granstaff, U. and Holt, C. and Pollio, D. and Austin, E..Development of a new homeless primary care assessment survey using qualitative methods and item response theory.Subst Use Misuse.2013;1(2):520-521. | Publication type |
| Elder, N. C. and Tubb, M. R..Diabetes in homeless persons: barriers and enablers to health as perceived by patients, medical, and social service providers.BMC Public Health.2014;52(3):220-31. | Population |
| Jones, A. L. and Hausmann, L. R. M. and Kertesz, S. and Suo, Y. and Cashy, J. P. and Mor, M. K. and Schaefer, J. H., Jr. and Gundlapalli, A. V. and Gordon, A. J..Differences in Experiences With Care Between Homeless and Nonhomeless Patients in Veterans Affairs Facilities With Tailored and Nontailored Primary Care Teams.Int J Equity Health.2018;9(7):610-618. | No outcomes reported |
| Parpouchi, M. and Moniruzzaman, A. and Rezansoff, S. N. and Russolillo, A. and Somers, J. M..The effect of Housing First on adherence to methadone maintenance treatment.J Transcult Nurs.2018;61():73-80. | Intervention |
| Wainwright, M. K. and Earle, M. and Kosog, K. and Gilbert, M. K. and Nolan, C. and Stellon, E. and Vitolo, O. and Canar, W. J..The Effect of Place of Service on Diabetic Screening Adherence in the Homeless Population.J Emerg Nurs.2020;13(1):73-80. | No outcomes reported |
| Nyamathi, A. and Nahid, P. and Berg, J. and Burrage, J. and Christiani, A. and Aqtash, S. and Morisky, D. and Leake, B..Efficacy of nurse case-managed intervention for latent tuberculosis among homeless subsamples.BMC Health Serv Res.2008;16(1):33-9. | No outcomes reported |
| Hoover, D. and Glazer, P. and Betat, D. and Wu, P. and Holloway, R. and Howe, B. and Debay, M. and Neeki, M..Emergency services utilization of adult homeless patients in a large public hospital in San Bernardino County, California.Children and Youth Services Review.2015;96(1):166. | Publication type |
| Shulman, C. and Hudson, B. F. and Low, J. and Hewett, N. and Daley, J. and Kennedy, P. and Davis, S. and Brophy, N. and Howard, D. and Vivat, B. and Stone, P..End-of-life care for homeless people: A qualitative analysis exploring the challenges to access and provision of palliative care.Health SA.2018;11(1):36-45. | Population |
| Jain, N. and Adams, E. A. and Joyes, E. C. and McLellan, G. and Burrows, M. and Paisi, M. and McGowan, L. J. and Iafrate, L. and Landes, D. and Watt, R. and Sniehotta, F. F. and Kaner, E. and Ramsay, S. E..Engagement and access to support for oral health, substance use, smoking and diet by people with severe and multiple disadvantage: A qualitative study.BMC Health Serv Res.2024;6(12):e0315254. | Population |
| Noska, A. J. and Belperio, P. S. and Loomis, T. P. and O'Toole, T. P. and Backus, L. I..Engagement in the Hepatitis C Care Cascade Among Homeless Veterans, 2015.Journal of Midwifery & Womens Health.2017;66(2):136-139. | No outcomes reported |
| Nyamathi, A. M. and Wall, S. A. and Yadav, K. and Shin, S. S. and Chang, A. and Arce, N. and Cuellar, H. and Fernando, S. and White, K. and Gelberg, L. and Salem, B. E..Engaging the Community in Designing a Hepatitis C Virus Treatment Program for Adults Experiencing Homelessness.Sex Res Social Policy.2021;187(11):2069-2083. | Population |
| Oosman, S. and Weber, G. and Ogunson, M. and Bath, B..Enhancing Access to Physical Therapy Services for People Experiencing Poverty and Homelessness: The Lighthouse Pilot Project.Journal of Occupational Science.2019;189(2):176-186. | Population |
| Clark, E. and Player, E. and Gillam, T. and Hanson, S. and Steel, N..Evaluating a specialist primary care service for patients experiencing homelessness: a qualitative study.Psychol Serv.2020;193(3):. | Population |
| Paisi, M. and Baines, R. and Worle, C. and Withers, L. and Witton, R..Evaluation of a community dental clinic providing care to people experiencing homelessness: A mixed methods approach.Substance Abuse.2020;196(5):1289-1299. | Population |
| De Los Reyes, G. and Ng, A. and Valencia Chavez, J. and Apollonio, D. E. and Kroon, L. and Lee, P. and Vijayaraghavan, M..Evaluation of a Pharmacist-Linked Smoking Cessation Intervention for Adults Experiencing Homelessness.Nurse Pract.2023;200(12):1519-1527. | Population |
| Abramovich, A. and Pang, N. and Kunasekaran, S. and Moss, A. and Kiran, T. and Pinto, A. D..Examining COVID-19 vaccine uptake and attitudes among 2SLGBTQ+ youth experiencing homelessness.BMC Health Serv Res.2022;9(1):122. | Population |
| Anastasiya, L. and Melanie, W. and Bartels, A. S. and Judy, F. and Eva, P..Examining the experiences of vulnerably housed patients visiting Kingston, Ontario's emergency departments: a qualitative analysis.Journal of General Internal Medicine.2024;51(1):139. | Population |
| Purkey, E. and MacKenzie, M..Experience of healthcare among the homeless and vulnerably housed a qualitative study: opportunities for equity-oriented health care.AIDS Care.2019;13(1):101. | Population |
| Chrystal, J. G. and Glover, D. L. and Young, A. S. and Whelan, F. and Austin, E. L. and Johnson, N. K. and Pollio, D. E. and Holt, C. L. and Stringfellow, E. and Gordon, A. J. and Kim, T. A. and Daigle, S. G. and Steward, J. L. and Kertesz, S. G..Experience of primary care among homeless individuals with mental health conditions.Pilot Feasibility Stud.2015;26(2):e0117395. | No outcomes reported |
| Gebreyesus, H. and Mamo, A. and Teweldemedhin, M. and Gidey, B. and Hdush, Z. and Birhanu, Z..Experiences of homeless women on maternity health service utilization and associated challenge in Aksum town, Northern Ethiopia.Health Promot J Austr.2019;14(1):359. | Population |
| Lonnie, E. and Pooja, S. and Allison, G. and Reuben, K. and David, A. and Juddy, W. and Edith, A. and Paula, B..Exploring patient-provider interactions and the health system's responsiveness to street-connected children and youth in Kenya: a qualitative study.J Acad Ophthalmol (2017).2021;20(1):363. | Population |
| Galvin, A. M. and Bergh, R. E. and Walters, S. T. and Lewis, M. A. and Thompson, E. L..Exploring Postpartum Pregnancy Prevention Behaviors Among Women Experiencing Homelessness: A Mixed-Methods Analysis.Scand J Caring Sci.2024;55():. | Population |
| Varley, A. L. and Montgomery, A. E. and Steward, J. and Stringfellow, E. and Austin, E. L. and Gordon, A. J. and Pollio, D. and deRussy, A. and Hoge, A. and Gelberg, L. and Riggs, K. and Kim, T. W. and Rubens, S. L. and Kertesz, S. G..Exploring Quality of Primary Care for Patients Who Experience Homelessness and the Clinicians Who Serve Them: What Are Their Aspirations?.Open Forum Infect Dis.2020;15(6):865-879. | Population |
| Boden, L. and Wolski, A. and Rubin, A. S. and Oliveira, L. P. and Tyminski, Q. P..Exploring the barriers and facilitators to menstrual hygiene management for women experiencing homelessness.Child Youth Serv Rev.2023;29(2):235-250. | Population |
| Coles, E. and Freeman, R..Exploring the oral health experiences of homeless people: a deconstruction-reconstruction formulation.AIDS Patient Care STDS.2016;330(1):53-63. | Population |
| Collins, S. E. and Duncan, M. H. and Smart, B. F. and Saxon, A. J. and Malone, D. K. and Jackson, T. R. and Ries, R. K..Extended-Release Naltrexone and Harm Reduction Counseling for Chronically Homeless People With Alcohol Dependence.JMIR Mhealth Uhealth.2015;107(1):21-33. | Population |
| Kacheyo, S. M. and Nyirenda, L..Factors associated with access and utilization of sexual abuse emergency healthcare services among street children in Zomba, Malawi: a qualitative study.Soc Sci Med.2024;1(1):1410. | Population |
| Fields, J. D. and Nguyen, K. H. and Moore, T. and Birkmeyer, J. and Perry, E. and Kushel, M..FACTORS ASSOCIATED WITH ACUTE HEALTHCARE USE IN A REPRESENTATIVE SAMPLE OF ADULTS EXPERIENCING HOMELESSNESS IN CALIFORNIA.J Urban Health.2023;23():S180. | Publication type |
| Royal, S. W. and Kidder, D. P. and Patrabansh, S. and Wolitski, R. J. and Holtgrave, D. R. and Aidala, A. and Pals, S. and Stall, R..Factors associated with adherence to highly active antiretroviral therapy in homeless or unstably housed adults living with HIV.Development Southern Africa.2009;33(4):448-55. | Population |
| Edgar, N. E. and Bennett, A. and Dunn, N. S. and MacLean, S. E. and Hatcher, S..Feasibility and acceptability of Narrative Exposure Therapy to treat individuals with PTSD who are homeless or vulnerably housed: a pilot randomized controlled trial.Journal of General Internal Medicine.2022;32(1):83. | Population |
| Osilla, K. C. and Kennedy, D. P. and Hunter, S. B. and Maksabedian, E..Feasibility of a computer-assisted social network motivational interviewing intervention for substance use and HIV risk behaviors for housing first residents.Qual Health Res.2016;99(1):14. | Population |
| Bower, M. and Olsen, N. and Peach, N. and Green, O. and Duarte, C. and Valpiani, P. and Teesson, M..Feasibility of telehealth counselling pilot for people experiencing homelessness and/or complex needs: During COVID-19 and beyond.Cjem.2023;6(4):889-894. | Population |
| Hennein, L. and Spaulding, K. A. and Karlegan, V. and Nnamani Silva, O. N. and de Alba Campomanes, A. G..Follow-Up Rates at a Free Ophthalmology Clinic at a Homeless Shelter.J Adv Nurs.2021;77(1):e51-e56. | No outcomes reported |
| Mokashi, V. and Gilchrist, J. and Smieja, N. and Maciejewski, J. and Marttala, S. and Beal, K. and Mbuagbaw, L. and Bulir, D. and Smieja, M. and O'Shea, T..Four COVID-19 screening strategies for early case identification within the homeless shelter population: a cluster randomized controlled trial.Public Health.2024;99(1):1478. | No outcomes reported |
| Murphy, K. and Embleton, L. and Lachman, J. M. and Owino, E. and Kirwa, S. and Makori, D. and Braitstein, P.."From Analog to Digital": The Feasibility, Acceptability, and Preliminary Outcomes of a Positive Parenting Program for Street-Connected Mothers in Kenya.J Community Health.2021;2020():. | Population |
| Rajabiun, S. and Mallinson, R. K. and McCoy, K. and Coleman, S. and Drainoni, M. L. and Rebholz, C. and Holbert, T.."Getting me back on track": the role of outreach interventions in engaging and retaining people living with HIV/AIDS in medical care.J Am Acad Nurse Pract.2007;22():S20-9. | Population |
| Neale, J. and Kennedy, C..Good practice towards homeless drug users: research evidence from Scotland.Int J Equity Health.2002;38(3):196-205. | Intervention |
| Brenchley, A..Graphic Paper: The perspectives of homeless people using the services of a mobile health clinic in relation to their health needs: a qualitative study on community-based outreach nursing.J Viral Hepat.2024;71(8):615-621. | Publication type |
| Robbins, J. L. and Wenger, L. and Lorvick, J. and Shiboski, C. and Kral, A. H..Health and oral health care needs and health care-seeking behavior among homeless injection drug users in San Francisco.Respir Care.2010;13(6):920-30. | No outcomes reported |
| Seager, J. R. and Tamasane, T..Health and well-being of the homeless in South African cities and towns.J Assoc Nurses AIDS Care.2010;18(1):63-83. | Intervention |
| Kreider, B. and Nicholson, S..Health insurance and the homeless.International Journal of Mental Health and Addiction.1997;39(1):31-41. | No outcomes reported |
| Taplin, J. G. and Barnabe, C. M. and Blanchard, I. E. and Doig, C. J. and Crowshoe, L. and Clement, F. M..Health service utilization by people experiencing homelessness and engaging with community paramedics: a pre-post study.J Public Health (Oxf).2022;26(8):885-889. | No outcomes reported |
| Kidder, D. P. and Wolitski, R. J. and Campsmith, M. L. and Nakamura, G. V..Health status, health care use, medication use, and medication adherence among homeless and housed people living with HIV/AIDS.Rev Bras Enferm.2007;12(12):2238-45. | No outcomes reported |
| Ungpakorn, R. and Rae, B..Health-related street outreach: Exploring the perceptions of homeless people with experience of sleeping rough.Subst Abus.2020;61(1):253-263. | Intervention |
| Vijayaraghavan, M. and Tochterman, A. and Hsu, E. and Johnson, K. and Marcus, S. and Caton, C. L..Health, access to health care, and health care use among homeless women with a history of intimate partner violence.Soc Psychiatry Psychiatr Epidemiol.2012;196(5):1032-9. | No outcomes reported |
| Chelvakumar, G. and Ford, N. and Kapa, H. M. and Lange, H. L. H. and McRee, A. L. and Bonny, A. E..Healthcare Barriers and Utilization Among Adolescents and Young Adults Accessing Services for Homeless and Runaway Youth.American Journal of Drug and Alcohol Abuse.2017;72(3):437-443. | Population |
| Cernadas, A. and Fernández, Á.Healthcare inequities and barriers to access for homeless individuals: a qualitative study in Barcelona (Spain).J Urban Health.2021;47(1):84. | Population |
| Bajis, S. and Grebely, J. and Cooper, L. and Smith, J. and Owen, G. and Chudleigh, A. and Hajarizadeh, B. and Martinello, M. and Adey, S. and Read, P. and Gilliver, R. and Applegate, T. and Treloar, C. and Maher, L. and Dore, G. J..Hepatitis C virus testing, liver disease assessment and direct-acting antiviral treatment uptake and outcomes in a service for people who are homeless in Sydney, Australia: The LiveRLife homelessness study.J Gen Intern Med.2019;(8):969-979. | Population |
| Phillips, M. and Richardson, L. and Wood, E. and Nguyen, P. and Kerr, T. and DeBeck, K..High-Intensity Drug Use and Health Service Access Among Street-Involved Youth in a Canadian Setting.Am J Public Health.2015;122(14):1805-13. | Population |
| Fogg, C. J. and Mawn, B..HIV screening: beliefs and intentions of the homeless.Schizophr Bull.2010;12(5):395-407. | No outcomes reported |
| Hawk, M. and Maulsby, C. and Enobun, B. and Kinsky, S..HIV Treatment Cascade by Housing Status at Enrollment: Results from a Retention in Care Cohort.Int J Environ Res Public Health.2019;93(3):765-775. | Population |
| Montgomery, B. E. E. and Crone, C. and Goodwin, B. and Hokans, R. and Williams, A. and Stacker, J. and Borne, R. and Pro, G. and Martel, I..Home Together: A Multi-Level Community-Based Health Promotion Program Supporting Families Experiencing Homelessness.Health Care Anal.2024;13(3):880-902. | Population |
| Burra, T. A. and Hwang, S. W. and Rourke, S. B. and Stergiopoulos, V..Homeless and Housed Inpatients with Schizophrenia: Disparities in Service Access upon Discharge from Hospital.Sex Transm Dis.2012;3(5):778-789. | Population |
| Hatton, D. C. and Kleffel, D. and Bennett, S. and Gaffrey, E. A..Homeless women and children's access to health care: a paradox.Behav Ther.2001;4(1):25-34. | Population |
| Tinland, A. and Zemmour, K. and Auquier, P. and Boucekine, M. and Girard, V. and Loubière, S. and Fond, G. and Boyer, L..Homeless women with schizophrenia reported lower adherence to their medication than men: results from the French Housing First experience.Qualitative Health Research.2017;115(9):1113-1122. | Population |
| Upshur, C. C. and Jenkins, D. and Weinreb, L. and Gelberg, L. and Orvek, E. A..Homeless women's service use, barriers, and motivation for participating in substance use treatment.International Journal of Qualitative Studies on Health and Well-Being.2018;22(2):252-262. | Population |
| Black, E. B. and Fedyszyn, I. E. and Mildred, H. and Perkin, R. and Lough, R. and Brann, P. and Ritter, C..Homeless youth: Barriers and facilitators for service referrals.Front Public Health.2018;14():7-12. | Population |
| Palepu, A. and Milloy, M. J. and Kerr, T. and Zhang, R. and Wood, E..Homelessness and adherence to antiretroviral therapy among a cohort of HIV-infected injection drug users.J Prim Care Community Health.2011;190(3):545-55. | No outcomes reported |
| Manser, S. T. and Sekar, P. and Bonilla, Z. and Ford, B. and Shippee, N. and Busch, A. M. and Gelberg, L. and Rogers, E. A. and Jennings-Dedina, L. and Montori, V. M. and Vickery, K. D..Homelessness and Type 2 Diabetes: A Qualitative Study of Facilitators and Barriers to Self-Management and Medication Adherence.Gend Med.2024;61(16):3120-3128. | Intervention |
| Schanzer, B. and Dominguez, B. and Shrout, P. E. and Caton, C. L..Homelessness, health status, and health care use.J Sch Nurs.2007;193(3):464-9. | No outcomes reported |
| Winn, J. L. and Shealy, S. E. and Kropp, G. J. and Felkins-Dohm, D. and Gonzales-Nolas, C. and Francis, E..Housing assistance and case management: improving access to substance use disorder treatment for homeless veterans.Anthrozoos.2013;119(2):233-40. | Population |
| Rezansoff, S. N. and Moniruzzaman, A. and Fazel, S. and McCandless, L. and Procyshyn, R. and Somers, J. M..Housing First Improves Adherence to Antipsychotic Medication Among Formerly Homeless Adults With Schizophrenia: Results of a Randomized Controlled Trial.Int J Environ Res Public Health.2017;32(4):852-861. | Population |
| Heaslip, V. and Green, S. and Simkhada, B. and Dogan, H. and Richer, S..How Do People Who Are Homeless Find Out about Local Health and Social Care Services: A Mixed Method Study.Arch Gen Psychiatry.2021;6(1):. | Intervention |
| Hughes, N. R..How Does Organisational Literacy Impact Access to Health Care for Homeless Individuals?.J Ment Health.2017;22(1):90-106. | No outcomes reported |
| Cironi, K. A. and Jones, A. T. and Hauser, E. M. and Olsen, J. W. and Kissinger, P. J..Human Immunodeficiency Virus and Hepatitis C Linkage-to-Care Initiative for New Orleans Residents Experiencing Homelessness During the COVID-19 Pandemic.Journal of Social Distress and the Homeless.2021;44(8):595-600. | No outcomes reported |
| Zahir, A. and Yip, D. and Garcia, C. and Smith, A. N. and Dhatt, Z. and Duke, M. and Kushel, M.."I Needed for You to See What I'm Talking About": Experiences With Telehealth Among Homeless-Experienced Older Adults.BMC Public Health.2023;44 Suppl 1():23337214231172650. | Population |
| Rew, L. and Yeargain, O. and Peretz, C. and Croce, E.."I'm losing everything all over again": Responses from youth experiencing homelessness during the COVID-19 pandemic.J Am Pharm Assoc (2003).2021;32(6):653-657. | Intervention |
| Fine, D. R. and Critchley, N. and Hart, K. and Joyce, A. and Sporn, N. and Gaeta, J. and Wright, J. and Baggett, T. P. and Kruse, G.."I'm on the Right Path": Exploring 1-Month Retention in a Homeless-Tailored Outpatient-Based Opioid Treatment Program.Am J Public Health.2024;25(2):268-277. | Population |
| Wu, Q. and Zhang, J. and Walsh, L. and Slesnick, N..Illicit Drug Use, Cognitive Distortions, and Suicidal Ideation Among Homeless Youth: Results From a Randomized Controlled Trial.AIDS Educ Prev.2022;11(1):92-104. | No outcomes reported |
| Ensign, J. and Bell, M..Illness experiences of homeless youth.Am J Public Health.2004;124(9):1239-1254. | Intervention |
| Ali, M. and de Muynck, A..Illness incidence and health seeking behaviour among street children in Rawalpindi and Islamabad, Pakistan - a qualitative study.Br J Community Nurs.2005;35(5):525-32. | Population |
| Håkanson, C. and Öhlén, J..Illness narratives of people who are homeless.BMJ Open.2016;18():. | Intervention |
| Graf, W. and Bertram, F. and Dost, K. and Brennecke, A. and Kowalski, V. and van Rüth, V. and Nörz, D. S. and Wulff, B. and Ondruschka, B. and Püschel, K. and Pfefferle, S. and Lütgehetmann, M. and Heinrich, F..Immunity against measles, mumps, rubella, and varicella among homeless individuals in Germany - A nationwide multi-center cross-sectional study.Subst Use Misuse.2024;4():1375151. | Intervention |
| Tominc, B. L. and Francis, K. L. and Sawyer, S. M. and Heerde, J. A. and O'Neill, J. and Henning, D..Immunization Coverage in Young People Experiencing Homelessness and the Impact of a Nurse-led Program.Am J Hosp Palliat Care.2023;8():21501319231204581. | Population |
| Cheng, A. L. and Kelly, P. J..Impact of an integrated service system on client outcomes by gender in a national sample of a mentally ill homeless population.J Technol Behav Sci.2008;72(4):395-404. | No outcomes reported |
| Morris, R. I. and Strong, L..The impact of homelessness on the health of families.Br J Gen Pract.2004;19(4):221-7. | Intervention |
| Ramirez, V. and Frisbie, L. and Robinson, J. and Rabinowitz, P. M..The Impact of Pet Ownership on Healthcare-Seeking Behavior in Individuals Experiencing Homelessness.J Gen Intern Med.2022;178(5):615-632. | Population |
| Dawes, J. and May, T. and Fancourt, D. and Burton, A..The Impact of the COVID-19 Pandemic and Associated Societal Restrictions on People Experiencing Homelessness (PEH): A Qualitative Interview Study with PEH and Service Providers in the UK..2022;43(23):. | Population |
| Kashner, T. M. and Rosenheck, R. and Campinell, A. B. and Surís, A. and Crandall, R. and Garfield, N. J. and Lapuc, P. and Pyrcz, K. and Soyka, T. and Wicker, A..Impact of work therapy on health status among homeless, substance-dependent veterans: a randomized controlled trial.Open Heart.2002;60(10):938-44. | Intervention |
| Khan, Z. and McCrone, P. and Koehne, S..Impact on the use and cost of other services following intervention by an inpatient pathway homelessness team in an acute mental health hospital.Laryngoscope.2022;56(3):325-331. | No outcomes reported |
| Lee, K. and Murphy, E. R. and Cassidy, J. and Chen, Z. R. and Rhee, T. G..Impacts of health insurance on older adults with histories of homelessness.Drug Alcohol Rev.2022;82(1):1-10. | No outcomes reported |
| Pratt, R. and Xiong, S. and Kmiecik, A. and Strobel-Ayres, C. and Joseph, A. and Rose, S. A. E. and Luo, X. and Cooney, N. and Thomas, J. and Specker, S. and Okuyemi, K..The implementation of a smoking cessation and alcohol abstinence intervention for people experiencing homelessness.Journal of Addiction Medicine.2022;10(1):1260. | Population |
| Albertson, S. and Murray, T. and Triboletti, J. and Pence, L. and Gonzalvo, J. and Meredith, A. and Walroth, T. and Rodgers, J. and Crane, L. and Sidle, J..Implementation of primary care clinical pharmacy services for adults experiencing homelessness.J Gen Intern Med.2021;99(1):e80-e84. | Population |
| Anaya, H. D. and Butler, J. N. and Knapp, H. and Chan, K. and Conners, E. E. and Rumanes, S. F..Implementing an HIV Rapid Testing-Linkage-to-Care Project Among Homeless Individuals in Los Angeles County: A Collaborative Effort Between Federal, County, and City Government.BMC Public Health.2015;16(1):85-90. | Population |
| Neumiller, S. and Bennett-Clark, F. and Young, M. S. and Dates, B. and Broner, N. and Leddy, J. and Kendall, D. and Richards, S. and De Jong, F..Implementing Assertive Community Treatment in Diverse Settings for People Who Are Homeless with Co-Occurring Mental and Addictive Disorders: A Series of Case Studies.Health Psychol.2009;40(3):239-263. | Population |
| Rosenheck, R. and Frisman, L. and Kasprow, W..Improving access to disability benefits among homeless persons with mental illness: an agency-specific approach to services integration.Nurs Forum.1999;39(4):524-8. | Intervention |
| McGregor, F. and Paisi, M. and Robinson, A. and Shawe, J..Improving the sexual health of young people experiencing homelessness.Journal of Epidemiology and Community Health.2021;22(11):560-566. | Population |
| Ziolkowski, R. A. and Balian, L. and Sridhar, S. and Rodriguez, N. M..Improving uptake of COVID-19 testing and vaccination in a homeless population: mixed-methods evaluation of community health worker-led education in a shelter.J Subst Abuse Treat.2024;191(12):e087134. | Population |
| Phillips, M. and DeBeck, K. and Desjarlais, T. and Morrison, T. and Feng, C. and Kerr, T. and Wood, E..Inability to access addiction treatment among street-involved youth in a Canadian setting.Int J Ment Health Nurs.2014;118(10):1233-40. | Population |
| Krakowsky, Y. and Gofine, M. and Brown, P. and Danziger, J. and Knowles, H..Increasing access--a qualitative study of homelessness and palliative care in a major urban center.Disabil Health J.2013;105(3):268-70. | Population |
| Conti, J. and Dryden, E. and Fincke, B. G. and Dunlap, S. and McInnes, D. K..Innovative Approaches to Engaging Homeless and Marginally Housed Patients in Care: a Case Study of Hepatitis C.Addict Sci Clin Pract.2023;74(1):156-164. | Population |
| Bark, P. and Ramasawmy, M. and Hayward, A. and Luchenski, S. A. and Aldridge, R. and Burridge, S. and Banerjee, A..AN INTEGRATED APPROACH TO CARDIOVASCULAR DISEASE IN HOMELESS INDIVIDUALS: A QUALITATIVE STUDY.J Addict Med.2022;72():. | Intervention |
| Bark, P. and Ramasawmy, M. and Hayward, A. and Luchenski, S. and Aldridge, R. and Burridge, S. and Banerjee, A..Integrated approach to cardiovascular disease in people experiencing homelessness: a qualitative study.Kidney Int Suppl (2011).2023;103(1):. | Intervention |
| Carver, H. and Parkes, T. and Browne, T. and Matheson, C. and Pauly, B..Investigating the need for alcohol harm reduction and managed alcohol programs for people experiencing homelessness and alcohol use disorders in Scotland.Holist Nurs Pract.2021;73(2):220-230. | Population |
| Moeller, D. and Nowinski-Konchak, J..Is Homelessness or Housing Insecurity a Barrier to Effective Addiction Treatment?.Arch Gen Psychiatry.2020;21(6):e399-e400. | Publication type |
| McInnes, D. K. and Troszak, L. K. and Fincke, B. G. and Shwartz, M. and Midboe, A. M. and Gifford, A. L. and Dunlap, S. and Byrne, T..Is the Availability of Direct-Acting Antivirals Associated with Increased Access to Hepatitis C Treatment for Homeless and Unstably Housed Veterans?.Journal of Progressive Human Services.2022;188(5):1038-1044. | Population |
| Tam, C. and Wesseling, T. and Wang, L. and Salters, K. and Moore, D. M. and Dawydiuk, N. and Zhu, J. and Grieve, S. and Bingham, B. and McLinden, T. and Hogg, R. and Barrios, R..It's all about connection: Determinants of social support and the influence on HIV treatment interruptions among people living with HIV in British Columbia, Canada.Cad Saude Publica.2023;187(1):2524. | Population |
| Nyamathi, A. and Stein, J. A. and Schumann, A. and Tyler, D..Latent variable assessment of outcomes in a nurse-managed intervention to increase latent tuberculosis treatment completion in homeless adults.Inquiry.2007;61(1):68-76. | Population |
| Santa Maria, D. and Paul, M. and Lightfoot, M. and Nyamathi, A. and Quadri, Y. and Padhye, N. and Ocampo, E. and Babayev, R. and Ramos, M. W. and Malone-Thomas, S. L. and White, M. and Carrillo, A. and Jones, J. T..The Leaky Preexposure Prophylaxis Cascade: Barriers and Facilitators to Preexposure Prophylaxis Uptake and Adherence Among Youth Experiencing Homelessness in the US South, a Mixed Methods Study.J Community Health Nurs.2024;20():. | Population |
| Thirkle, S. A. and Adams, E. A. and Harland, J. and Kaner, E. and Ramsay, S. E..'THE LIGHTS ARE ON, AND THE DOORS ARE ALWAYS OPEN': A QUALITATIVE STUDY TO UNDERSTAND CHALLENGES UNDERLYING THE NEED FOR EMERGENCY CARE IN PEOPLE EXPERIENCING HOMELESSNESS IN RURAL AND COASTAL NORTH-EAST ENGLAND.J Am Acad Nurse Pract.2023;76():A4. | Population |
| Matheson, C. and Foster, R. and Schofield, J. and Browne, T..Long-acting depot buprenorphine in people who are homeless: Views and experiences.Res Social Adm Pharm.2022;40():108781. | No outcomes reported |
| Burns, A. and Robins, A. and Hodge, M. and Holmes, A..Long-term homelessness in men with a psychosis: Limitation of services.Int J Pharm Pract.2009;52(2):126-32. | Intervention |
| Glendening, Z. S. and McCauley, E. and Shinn, M. and Brown, S. R..Long-term housing subsidies and SSI/SSDI income: Creating health-promoting contexts for families experiencing housing instability with disabilities.Am J Hosp Palliat Care.2018;16(2):214-220. | Intervention |
| Lee, C. T. and Winquist, A. and Wiewel, E. W. and Braunstein, S. and Jordan, H. T. and Gould, L. H. and Gwynn, R. C. and Lim, S..Long-Term Supportive Housing is Associated with Decreased Risk for New HIV Diagnoses Among a Large Cohort of Homeless Persons in New York City.BMC Public Health.2018;47(9):3083-3090. | No outcomes reported |
| Shearer, R. D. and Shippee, N. D. and Vickery, K. D. and Stevens, M. A. and Winkelman, T. N. A..A longitudinal cross-sectional analysis of substance use treatment trends for individuals experiencing homelessness, criminal justice involvement, both, or neither - United States, 2006-2018.Journal of Social Distress and the Homeless.2022;11():. | Population |
| O'Donnell, A. and Cabral, H. J. and Kertesz, S. G. and Rajabiun, S..Longitudinal experience of primary care in a cohort of multiply diagnosed persons with HIV experiencing homelessness.Journal of Social Distress and the Homeless.2024;74(1):152-163. | No outcomes reported |
| Carter, J. and Zevin, B. and Lum, P. J..Low barrier buprenorphine treatment for persons experiencing homelessness and injecting heroin in San Francisco.Int J Environ Res Public Health.2019;14(1):20. | No outcomes reported |
| Jones, A. L. and Gelberg, L. and deRussy, A. J. and Varley, A. L. and Riggs, K. R. and Gordon, A. J. and Kertesz, S. G..Low Uptake of Secure Messaging Among Veterans With Experiences of Homelessness and Substance Use Disorders.J Appl Lab Med.2021;15(6):508-511. | No outcomes reported |
| Podymow, T. and Turnbull, J..Management of chronic kidney disease and dialysis in homeless persons.Journal of Social Distress and the Homeless.2013;53(2):230-235. | No outcomes reported |
| Paisi, M. and Witton, R. and Burrows, M. and Allen, Z. and Plessas, A. and Withers, L. and McDonald, L. and Kay, E..Management of plaque in people experiencing homelessness using 'peer education': a pilot study.Am J Addict.2019;14(11):860-866. | No outcomes reported |
| Weinrich, S. P. and Bormann, J. E. and Glaser, D. and Hardin, S. and Barger, M. and Lizarraga, C. and Del Rio, J. and Allard, C. B..Mantram Repetition With Homeless Women: A Pilot Study.BMC Infect Dis.2016;18(6):360-367. | No outcomes reported |
| Tsai, A. C. and Weiser, S. D. and Petersen, M. L. and Ragland, K. and Kushel, M. B. and Bangsberg, D. R..A marginal structural model to estimate the causal effect of antidepressant medication treatment on viral suppression among homeless and marginally housed persons with HIV.BJGP Open.2010;4(12):1282-90. | Population |
| Johnson, I. M. and Light, M. A..Meaningful Healthcare and Social Service Access for Homeless Populations: Generating Alliances Through Theories of Therapeutic Landscape.Psychol Serv.2024;21(1):120-141. | Intervention |
| Aguiar, M. M. and Iriart, J. A..[Meanings and practices associated with health and illness among the homeless in Salvador, Bahia State, Brazil].J Health Care Poor Underserved.2012;24(1):115-24. | No outcomes reported |
| Glied, S. and Hoven, C. and Moore, R. E. and Garrett, A. B..Medicaid and service use among homeless adults.Rev Bras Enferm.1998;106(4):380-8. | No outcomes reported |
| Zur, J. and Linton, S. and Mead, H..Medical Respite and Linkages to Outpatient Health Care Providers among Individuals Experiencing Homelessness.Journal of Adolescent Health.2016;67(2):81-9. | Population |
| Venkat, D. and Perri, P. and Cuevas, E. and Richards, H. and Ling, B. and Dipre, D. L. and Park, P. and Deutsch, K. and Watson, C. and Fisk, S..Medical respite: Improving healthcare utilization in vulnerable populations.J Med Internet Res.2018;80(2):819. | Publication type |
| Burda, C. and Haack, M. and Duarte, A. C. and Alemi, F..Medication adherence among homeless patients: a pilot study of cell phone effectiveness.Am J Public Health.2012;42(11):675-81. | Population |
| Watson, T. and Schindel, T. J. and Simpson, S. H. and Hughes, C. A..Medication adherence in patients with mental illness and recent homelessness: contributing factors and perceptions on mobile technology use.JAMA Netw Open.2020;18(4):362-369. | Population |
| Gruenewald, D. A. and Doan, D. and Poppe, A. and Jones, J. and Hutt, E.."Meet Me Where I Am": Removing Barriers to End-of-Life Care for Homeless Veterans and Veterans Without Stable Housing.Can J Ophthalmol.2018;19(12):1483-1489. | Population |
| DeMaria, A. L. and Martinez, R. and Otten, E. and Schnolis, E. and Hrubiak, S. and Frank, J. and Cromer, R. and Ruiz, Y. and Rodriguez, N. M..Menstruating while homeless: navigating access to products, spaces, and services.Journal of Womens Health.2024;28(1):909. | Population |
| Dauriac-Le Masson, V. and Mercuel, A. and Guedj, M. J. and Douay, C. and Chauvin, P. and Laporte, A..Mental Healthcare Utilization among Homeless People in the Greater Paris Area.BMC Health Serv Res.2020;73(21):. | No outcomes reported |
| Kinczewski, A. E. and Johnson, E. E. and Szymkowiak, D. and Pfirrman, S. J. and O'Toole, T. P..Mixed methods analysis of a national implementation of a medical respite program in transitional housing settings for veterans experiencing homelessness.J Gen Intern Med.2023;69(1):97-103. | Population |
| Iheanacho, T. and Payne, K. and Tsai, J..Mobile, Community-Based Buprenorphine Treatment for Veterans Experiencing Homelessness With Opioid Use Disorder: A Pilot, Feasibility Study.J Public Health Dent.2020;20(6):485-491. | No outcomes reported |
| Queen, A. B. and Lowrie, R. and Richardson, J. and Williamson, A. E..Multimorbidity, disadvantage, and patient engagement within a specialist homeless health service in the UK: an in-depth study of general practice data.Drug and Alcohol Dependence.2017;17(3):bjgpopen17X100941. | No outcomes reported |
| Jones, A. L. and Hausmann, L. R. M. and Haas, G. L. and Mor, M. K. and Cashy, J. P. and Schaefer, J. H. and Gordon, A. J..A national evaluation of homeless and nonhomeless veterans' experiences with primary care.Medicina (Brazil).2017;10(2):174-183. | Population |
| O'Toole, T. P. and Johnson, E. E. and Redihan, S. and Borgia, M. and Rose, J..Needing Primary Care But Not Getting It: The Role of Trust, Stigma and Organizational Obstacles reported by Homeless Veterans.BMC Public Health.2015;204(3):1019-31. | Intervention |
| Oliveira, D. M. and Expedito, A. C. and Aleixo, M. T. and Carneiro, N. S. and Jesus, M. C. P. and Merighi, M. A. B..Needs, expectations and care production of people in street situation.Advanced Emergency Nursing Journal.2018;24():2689-2697. | No outcomes reported |
| Von Holtz, L. A. H. and Frasso, R. and Lu, Y. and Hanlon, A. L. and Dowshen, N. L..New media use and health information-seeking behavior among homeless adolescents.J Am Board Fam Med.2015;7(2):S55. | Publication type |
| Post, L. A. and Vaca, F. E. and Doran, K. M. and Luco, C. and Naftilan, M. and Dziura, J. and Brandt, C. and Bernstein, S. and Jagminas, L. and D'Onofrio, G..New media use by patients who are homeless: the potential of mHealth to build connectivity.Community Ment Health J.2013;39(9):e195. | No outcomes reported |
| O'Toole, T. P. and Bourgault, C. and Johnson, E. E. and Redihan, S. G. and Borgia, M. and Aiello, R. and Kane, V..New to care: demands on a health system when homeless veterans are enrolled in a medical home model.Cancer Causes Control.2013;11():S374-9. | No outcomes reported |
| Dickins, K. and Buchholz, S. W. and Ingram, D. and Hamilton, R. J. and Braun, L. T. and Karnik, N. S. and Earle, M.."Now that you've got that coverage": Promoting use of a regular source of primary care among homeless persons.International Journal of Pharmacy Practice.2019;124(2):158-166. | Population |
| Park, T. and Issa, M. and Mikhail, M. and Duong, S. and Bedi, H. and Jiang, S. and Wylson, V. and Lebovic, G. and Lichter, M..Ophthalmic findings in marginally housed women in a Canadian city.PLoS One.2024;10(1):12-18. | No outcomes reported |
| Yusuf, H. and Golkari, A. and Kaddour, S..Oral health of people experiencing homelessness in London: a mixed methods study.Int J Equity Health.2023;25(1):1701. | Population |
| Concannon, K. and Linden, H. and Thayer, H. and Baik, C..Outcomes and Quality Measures Among Homeless Lung Cancer Patients In A Single Institution.Front Psychiatry.2019;20(1):246. | Publication type |
| Johnsen, S. and Cuthill, F. and Blenkinsopp, J..Outreach-based clinical pharmacist prescribing input into the healthcare of people experiencing homelessness: a qualitative investigation.Nurs Res.2021;9(1):7. | Population |
| Hudson, B. and Shulman, C. and Dzeng, E. and Burnett, A..Palliative Care Access for People in the UK Who Are Experiencing Homelessness and Who Have No Recourse to Public Funds.J Prev Med Hyg.2022;94(1):57-58. | Publication type |
| Bazzi, A. R. and Shaw, L. C. and Biello, K. B. and Vahey, S. and Brody, J. K..Patient and Provider Perspectives on a Novel, Low-Threshold HIV PrEP Program for People Who Inject Drugs Experiencing Homelessness.J Subst Abuse Treat.2023;20(4):913-921. | Population |
| Daly, B. and Newton, J. T. and Batchelor, P..Patterns of dental service use among homeless people using a targeted service.J Urban Health.2010;74(1):45-51. | Population |
| Blonigen, D. and Smelson, D. and Smith, J. and Baldwin, N. and McInnes, D. K. and Raikov, I. and Weber, J. and Hyde, J..Peer support and whole health coaching to address the healthcare needs of homeless veterans: a pilot study.J Addict Dis.2022;50(1):331. | Population |
| Nguyen, M. A. H. and Reitzel, L. R. and Kendzor, D. E. and Businelle, M. S..Perceived cessation treatment effectiveness, medication preferences, and barriers to quitting among light and moderate/heavy homeless smokers.J Fam Med.2015;37():341-345. | No outcomes reported |
| Milligan, R. and Wingrove, B. K. and Richards, L. and Rodan, M. and Monroe-Lord, L. and Jackson, V. and Hatcher, B. and Harris, C. and Henderson, C. and Johnson, A. A..Perceptions about prenatal care: views of urban vulnerable groups.J Adolesc.2002;107():25. | Population |
| Kerman, N. and Gran-Ruaz, S. and Lawrence, M. and Sylvestre, J..Perceptions of Service Use Among Currently and Formerly Homeless Adults with Mental Health Problems.PLoS One.2019;33(5):777-783. | Population |
| Paudyal, V. and Stewart, D. and MacLure, K. and Buchanan, C. and MacLeod, J. and Wilson, L..Perspectives of homeless patients on their prescribed medicines.Health Soc Care Community.2015;58():6-7. | Publication type |
| Farmer, N. and McPherson, A. and Thomson, J. and Lowrie, R..Perspectives of people experiencing homelessness with recent non-fatal street drug overdose on the Pharmacist and Homeless Outreach Engagement and Non-medical Independent prescribing Rx (PHOENIx) intervention.BMC Fam Pract.2024;8(5):e0302988. | No outcomes reported |
| Lowrie, R. and Stock, K. and Lucey, S. and Knapp, M. and Williamson, A. and Montgomery, M. and Lombard, C. and Maguire, D. and Allan, R. and Blair, R. and Paudyal, V. and Mair, F. S..Pharmacist led homeless outreach engagement and non-medical independent prescribing (Rx) (PHOENIx) intervention for people experiencing homelessness: a non- randomised feasibility study.Health Soc Care Community.2021;119(1):19. | No outcomes reported |
| Vickery, K. D. and Gelberg, L. and Hyson, A. R. and Strother, E. and Carter, J. and Oranday Perez, O. and Franco, M. and Kavistan, S. and Gust, S. and Adair, E. and Anderson-Campbell, A. and Brito, L. and Butler, A. and Robinson, T. and Connett, J. and Evans, M. D. and Emmons, K. M. and Comulada, W. S. and Busch, A. M..Pilot trial results of D-HOMES: a behavioral-activation based intervention for diabetes medication adherence and psychological wellness among people who have been homeless.J Clin Psychiatry.2024;115():1329138. | Population |
| Rew, L. and Chambers, K. B. and Kulkarni, S..Planning a sexual health promotion intervention with homeless adolescents.JCO Oncol Pract.2002;11(3):168-74. | Intervention |
| Ly, T. D. A. and Perieres, L. and Hoang, V. T. and Dao, T. L. and Gautret, P..Pneumococcal infections and homelessness.J Addict Med.2021;18(4):E950-e957. | No outcomes reported |
| Hudson, A. L. and Nyamathi, A. and Slagle, A. and Greengold, B. and Griffin, D. K. and Khalilifard, F. and Gedzoff, D. and Reid, C..The power of the drug, nature of support, and their impact on homeless youth.Journal of Social Distress and the Homeless.2009;1(4):356-65. | No outcomes reported |
| Ojo-Fati, O. and Thomas, J. L. and Vogel, R. I. and Ogedegbe, O. and Jean-Louis, G. and Okuyemi, K. S..Predictors of Adherence to Nicotine Replacement Therapy (Nicotine Patch) Among Homeless Persons Enrolled in a Randomized Controlled Trial Targeting Smoking Cessation.Res Social Adm Pharm.2016;11(7):. | No outcomes reported |
| Hathazi, D. and Lankenau, S. E. and Sanders, B. and Jackson Bloom, J..Pregnancy and sexual health among homeless young injection drug users.PLoS One.2009;4(2):339-55. | Intervention |
| Winetrobe, H. and Rhoades, H. and Barman-Adhikari, A. and Cederbaum, J. and Rice, E. and Milburn, N..Pregnancy attitudes, contraceptive service utilization, and other factors associated with Los Angeles homeless youths' use of effective contraception and withdrawal.Dialogues Health.2013;45(6):314-22. | No outcomes reported |
| McInnes, D. K. and Fix, G. M. and Solomon, J. L. and Petrakis, B. A. and Sawh, L. and Smelson, D. A..Preliminary needs assessment of mobile technology use for healthcare among homeless veterans.Int J Environ Res Public Health.2015;182():e1096. | Population |
| Yim, L. C. and Leung, H. C. and Chan, W. C. and Lam, M. H. and Lim, V. W..Prevalence of Mental Illness among Homeless People in Hong Kong.J Health Care Poor Underserved.2015;9(10):e0140940. | Intervention |
| Pooprasert, P. and Ahnood, D. and Parmar, T. and Wang, W. and Young-Zvandasara, T. and Morgan, J..Prevalence of refractive error, visual impairment and access to eyecare for the homeless in Wales, United Kingdom.Int J Radiat Oncol Biol Phys.2021;30(10):2727-2732. | No outcomes reported |
| MacKinnon, L. and Kerman, N. and Socías, M. E. and Brar, R. and Bardwell, G..Primary care embedded within permanent supportive housing for people who use substances: A qualitative study examining healthcare access in Vancouver, Canada.Int J Tuberc Lung Dis.2022;5(6):e5062-e5073. | Population |
| Crane, M. and Warnes, A. M..Primary health care services for single homeless people: defects and opportunities.Ann Fam Med.2001;19(3):272-6. | Population |
| Campbell, D. J. and O'Neill, B. G. and Gibson, K. and Thurston, W. E..Primary healthcare needs and barriers to care among Calgary's homeless populations.Epidemiol Infect.2015;24():139. | Population |
| Steward, J. and Holt, C. L. and Pollio, D. E. and Austin, E. L. and Johnson, N. and Gordon, A. J. and Kertesz, S. G..Priorities in the primary care of persons experiencing homelessness: convergence and divergence in the views of patients and provider/experts.Future Virology.2016;32():153-8. | Population |
| Lamanna, D. and Stergiopoulos, V. and Durbin, J. and O'Campo, P. and Poremski, D. and Tepper, J..Promoting continuity of care for homeless adults with unmet health needs: The role of brief interventions.Front Public Health.2018;24(1):56-64. | Population |
| Sajatovic, M. and Levin, J. and Ramirez, L. F. and Hahn, D. Y. and Tatsuoka, C. and Bialko, C. S. and Cassidy, K. A. and Fuentes-Casiano, E. and Williams, T. D..Prospective trial of customized adherence enhancement plus long-acting injectable antipsychotic medication in homeless or recently homeless individuals with schizophrenia or schizoaffective disorder.Journal of Pediatric and Adolescent Gynecology.2013;83(12):1249-55. | Population |
| Mayo, Z. S. and Kilic, S. S. and Weleff, J. and Parker, S. M. and Strzalka, C. and Phelan, M. and Mian, O. Y. and Stephans, K. L. and Suh, J. H. and Tendulkar, R. D..Prostate Cancer Screening Disparities in Persons Experiencing Homelessness.Br J Gen Pract.2022;18(11):e1866-e1873. | Publication type |
| Kennedy, A. J. and George, J. S. and Rossetti, G. and Brown, C. O. and Ragins, K. and Dadiomov, D. and Trotzky-Sirr, R. and Sanchez, G. and Llamas, H. and Hurley, B..Providing Low-barrier Addiction Treatment Via a Telemedicine Consultation Service During the COVID-19 Pandemic in Los Angeles, County: An Assessment 1 Year Later.Br J Gen Pract.2023;(1):e64-e66. | No outcomes reported |
| Cox, S. N. and Thuo, N. B. and Rogers, J. H. and Meehan, A. A. and Link, A. C. and Martinez, M. and Lo, N. K. and Manns, B. J. and Ogokeh, C. and Chow, E. J. and Rolfes, M. A. and Mosites, E. and Al Achkar, M. and Chu, H..A qualitative analysis of COVID-19 vaccination intent, decision-making, and recommendations to increase uptake among residents and staff in six homeless shelters in Seattle, WA, USA.Aids and Behavior.2024;20(2):316-328. | Population |
| Moczygemba, L. R. and Kennedy, A. K. and Marks, S. A. and Goode, J. V. and Matzke, G. R..A qualitative analysis of perceptions and barriers to therapeutic lifestyle changes among homeless hypertensive patients.American Journal of Public Health.2013;192(4):467-81. | Intervention |
| Powell, P. V..Qualitative assessment in the evaluation of the Edinburgh primary health care scheme for single homeless hostel dwellers.Nicotine Tob Res.1988;60(3):185-196. | Population |
| Celeste-Villalvir, A. and Wilkerson, J. M. and Markham, C. and Rodriguez, L. and Schick, V..A qualitative investigation of organizational challenges and facilitators to screening individuals experiencing homelessness for hepatitis C virus (HCV) in Houston, Texas.Ir J Med Sci.2022;6(8):e0273302. | Population |
| Celeste-Villalvir, A. and Wilkerson, J. M. and Markham, C. and Rodriguez, L. and Schick, V..A qualitative investigation of organizational challenges and facilitators to screening individuals experiencing homelessness for hepatitis C virus (HCV) in Houston, Texas.BMC Health Serv Res.2022;11():100058. | Population |
| Asgary, R. and Sckell, B. and Alcabes, A. and Naderi, R. and Schoenthaler, A. and Ogedegbe, G..Rates and Predictors of Uncontrolled Hypertension Among Hypertensive Homeless Adults Using New York City Shelter-Based Clinics.Int J Ment Health Nurs.2016;51(1):41-6. | No outcomes reported |
| Conway, B. and Rodriguez-Tajes, S. and Garcia-Retortillo, M. and Pérez-Hernandez, P. and Teti, E. and Ryan, P. and Fraser, C. and MacEdo, G. and Morano Amado, L. E. and Lédinghen, V. D. and Fenech, M. and Martins, A. and Guerra-Veloz, M. F. and Ntalla, I. and Ramroth, H. and Vanstraelen, K. and Hernandez, C. and Mertens, M. and O'Loan, J..Real-world evidence of sofosbuvir/velpatasvir as an effective and simple hepatitis C virus treatment and elimination tool in homeless populations.Children and Youth Services Review.2022;1(2):77-86. | No outcomes reported |
| Gaudet, B. and Liu, N. and Downen, J. and Jarvill, T. and Zemanek, C. and Karn, M. and Cuadrado, H. and Quinones, J..Relationship Between Sexual Activity, Contraceptive Utilization and Biopsychosocial Characteristics Among Homeless Adolescents.CMAJ. Canadian Medical Association Journal.2021;12(2):272. | Population |
| Smith, K. G. and Paudyal, V. and MacLure, K. and Forbes-McKay, K. and Buchanan, C. and Wilson, L. and MacLeod, J. and Smith, A. and Stewart, D..Relocating patients from a specialist homeless healthcare centre to general practices: a multi-perspective study.Int J Drug Policy.2018;187(667):e105-e113. | No outcomes reported |
| Howells, K. and Amp, M. and Burrows, M. and Brown, J. and Brennan, R. and Dickinson, J. and Jackson, S. and Yeung, W. L. and Ashcroft, D. and Campbell, S. and Blakeman, T. and Sanders, C..Remote primary care during the COVID-19 pandemic for people experiencing homelessness: a qualitative study.J Subst Abuse Treat.2022;182(720):e492-e500. | Population |
| Ensign, J..Reproductive health of homeless adolescent women in Seattle, Washington, USA.Aids.2000;31(2):133-51. | Population |
| Galvin, A. M. and Akpan, I. N. and Lewis, M. A. and Walters, S. T. and Thompson, E. L..Reproductive Interconception Care Among Women Recently Pregnant and Homeless: A Qualitative Analysis.JMIR Mhealth Uhealth.2024;8(2):302-310. | Population |
| Maria, D. S. and Nyamathi, A. and Lightfoot, M. and Paul, M. and Quadri, Y. and Padhye, N. and Businelle, M. and Fernandez-Sanchez, H. and Jones, J. T..Results of a Randomized Wait-List Controlled Trial of CAYA: A Nurse Case Management HIV Prevention Intervention for Youth Experiencing Homelessness.Nicotine Tob Res.2024;191():. | Population |
| McInnes, D. K. and Petrakis, B. A. and Gifford, A. L. and Rao, S. R. and Houston, T. K. and Asch, S. M. and O'Toole, T. P..Retaining Homeless Veterans in Outpatient Care: A Pilot Study of Mobile Phone Text Message Appointment Reminders.Int J Environ Res Public Health.2014;16():S588-S594. | Population |
| Richards, C. M. and Sharif, F. and Eischen, S. and Thomas, J. and Wang, Q. and Guo, H. and Luo, X. and Okuyemi, K..Retention of Homeless Smokers in the Power to Quit Study.Contraception.2015;16(9):1104-11. | No outcomes reported |
| O'Carroll, A. and Irving, N. and O'Neill, J. and Flanagan, E..A review of a GP registrar-run mobile health clinic for homeless people.Front Oral Health.2017;187(3):541-546. | Population |
| Campbell, D. J. and Gibson, K. and O'Neill, B. G. and Thurston, W. E..The role of a student-run clinic in providing primary care for Calgary's homeless populations: a qualitative study.AIDS Behav.2013;197():277. | Population |
| Kalofonos, I. and McCoy, M. and Altman, L. and Gelberg, L. and Hamilton, A. B. and Gabrielian, S..A Sanctioned Encampment as a Strategy for Increasing Homeless Veterans' Access to Housing and Healthcare During the COVID-19 Pandemic.Br Dent J.2023;11():857-864. | Intervention |
| Jackson, Y. L. and Wuillemin, T. and Adler, D. and Janssens, J. P..Screening for active tuberculosis in an urban shelter for homeless in switzerland: A prospective study..2017;24(1):139-140. | Publication type |
| Parsell, C. and Ten Have, C. and Denton, M. and Walter, Z..Self-management of health care: multimethod study of using integrated health care and supportive housing to address systematic barriers for people experiencing homelessness.Soc Sci Med.2018;63(3):303-308. | Population |
| Muir-Cochrane, E. and Fereday, J. and Jureidini, J. and Drummond, A. and Darbyshire, P..Self-management of medication for mental health problems by homeless young people.PRiMER.2006;80(3):163-70. | Intervention |
| Rudolph, K. A. and Stewart, M. and Borba, C. P. C.."Shelter is Stressing Me Out": Challenges Meeting Health Care Needs of Older Adults in Congregate Shelters.Am J Orthopsychiatry.2023;8(3):1003-1020. | Intervention |
| Podymow, T. and Turnbull, J. and Coyle, D. and Yetisir, E. and Wells, G..Shelter-based managed alcohol administration to chronically homeless people addicted to alcohol.J Gen Intern Med.2006;189(1):45-49. | No outcomes reported |
| Pauly, B. B..Shifting moral values to enhance access to health care: harm reduction as a context for ethical nursing practice.Public Health Nurs.2008;16(3):195-204. | Population |
| Thompson, E. L. and Galvin, A. M. and Garg, A. and Diener, A. and Deckard, A. and Griner, S. B. and Kline, N. S..A socioecological perspective to contraceptive access for women experiencing homelessness in the United States.Digit Health.2023;83():109991. | Population |
| Paisi, M. and Witton, R. and Withers, L. and Plessas, A. and Burrows, M. and Morrison, S. and McDonald, L. and Kay, E..Strategies to improve oral health behaviours and dental access for people experiencing homelessness: a qualitative study.British Journal of Social Work.2020;33():. | Population |
| Bashir, K. and Ouedraogo, M. O. and Dharma, C. and Sobers, M. and Atukorale, V. and Mauer-Vakil, D. and Ataullahjan, A. and Fadel, S. A. and Allin, S..Strengthening access to and confidence in COVID-19 vaccines among equity-deserving populations across Canada: An exploratory qualitative study.Harm Reduct J.2024;178():. | Population |
| Teaw, S. and Annapureddy, D. and Wang, E. and Speed, S. and Gimpel, N..A Student-Led Patient Navigation Program: Assessing the Goals of Individuals Experiencing Homelessness.BMC Public Health.2024;26():50. | No outcomes reported |
| Baggett, T. P. and Yaqubi, A. and Berkowitz, S. A. and Kalkhoran, S. M. and McGlave, C. and Chang, Y. and Campbell, E. G. and Rigotti, N. A..Subsistence difficulties are associated with more barriers to quitting and worse abstinence outcomes among homeless smokers: evidence from two studies in Boston, Massachusetts.Nicotine Tob Res.2018;40(1):463. | Intervention |
| Dickins, K. A. and Buchholz, S. W. and Ingram, D. and Braun, L. T. and Hamilton, R. J. and Earle, M. and Karnik, N. S..Supporting Primary Care Access and Use among Homeless Persons.Contemp Clin Trials Commun.2020;51(6):335-357. | Population |
| Narendorf, S. C. and Munson, M. R. and Washburn, M. and Fedoravicius, N. and Wagner, R. and Flores, S. K..Symptoms, circumstances, and service systems: Pathways to psychiatric crisis service use among uninsured young adults.Journal of the American Academy of Orthopaedic Surgeons.2017;157(5):585-596. | Population |
| O'Toole, T. P. and Johnson, E. E. and Borgia, M. L. and Rose, J..Tailoring Outreach Efforts to Increase Primary Care Use Among Homeless Veterans: Results of a Randomized Controlled Trial.Can J Nurs Res.2015;61(7):886-98. | No outcomes reported |
| Lashley, M..A targeted testing program for tuberculosis control and prevention among Baltimore city's homeless population.Int J Circumpolar Health.2007;31(1):34-9. | No outcomes reported |
| Meray, V. and Motorwala, A. and Sellers, K. P. and Suarez, A. and Schneider, G. W..Telehealth Monitoring for Individuals Experiencing Homelessness During the Early COVID-19 Pandemic: An Innovative Clinical and Medical Education Model.PLOS Glob Public Health.2022;178(11):e31528. | No outcomes reported |
| Joseph, K. and Cardenas, F. and Brudney, R. and Haukoos, J. and Ray, L. and Whitfield, J..Test results pending at discharge among emergency department patients experiencing homelessness.Vaccine X.2024;29():190-195. | Population |
| Kershaw, K. and Martelly, L. and Stevens, C. and McInnes, D. K. and Silverman, A. and Byrne, T. and Aycinena, D. and Sabin, L. L. and Garvin, L. A. and Vimalananda, V. G. and Hass, R..Text messaging to increase patient engagement in a large health care for the homeless clinic: Results of a randomized pilot study.J Addict Med.2022;15():20552076221129729. | Population |
| Rhoades, H. and Wenzel, S. and Winetrobe, H. and Ramirez, M. and Wu, S. and Carranza, A. and Dent, D. and Caraballo Jones, M..A text messaging-based intervention to increase physical activity among persons living in permanent supportive housing: Feasibility and acceptability findings from a pilot study.Int J Tuberc Lung Dis.2019;42():2055207619832438. | Population |
| Leonard, N. R. and Casarjian, B. and Fletcher, R. R. and Praia, C. and Sherpa, D. and Kelemen, A. and Rajan, S. and Salaam, R. and Cleland, C. M. and Gwadz, M. V..Theoretically-Based Emotion Regulation Strategies Using a Mobile App and Wearable Sensor Among Homeless Adolescent Mothers: Acceptability and Feasibility Study.Addictive Disorders and their Treatment.2018;16(1):e1. | Population |
| Farmer, N. and McPherson, A. and Thomson, J. and Reilly, F. and Williamson, A. and Lowrie, R..There's No Hope for Any Kind of Decent Life': A Qualitative Study to Explore the Perspectives of People Experiencing Homelessness with a Recent Non-Fatal Overdose in Scotland.J Natl Black Nurses Assoc.2024;83(2):548-567. | Intervention |
| Parkes, T. and Carver, H. and Masterton, W. and Falzon, D. and Dumbrell, J. and Grant, S. and Wilson, I..'They already operated like it was a crisis, because it always has been a crisis': a qualitative exploration of the response of one homeless service in Scotland to the COVID-19 pandemic.BMC Prim Care.2021;35(1):26. | Population |
| Kumar, S. and Gupte, H. A. and Isaakidis, P. and Mishra, J. K. and Munjattu, J. F.."They don't like us….": Barriers to antiretroviral and opioid substitution therapy among homeless HIV positive people who inject drugs in Delhi: A mixed method study.Am J Public Health.2018;22(8):e0203262. | Population |
| Lamarche, L. and Scallan, E. and Mak, O. and Howden, J. and Bodkin, C. and Nussey, L. and Wolf, K. and Ans, J. and Delottinville, D. and O'Shea, T. and Lennox, R.."They forgot about us": experiences of the COVID-19 pandemic among people deprived of housing in an urban centre in Ontario, Canada.BMC Public Health.2023;10(5):796-805. | Intervention |
| McDaniel, M. and Sundaram, S. and Manjanatha, D. and Odes, R. and Lerman, P. and Handley, M. A. and Coffin, P. O. and Myers, J. J. and Goldman, M. L.."They made me feel like I mattered": a qualitative study of how mobile crisis teams can support people experiencing homelessness.Int J Environ Res Public Health.2024;21(1):2183. | Intervention |
| Vijayaraghavan, M. and Tieu, L. and Ponath, C. and Guzman, D. and Kushel, M..Tobacco Cessation Behaviors Among Older Homeless Adults: Results From the HOPE HOME Study.Drugs-Education Prevention and Policy.2016;19(8):1733-9. | No outcomes reported |
| Miller, J. and Cuby, J. and Hall, S. M. and Stitzer, M. and Kushel, M. and Appiah, D. and Vijayaraghavan, M..Tobacco use behaviors and views on engaging in clinical trials for tobacco cessation among individuals who experience homelessness.J Am Geriatr Soc.2023;84():101094. | Intervention |
| D'Souza, M. S. and Mirza, N. A..Towards Equitable Health Care Access: Community Participatory Research Exploring Unmet Health Care Needs of Homeless Individuals.PLoS One.2022;21(4):451-463. | Population |
| Schmidt, R. and Hrenchuk, C. and Bopp, J. and Poole, N..Trajectories of women's homelessness in Canada's 3 northern territories.American Behavioral Scientist.2015;10():29778. | Intervention |
| Heyd, A. and Heffernan, C. and Storey, K. and Wild, T. C. and Long, R..Treating latent tuberculosis infection (LTBI) with isoniazid and rifapentine (3HP) in an inner-city population with psychosocial barriers to treatment adherence: A qualitative descriptive study.J Pain Symptom Manage.2021;20(12):e0000017. | Population |
| Sánchez-Arcilla, I. and Vílchez, J. M. and García de la Torre, M. and Fernández, X. and Noguerado, A..[Treatment of latent tuberculosis among homeless population. Comparison between wo therapeutic approaches].JAMA Ophthalmol.2004;23(2):57-9. | Population |
| Cox, S. N. and Rogers, J. H. and Thuo, N. B. and Meehan, A. and Link, A. C. and Lo, N. K. and Manns, B. J. and Chow, E. J. and Al Achkar, M. and Hughes, J. P. and Rolfes, M. A. and Mosites, E. and Chu, H. Y..Trends and factors associated with change in COVID-19 vaccination intent among residents and staff in six Seattle homeless shelters, March 2020 to August 2021.Saude E Sociedade.2022;11():100232. | Population |
| Rogers, J. H. and Cox, S. N. and Hughes, J. P. and Link, A. C. and Chow, E. J. and Fosse, I. and Lukoff, M. and Shim, M. M. and Uyeki, T. M. and Ogokeh, C. and Jackson, M. L. and Boeckh, M. and Englund, J. A. and Mosites, E. and Rolfes, M. A. and Chu, H. Y..Trends in COVID-19 vaccination intent and factors associated with deliberation and reluctance among adult homeless shelter residents and staff, 1 November 2020 to 28 February 2021 - King County, Washington.Ssm-Qualitative Research in Health.2022;111(1):122-132. | Population |
| Samuel, L. and Caygill-Walsh, R. and Suen, L. W. and Mohebbi, S. and Geier, M..Triple Threat: Response to the Crises of COVID-19, Homelessness, and Opioid Use Disorder With a Novel Approach to Buprenorphine Delivery: A Case Series.Journal of Social Distress and the Homeless.2022;42(6):733-735. | No outcomes reported |
| Southern, A. and Premaratne, N. and English, M. and Balazs, J. and O'Sullivan, D..Tuberculosis among homeless people in London: an effective model of screening and treatment.Public Health.1999;28(11):1001-8. | Publication type |
| Borda, J. P. and Zuleta, P..Ulysses Coercion through Psychiatric Advanced Directives in Homeless People with Substance Use Disorder: A Qualitative Study of the Colombian Perspective.Res Social Adm Pharm.2021;10(1):21-28. | Intervention |
| Hwang, S. W. and Ueng, J. J. and Chiu, S. and Kiss, A. and Tolomiczenko, G. and Cowan, L. and Levinson, W. and Redelmeier, D. A..Universal health insurance and health care access for homeless persons..2010;40(8):1454-61. | No outcomes reported |
| Thorndike, A. L. and Yetman, H. E. and Thorndike, A. N. and Jeffrys, M. and Rowe, M..Unmet health needs and barriers to health care among people experiencing homelessness in San Francisco's Mission District: a qualitative study..2022;8(1):1071. | Population |
| Carroll, Á and O'Brien, S. and Harrington, D. and Cheallaigh, C. N. and Lawlee, A. M. and Sukumar, P..The Unmet Rehabilitation Needs in an Inclusion Health Integrated Care Programme for Homeless Adults in Dublin, Ireland..2021;31(15):. | No outcomes reported |
| Pajka, S. E. and Kushel, M. and Handley, M. A. and Olsen, P. and Li, B. and Enriquez, C. and Kaplan, L. and Sudore, R. L..Using behavioral theory to adapt advance care planning for homeless-experienced older adults in permanent supportive housing..2023;13B(8):2615-2626. | Population |
| Grewal, E. K. and Campbell, R. B. and Booth, G. L. and McBrien, K. A. and Hwang, S. W. and O'Campo, P. and Campbell, D. J. T..Using concept mapping to prioritize barriers to diabetes care and self-management for those who experience homelessness..2021;34(1):158. | Population |
| Onwubiko, U. and Wall, K. and Sales, R. M. and Holland, D. P..Using Directly Observed Therapy (DOT) for latent tuberculosis treatment - A hit or a miss? A propensity score analysis of treatment completion among 274 homeless adults in Fulton County, GA..2019;96(6):e0218373. | No outcomes reported |
| Wenzel, S. L. and Leake, B. D. and Andersen, R. M. and Gelberg, L..Utilization of birth control services among homeless women..2001;31(1):14-34. | No outcomes reported |
| Klop, H. T. and van Dongen, S. I. and Francke, A. L. and de Veer, A. J. E. and Rietjens, J. A. C. and Gootjes, J. R. G. and Onwuteaka-Philipsen, B. D..The Views of Homeless People and Health Care Professionals on Palliative Care and the Desirability of Setting Up a Consultation Service: A Focus Group Study..2018;114(3):327-336. | Population |
| Noel, C. W. and Fung, H. and Srivastava, R. and Lebovic, G. and Hwang, S. W. and Berger, A. and Lichter, M..Visual impairment and unmet eye care needs among homeless adults in a Canadian city..2015;(4):455-60. | No outcomes reported |
| do Vale, A. R. and Dalla Vecchia, M.."We are the UPA ourselves": social support networks on the health care for the homeless in a smalltown..2019;(1):222-234. | Intervention |
| Jenkinson, J. I. R. and Hwang, S. W. and Strike, C. and Di Ruggiero, E.."We don't have a good system for people who don't have a home and don't need a hospital": Contextualizing the hospital discharge process for people experiencing homelessness in Toronto, Canada..2022;():. | Population |
| Pendyal, A. and Rosenthal, M. S. and Spatz, E. S. and Cunningham, A. and Bliesener, D. and Keene, D. E.."When you're homeless, they look down on you": A qualitative, community-based study of homeless individuals with heart failure..2021;(1):80-85. | Population |
| Thurman, W. and Moczygemba, L. R. and Baffoe, J. O.."Without my medication, I'm a wreck": Photo-elicitation to explore medication use among people experiencing homelessness..2022;(7):3149-3157. | Intervention |
| Groton, D. B. and Leavitt, M. A. and Opalinski, A. S.."You got to eat, but then what you are eating, it's going to kill you": Living with hypertension while experiencing homelessness..2021;(2):160-166. | Intervention |
| McElyea, J. and Bistransin, K. and Bana, S. and Alvarez, K. S. and Brown, L. S. and Persaud, D. and King, H..Impact of a clinical pharmacist within an HIV PrEP program for patients experiencing homelessness.J Prim Care Community Health.2023;84(1):324-329. | Not retrieved |
| Polzer, E. R. and Thomas, S. M. and Kinney, A. R. and Penzenik, M. E. and Monteith, L. L. and Brenner, L. A. and Holliday, R..A thematic analysis of perceptions of VHA emergency care and suicide risk assessment among Veterans experiencing homelessness.Arch Intern Med.2024;22():. | Not retrieved |
| Niu, R. J. and Egan, C. and Fang, C. and Duru, N. and Alley, M. C. and Freccero, D. M. and Smith, E. L..Total Joint Arthroplasty in Homeless Patients at an Urban Safety Net Hospital.Int J Equity Health.2022;84(11):523-527. | No outcomes reported |
| Baldwin, D. M. and Williams-Brown, S..Uncovering homeless African-American women's knowledge of breast cancer and their use of breast cancer screening services.Public Health Nurs.2005;(1):24-30. | Not retrieved |
| Feng, R. C. and Lathrop, B. and Santibanez, S..What keeps you coming back? Health care experience and determinants of consistent follow-up among persons experiencing homelessness in Atlanta, GA..2024;():. | Not retrieved |
| Magwood O, Leki VY, Kpade V, Saad A, Alkhateeb Q, Gebremeskel A, et al. Common trust and personal safety issues: A systematic review on the acceptability of health and social interventions for persons with lived experience of homelessness. PLoS One. 2019;14(12):e0226306. | No outcomes reported |
| Parpouchi M, Moniruzzaman A, Rezansoff SN, Russolillo A, Somers JM. Characteristics of adherence to methadone maintenance treatment over a 15-year period among homeless adults experiencing mental illness. Addict Behav Rep. 2017;6:106-11. | No outcomes reported |
| Miguel AQC, Simoes V, Yamauchi R, Madruga CS, da Silva CJ, Laranjeira RR, et al. Acceptability and feasibility of incorporating contingency management into a public treatment program for homeless crack cocaine users in Brazil: A pilot study. Exp Clin Psychopharmacol. 2022;30(5):507-13. | No outcomes reported |
| Okuyemi KS, Thomas JL, Hall S, Nollen NL, Richter KP, Jeffries SK, et al. Smoking cessation in homeless populations: a pilot clinical trial. Nicotine Tob Res. 2006;8(5):689-99. | No outcomes reported |
| Bangsberg DR, Ragland K, Monk A, Deeks SG. A single tablet regimen is associated with higher adherence and viral suppression than multiple tablet regimens in HIV+ homeless and marginally housed people. AIDS. 2010;24(18):2835-40 | No outcomes reported |
| Moss AR, Hahn JA, Perry S, Charlebois ED, Guzman D, Clark RA, et al. Adherence to highly active antiretroviral therapy in the homeless population in San Francisco: a prospective study. Clin Infect Dis. 2004;39(8):1190-8. | No outcomes reported |
| Bangsberg DR, Hecht FM, Charlebois ED, Zolopa AR, Holodniy M, Sheiner L, et al. Adherence to protease inhibitors, HIV-1 viral load, and development of drug resistance in an indigent population. AIDS. 2000;14(4):357-66. | No outcomes reported |
| Schwarz K, Garrett B, Lee J, Thompson D, Thiel T, Alter MJ, et al. Positive impact of a shelter-based hepatitis B vaccine program in homeless Baltimore children and adolescents. J Urban Health. 2008;85(2):228-38. | No outcomes reported |
| Jones AL, Hausmann LRM, Kertesz SG, Suo Y, Cashy JP, Mor MK, et al. Providing Positive Primary Care Experiences for Homeless Veterans Through Tailored Medical Homes: The Veterans Health Administration's Homeless Patient Aligned Care Teams. Med Care. 2019;57(4):270-8. | No outcomes reported |
| Gabrielian S, Jones AL, Hoge AE, deRussy AJ, Kim YI, Montgomery AE, et al. Enhancing Primary Care Experiences for Homeless Patients with Serious Mental Illness: Results from a National Survey. J Prim Care Community Health. 2021;12:2150132721993654. | No outcomes reported |
| Rosen AD, Howerton I, Brosnan HK, Stefanescu A, Gomih A, Ngo C, et al. Financial Incentives for COVID-19 Vaccines Among People Experiencing Homelessness. Am J Prev Med. 2023;65(1):12-8. | No outcomes reported |
| McCosker LK, Ware RS, Seale H, Hooshmand D, O'Leary R, Downes MJ. The effect of a financial incentive on COVID-19 vaccination uptake, and predictors of uptake, in people experiencing homelessness: A randomized controlled trial. Vaccine. 2024;42(10):2578-84. | No outcomes reported |
| Thomas I, Mackie P. Assessing the coverage and timeliness of coronavirus vaccination among people experiencing homelessness in Wales, UK: a population-level data-linkage study. BMC Public Health. 2023;23(1):1494. | No outcomes reported |
| Shariff SZ, Richard L, Hwang SW, Kwong JC, Forchuk C, Dosani N, et al. COVID-19 vaccine coverage and factors associated with vaccine uptake among 23 247 adults with a recent history of homelessness in Ontario, Canada: a population-based cohort study. Lancet Public Health. 2022;7(4):e366-e77. | No outcomes reported |
| Parashar S, Palmer AK, O'Brien N, Chan K, Shen A, Coulter S, et al. Sticking to it: the effect of maximally assisted therapy on antiretroviral treatment adherence among individuals living with HIV who are unstably housed. AIDS Behav. 2011;15(8):1612-22. | No outcomes reported |
| Chong MT, Yamaki J, Harwood M, d'Assalenaux R, Rosenberg E, Aruoma O, et al. Assessing health conditions and medication use among the homeless community in Long Beach, California. J Res Pharm Pract. 2014;3(2):56-61. | No outcomes reported |
| Rezansoff SN, Moniruzzaman A, Fazel S, Procyshyn R, Somers JM. Adherence to antipsychotic medication among homeless adults in Vancouver, Canada: a 15-year retrospective cohort study. Soc Psychiatry Psychiatr Epidemiol. 2016;51(12):1623-32. | No outcomes reported |
| Waddell CJ, Pellegrini Gj, Jr., Persad N, Filardo TD, Prasad N, Carson WC, et al. Minimally Invasive Blood Collection for an Mpox Serosurvey among People Experiencing Homelessness. J Appl Lab Med. 2024;9(5):1014-9. | No outcomes reported |
| Santa Maria D, Cuccaro P, Bender K, Cron S, Fine M, Sibinga E. Feasibility of a Mindfulness-Based Intervention with Sheltered Youth Experiencing Homelessness. Journal of Child and Family Studies. 2020;29(1):261-72. | No outcomes reported |
| Lindner AK, Sarma N, Rust LM, Hellmund T, Krasovski-Nikiforovs S, Wintel M, et al. Monitoring for COVID-19 by universal testing in a homeless shelter in Germany: a prospective feasibility cohort study. BMC Infect Dis. 2021;21(1):1241. | No outcomes reported |
| Nyamathi AM, Christiani A, Nahid P, Gregerson P, Leake B. A randomized controlled trial of two treatment programs for homeless adults with latent tuberculosis infection. Int J Tuberc Lung Dis. 2006;10(7):775-82. | No outcomes reported |
| Bell L, Whelan M, Lycett D, Fernandez E, Khera-Butler T, Kehal I, et al. Healthcare and housing provision for a UK homeless community: a qualitative service evaluation. Public Health. 2024;229:1-6. | No outcomes reported |
| Asgary R, Naderi R, Wisnivesky J. Opt-Out Patient Navigation to Improve Breast and Cervical Cancer Screening Among Homeless Women. J Womens Health (Larchmt). 2017;26(9):999-1003. | No outcomes reported |
| Bahrami S, Chang C, Alvarez KS, Lutek K, Nguyen S, Hegde A. Pharmacist impact on health outcomes in a homeless population. J Am Pharm Assoc (2003). 2020;60(3):485-90. | No outcomes reported |
| Facer BD, Bingham B, Fleisch SB, Walker JN, Ahmad M, Osmundson EC. Radiation Therapy Adherence Among Patients Experiencing Homelessness. Int J Radiat Oncol Biol Phys. 2021;109(4):1019-27. | No outcomes reported |
| Powell J, Ricco M, Naugle J, Magee C, Hassan H, Masson C, et al. Adherence to Hepatitis C Therapy in a Shelter-Based Education and Treatment Model Among Persons Experiencing Homelessness. Open Forum Infect Dis. 2021;8(10):ofab488. | No outcomes reported |
| Bekasi S, Girasek E, Gyorffy Z. Telemedicine in community shelters: possibilities to improve chronic care among people experiencing homelessness in Hungary. Int J Equity Health. 2022;21(1):181. | No outcomes reported |
| Janssens JP, Wuillemin T, Adler D, Jackson Y. Screening for tuberculosis in an urban shelter for homeless in Switzerland: a prospective study. BMC Infect Dis. 2017;17(1):347. | No outcomes reported |
| Pilote L, Tulsky JP, Zolopa AR, Hahn JA, Schecter GF, Moss AR. Tuberculosis Prophylaxis in the Homeless: A Trial to Improve Adherence to Referral. Archives of Internal Medicine. 1996;156(2):161-5. | No outcomes reported |

**Tables S4. Included studies – complete references**

| 1 | Chen JS, Nguyen AH, Malesker MA, Morrow LE: High-Risk Smoking Behaviors and Barriers to Smoking Cessation Among Homeless Individuals. Respir Care 2016, 61(5):640-645. |
| --- | --- |
| 2 | Nicholls MJ, Urada LA: Homelessness and polysubstance use: A qualitative study on recovery and treatment access solutions around an urban library in Southern California, USA. Health Soc Care Community 2022, 30(1):e175-e183. |
| 3 | Rubin SB, Vijayaraghavan M, Weiser SD, Tsoh JY, Cohee A, Delucchi K, Riley ED: Homeless women's perspectives on smoking and smoking cessation programs: A qualitative study. Int J Drug Policy 2021, 98103377. |
| 4 | Swartz N, Adnan T, Perea F, Baggett TP, Chatterjee A: "Sick and tired of being sick and tired": Exploring initiation of medications for opioid use disorder among people experiencing homelessness. J Subst Abuse Treat 2022, 138108752. |
| 5 | Baggett TP, McGlave C, Kruse GR, Yaqubi A, Chang Y, Rigotti NA: SmokefreeTXT for Homeless Smokers: Pilot Randomized Controlled Trial. JMIR Mhealth Uhealth 2019, 7(6):e13162. |
| 6 | Sestito SF, Rodriguez KL, Saba SK, Conley JW, Mitchell MA, Gordon AJ: Homeless veterans' experiences with substance use, recovery, and treatment through photo elicitation. Subst Abus 2017, 38(4):422-431. |
| 7 | Linnemayr S, Zutshi R, Shadel W, Pedersen E, DeYoreo M, Tucker J: Text Messaging Intervention for Young Smokers Experiencing Homelessness: Lessons Learned From a Randomized Controlled Trial. JMIR Mhealth Uhealth 2021, 9(4):e23989. |
| 8 | Salem BE, Klansek E, Morisky DE, Shin SS, Yadav K, Chang AH, Nyamathi AM: Acceptability and Feasibility of a Nurse-Led, Community Health Worker Partnered Latent Tuberculosis Medication Adherence Model for Homeless Adults. Int J Environ Res Public Health 2020, 17(22). |
| 9 | Chan B, Hulen E, Edwards ST, Geduldig A, Devoe M, Nicolaidis C, Korthuis PT, Saha S: Perceptions of Medically Complex Patients Enrolled in an Ambulatory Intensive Care Unit at a Healthcare-for-the-Homeless Clinic. J Am Board Fam Med 2025, 37(5):888-899. |
| 10 | Asgary R, Sckell B, Alcabes A, Naderi R, Ogedegbe G: Perspectives of cancer and cancer screening among homeless adults of New York City shelter-based clinics: a qualitative approach. Cancer Causes Control 2015, 26(10):1429-1438. |
| 11 | Moravac CC: Reflections of Homeless Women and Women with Mental Health Challenges on Breast and Cervical Cancer Screening Decisions: Power, Trust, and Communication with Care Providers. Front Public Health 2018, 630. |
| 12 | Neale J, Stevenson C: Positive and negative features of a computer assisted drug treatment program delivered by mentors to homeless drug users living in hostels. J Subst Abuse Treat 2014, 47(4):258-264. |
| 13 | Neale J, and Stevenson C: The use of computer-assisted therapy by homeless drug users living in hostels: An explorative qualitative study. Drugs: Education, Prevention and Policy 2014, 21(1):80-87. |
| 14 | Gelberg L, Browner CH, Lejano E, Arangua L: Access to women's health care: a qualitative study of barriers perceived by homeless women. Women Health 2004, 40(2):87-100. |
| 15 | Eapen DJ, Bergh R, Lucas S, Narendorf SC, Begun S, Santa Maria D: Experiences of pregnancy prevention among youth experiencing homelessness. Children and Youth Services Review 2023, 153107115. |
| 16 | Adedze M, Osei-Yeboah R, Morhe ESK, Ngambouk VP: Exploring Sexual and Reproductive Health Needs and Associated Barriers of Homeless Young Adults in Urban Ghana: A Qualitative Study. Sex Res Social Policy 2022, 19(3):1006-1019. |
| 17 | Kennedy S, Grewal M, Roberts EM, Steinauer J, Dehlendorf C: A qualitative study of pregnancy intention and the use of contraception among homeless women with children. J Health Care Poor Underserved 2014, 25(2):757-770. |
| 18 | Kachingwe ON, Anderson K, Houser C, Fleishman JL, Novick JG, Phillips DR, Aparicio EM: “She was there through the whole process:” Exploring how homeless youth access and select birth control. Children and Youth Services Review 2019, 101277-284. |
| 19 | Vickery KD, Ford BR, Gelberg L, Bonilla Z, Strother E, Gust S, Adair E, Montori VM, Linzer M, Evans MD, et al: The development and initial feasibility testing of D-HOMES: a behavioral activation-based intervention for diabetes medication adherence and psychological wellness among people experiencing homelessness. Front Psychol 2023, 141225777. |
| 20 | Weber JJ, Lee RC, Martsolf D: Experiences of Care in the Emergency Department Among a Sample of Homeless Male Veterans: A Qualitative Study. J Emerg Nurs 2020, 46(1):51-58. |
| 21 | McCallum R, Medved MI, Hiebert-Murphy D, Distasio J, Sareen J, Chateau D: Fixed Nodes of Transience: Narratives of Homelessness and Emergency Department Use. Qual Health Res 2020, 30(8):1183-1195. |
| 22 | Chinchilla M, Preston-Suni K, Jacobo E, Gabrielian S: Increasing Primary Care Engagement Among Homeless-Experienced Veterans Following an Emergency Department Visit: Qualitative Insights From Los Angeles County. J Prim Care Community Health 2024, 1521501319241296603. |
| 23 | Moczygemba LR, Thurman W, Tormey K, Hudzik A, Welton-Arndt L, Kim E: GPS Mobile Health Intervention Among People Experiencing Homelessness: Pre-Post Study. JMIR Mhealth Uhealth 2021, 9(11):e25553. |
| 24 | Partida D, Powell J, Ricco M, Naugle J, Magee C, Zevin B, Masson CL, Konadu Fokuo J, Gonzalez D, Khalili M: Formal Hepatitis C Education Increases Willingness to Receive Therapy in an On-site Shelter-Based HCV Model of Care in Persons Experiencing Homelessness. Open Forum Infect Dis 2022, 9(4):ofac103. |
| 25 | Masson CL, Fokuo JK, Anderson A, Powell J, Zevin B, Bush D, Khalili M: Clients' perceptions of barriers and facilitators to implementing hepatitis C virus care in homeless shelters. BMC Infect Dis 2020, 20(1):386. |
| 26 | Rado N, Bekasi S, Gyorffy Z: Health Technology Access and Peer Support Among Digitally Engaged People Experiencing Homelessness: Qualitative Study. JMIR Hum Factors 2024, 11e55415. |
| 27 | Oliveira MA, Boska GA, Oliveira MAF, Barbosa GC: Access to health care for people experiencing homelessness on Avenida Paulista: barriers and perceptions. Rev Esc Enferm USP 2021, 55e03744. |
| 28 | Christiani A, Hudson AL, Nyamathi A, Mutere M, Sweat J: Attitudes of homeless and drug-using youth regarding barriers and facilitators in delivery of quality and culturally sensitive health care. J Child Adolesc Psychiatr Nurs 2008, 21(3):154-163. |
| 29 | Ensign J, Panke A: Barriers and bridges to care: voices of homeless female adolescent youth in Seattle, Washington, USA. J Adv Nurs 2002, 37(2):166-172. |
| 30 | Darbyshire P, Muir-Cochrane E, Fereday J, Jureidini J, Drummond A: Engagement with health and social care services: perceptions of homeless young people with mental health problems. Health Soc Care Community 2006, 14(6):553-562. |
| 31 | Henderson MD, McCurry IJ, Deatrick JA, Lipman TH: Experiences of Adult Men Who Are Homeless Accessing Care: A Qualitative Study. J Transcult Nurs 2022, 33(2):199-207. |
| 32 | Ensign J, Gittelsohn J: Health and access to care: perspectives of homeless youth in Baltimore City, U.S.A. Soc Sci Med 1998, 47(12):2087-2099. |
| 33 | Lewis JH, Andersen RM, Gelberg L: Health care for homeless women. J Gen Intern Med 2003, 18(11):921-928. |
| 34 | Ramsay N, Hossain R, Moore M, Milo M, Brown A: Health Care While Homeless: Barriers, Facilitators, and the Lived Experiences of Homeless Individuals Accessing Health Care in a Canadian Regional Municipality. Qual Health Res 2019, 29(13):1839-1849. |
| 35 | Hudson AL, Nyamathi A, Greengold B, Slagle A, Koniak-Griffin D, Khalilifard F, Getzoff D: Health-seeking challenges among homeless youth. Nurs Res 2010, 59(3):212-218. |
| 36 | Nickasch B, Marnocha SK: Healthcare experiences of the homeless. J Am Acad Nurse Pract 2009, 21(1):39-46. |
| 37 | Mnkandla MM, Tshitangano TG, Mudau AG: Healthcare-Seeking Behaviors of Homeless Substance Users During the COVID-19 Lockdowns in Gauteng, South Africa: A COREQ-Based Report. In Social Sciences, vol. 12; 2023. |
| 38 | Mc Conalogue D, Maunder N, Areington A, Martin K, Clarke V, Scott S: Homeless people and health: a qualitative enquiry into their practices and perceptions. J Public Health (Oxf) 2021, 43(2):287-294. |
| 39 | Prado M, Goncalves M, Silva SSD, Oliveira PS, Santos KDS, Fortuna CM: Homeless people: health aspects and experiences with health services. Rev Bras Enferm 2021, 74(1):e20190200. |
| 40 | Wen CK, Hudak PL, Hwang SW: Homeless people's perceptions of welcomeness and unwelcomeness in healthcare encounters. J Gen Intern Med 2007, 22(7):1011-1017. |
| 41 | Woith WM, Kerber C, Astroth KS, Jenkins SH: Lessons from the Homeless: Civil and Uncivil Interactions with Nurses, Self-Care Behaviors, and Barriers to Care. Nurs Forum 2017, 52(3):211-220. |
| 42 | Haley RJ, Woodward KR: Perceptions of Individuals Who Are Homeless: Healthcare Access and Utilization in San Diego. Advanced Emergency Nursing Journal 2007, 29(4). |
| 43 | Eapen DJ, Bergh R, Narendorf SC, Santa Maria DM: Pregnancy and parenting support for youth experiencing homelessness. Public Health Nurs 2022, 39(4):728-735. |
| 44 | Kneck A, Mattsson E, Salzmann-Erikson M, Klarare A, in collaboration with Women Advisory Board for Inclusion H: "Stripped of dignity" - Women in homelessness and their perspectives of healthcare services: A qualitative study. Int J Nurs Stud 2021, 120103974. |
| 45 | McGonigle K, Carley T, Hoff C: Assessing Racial Disparities in HCV Infection and Care Outcomes in a Southern Urban Population. J Racial Ethn Health Disparities 2018, 5(5):1052-1058. |
| 46 | Etchin AG, LaCoursiere-Zucchero T, McDannold SE, McInnes DK: Dual use of Department of Veterans Affairs and community healthcare: Homeless veterans' experiences, perspectives, and perceptions. J Am Assoc Nurse Pract 2021, 33(11):991-998. |
| 47 | Bell L, Whelan M, Lycett D, Fernandez E, Khera-Butler T, Kehal I, Patel R: Healthcare and housing provision for a UK homeless community: a qualitative service evaluation. Public Health 2024, 2291-6. |
| 48 | Ramirez J, Petruzzi LJ, Mercer T, Gulbas LE, Sebastian KR, Jacobs EA: Understanding the primary health care experiences of individuals who are homeless in non-traditional clinic settings. BMC Prim Care 2022, 23(1):338. |
| 49 | Doroshenko A, Hatchette J, Halperin SA, MacDonald NE, Graham JE: Challenges to immunization: the experiences of homeless youth. BMC Public Health 2012, 12338. |
| 50 | Jenkinson JIR, Wigle J, Richard L, Tibebu T, Orkin AM, Thulien NS, Kiran T, Gogosis E, Crichlow F, Dyer AP, et al: Structural violence as a driver of COVID-19 vaccine hesitancy and low vaccine uptake among people experiencing homelessness in Toronto, Canada: A qualitative study. Soc Sci Med 2025, 365117588. |
| 51 | Grune J, Savelsberg D, Kobus M, Lindner AK, Herrmann WJ, Schuster A: Determinants of COVID-19 vaccine acceptance and access among people experiencing homelessness in Germany: A qualitative interview study. Front Public Health 2023, 111148029. |
| 52 | Coe AB, Moczygemba LR, Gatewood SB, Osborn RD, Matzke GR, Goode JV: Medication adherence challenges among patients experiencing homelessness in a behavioral health clinic. Res Social Adm Pharm 2015, 11(3):e110-120. |
| 53 | Richler MJ, Yousaf S, Hwang SW, Dewhurst NF: Descriptive study of homeless patients' perceptions that affect medication adherence. Am J Health Syst Pharm 2019, 76(17):1288-1295. |
| 54 | Nyamathi A, Shuler P: Factors Affecting Prescribed Medication Compliance of the Urban Homeless Adult. The Nurse Practitioner 1989, 14(8). |
| 55 | Paudyal V, MacLure K, Buchanan C, Wilson L, Macleod J, Stewart D: 'When you are homeless, you are not thinking about your medication, but your food, shelter or heat for the night': behavioural determinants of homeless patients' adherence to prescribed medicines. Public Health 2017, 1481-8. |
| 56 | Adkins EC, Zalta AK, Boley RA, Glover A, Karnik NS, Schueller SM: Exploring the potential of technology-based mental health services for homeless youth: A qualitative study. Psychol Serv 2017, 14(2):238-245. |
| 57 | Guenzel N, Jerreed I, Patrick H, Leeza S, and Hinrichsen S: Mental health, stigma, and barriers to care in a Midwestern sample of homeless individuals. Journal of Social Distress and Homelessness 2020, 29(2):102-109. |
| 58 | Adams EA, Parker J, Jablonski T, Kennedy J, Tasker F, Hunter D, Denham K, Smiles C, Muir C, O'Donnell A, et al: A Qualitative Study Exploring Access to Mental Health and Substance Use Support among Individuals Experiencing Homelessness during COVID-19. Int J Environ Res Public Health 2022, 19(6). |
| 59 | Mar KL, Mizock L, Veazey C, Nelson A: Mental healthcare barriers and facilitators experienced by homeless women with serious mental illness. Journal of Social Distress and Homelessness 2023, 32(1):59-68. |
| 60 | Eshtehardi SS, Taylor AA, Chen TA, de Dios MA, Correa-Fernandez V, Kendzor DE, Businelle MS, Reitzel LR: Sociodemographic Determinants of Nonadherence to Depression and Anxiety Medication among Individuals Experiencing Homelessness. Int J Environ Res Public Health 2021, 18(15). |
| 61 | Acorda D, Torres J, Santa Maria D: Acceptability of a Just-in-Time Adaptive Intervention for HIV Prevention among Youth Experiencing Homelessness: A Qualitative Analysis. Journal of Health Care for the Poor and Underserved 2021, 32. |
| 62 | Paradis-Gagne E, Jacques MC, Pariseau-Legault P, Ben Ahmed HE, Stroe IR: The perspectives of homeless people using the services of a mobile health clinic in relation to their health needs: a qualitative study on community-based outreach nursing. J Res Nurs 2023, 28(2):154-167. |
| 63 | DiMarco MA, Ludington SM, Menke EM: Access to and utilization of oral health care by homeless children/families. J Health Care Poor Underserved 2010, 21(2 Suppl):67-81. |
| 64 | Csikar J, Vinall-Collier K, Richemond JM, Talbot J, Serban ST, Douglas GVA: Identifying the barriers and facilitators for homeless people to achieve good oral health. Community Dent Health 2019, 36(2):137-142. |
| 65 | Mago A, MacEntee MI, Brondani M, Frankish J: Anxiety and anger of homeless people coping with dental care. Community Dent Oral Epidemiol 2018, 46(3):225-230. |
| 66 | Cairns D, Rodriguez A: A stakeholder co-design approach to designing a dental service for adults experiencing homelessness. Front Oral Health 2024, 51355429. |
| 67 | Patel EA, Shah SV, Poulson TA, Jagasia AA: An Integrative Model of ENT Healthcare for the Homeless Population. Laryngoscope 2024, 134(6):2705-2709. |
| 68 | Hwang SW, Wilkins E, Chambers C, Estrabillo E, Berends J, MacDonald A: Chronic pain among homeless persons: characteristics, treatment, and barriers to management. BMC Fam Pract 2011, 1273. |
| 69 | Bloom KC, Bednarzyk MS, Devitt DL, Renault RA, Teaman V, Van Loock DM: Barriers to prenatal care for homeless pregnant women. J Obstet Gynecol Neonatal Nurs 2004, 33(4):428-435. |
| 70 | Gordon AC, Lehane D, Burr J, Mitchell C: Influence of past trauma and health interactions on homeless women's views of perinatal care: a qualitative study. Br J Gen Pract 2019, 69(688):e760-e767. |
| 71 | Santa Maria D, Gallardo KR, Narendorf S, Petering R, Barman-Adhikari A, Flash C, Hsu HT, Shelton J, Ferguson K, Bender K: Implications for PrEP Uptake in Young Adults Experiencing Homelessness: A Mixed Methods Study. AIDS Educ Prev 2019, 31(1):63-81. |
| 72 | Gunner E, Chandan SK, Marwick S, Saunders K, Burwood S, Yahyouche A, Paudyal V: Provision and accessibility of primary healthcare services for people who are homeless: a qualitative study of patient perspectives in the UK. Br J Gen Pract 2019, 69(685):e526-e536. |
| 73 | Hamilton AB, Poza I, Hines V, Washington DL: Barriers to Psychosocial Services among Homeless Women Veterans. J Soc Work Pract Addict 2012, 12(1):52-68. |
| 74 | Johnston D, McInerney P, Thurling H: Experiences of the homeless accessing an inner-city pharmacy and medical student-run clinic in Johannesburg. Health SA 2020, 251358. |
| 75 | Saharan A, Balachander M, Sparke M: Sharing the burden of treatment navigation: social work and the experiences of unhoused women in accessing health services in Santa Cruz. Soc Work Health Care 2021, 60(8-9):581-598. |
| 76 | Dave M, Thakrar S, Bagnall H, Kumbang J: Real-time evaluation of a multi-agency TB-screening event for persons experiencing homelessness in a town with a low incidence of TB in England. Epidemiol Infect 2024, 152e73. |
| 77 | Hino P, Almeida I, Monroe A, Bertolozzi M, Taminato M, Fornari L, Rosa A: Percepção de pessoas em situação de rua sobre o tratamento da tuberculose. Medicina (Ribeirão Preto) 2022, 55. |
| 78 | Garden B, Samarina A, Stavchanskaya I, Alsterlund R, Ovregaard A, Taganova O, Shpakovskaya L, Zjemkov V, Ridell M, Larsson LO: Food incentives improve adherence to tuberculosis drug treatment among homeless patients in Russia. Scand J Caring Sci 2013, 27(1):117-122. |
| 79 | Garvin LA, Greenan MA, Edelman EJ, Slightam C, McInnes DK, Zulman DM: Increasing Use of Video Telehealth Among Veterans Experiencing Homelessness with Substance Use Disorder: Design of A Peer-Led Intervention. J Technol Behav Sci 20221-12. |

**Table S5. Overall characteristics of the included studies (n=105)**

| **Authors** | **Country** | **Study design** | **Interventions**  **(complete description)** | **N** | **Male (%)** | **Age, y (mean; SD)** | **Shelter (%)** | **Population** | **Barriers** | **Facilitators** |
| --- | --- | --- | --- | --- | --- | --- | --- | --- | --- | --- |
| Chen et al. 2016 [1] | USA | Cross-sectional | Substance use disorders treatment | 100 | 78 | 45.4; 10.9 | 100 | People with Substance use disorders (Smokers) | individual, interpersonal, and community | -- |
| Nicholls et al. 2022 [2] | USA | Qualitative | Substance use disorders treatment | 22 | 59 | 39; 11.8 | 18 | People with Substance use disorders | individual, community and policy/structural | individual, interpersonal and community |
| Rubin et al. 2021 [3] | USA | Qualitative | Substance use disorders treatment | 29 | 0 | -- | -- | People with Substance use disorders (Smokers) | individual, interpersonal, community, and institutional | interpersonal |
| Swartz et al. 2022 [4] | USA | Qualitative | Substance use disorders treatment | 29 | 48.3 | -- | -- | People with Substance use disorders | individual, interpersonal, community and institutional | individual, interpersonal and policy/structural |
| Baggett et al. 2019 [5] | USA | RCT non-blinded | Substance use disorders treatment (SmokefreeTXT + nicotine patch vs. nicotine patch) | 25 | 40 | 46.1; 9.2 | -- | People with Substance use disorders (Smokers) | community and policy/structural | -- |
| Sestito et al. 2017 [6] | USA | Qualitative | Substance use disorders treatment | 15 | 100 | 56.5; 8.4 | -- | People with Substance use disorders (Veterans) | interpersonal | interpersonal and policy/structural |
| Linnemayr et al. 2021 [7] | USA | RCT cluster cross-over | Substance use disorders treatment  (text messaging + usual care vs usual care) | 40 | -- | 22.61; 1.84 | -- | People with Substance use disorders (Smokers) | community and policy/structural | interpersonal |
| Salem et al. 2020 [8] | USA | Qualitative | Adherence model for LTBI treatment | 11 | 90.9 | 51.2; 8.60 | -- | LTBI | individual, interpersonal, community and policy/structural | individual, interpersonal, community, institutional and policy/structural |
| Chan et al. 2024 [9] | USA | Qualitative | Ambulatory intensive care | 25 | 60 | 53.2; 8.7 | -- | General | -- | interpersonal and community |
| Asgary et al. 2015 [10] | USA | Qualitative | Cancer screening | 50 | 42 | 51.7; 11.3 | 100 | General | individual | policy/structural |
| Moravac, C. C. 2018 [11] | Canada | Qualitative | Cancer screening  (breast, cervical cancer) | 26 | 0 | -- | 100 | Severe mental illness | individual, interpersonal, community, institutional and policy/structural | individual, interpersonal, and community |
| Neale et al. 2014A [12] | United Kingdom | Qualitative | CAT | 30 | 83 | 43 | 100 | People with Substance use disorders | Individual and community | individual, community and institutional |
| Neale et al. 2014B [13] | United Kingdom | Qualitative | CAT using the BFO platform | 30 | 83 | 43 | 100 | People with Substance use disorders | individual, interpersonal, community and policy/structural | interpersonal, community and policy/structural |
| Gelberg et al. 2004 [14] | USA | Qualitative | Contraception and reproductive health care | 47 | 0 | 35.6; 10.6 | 59.6 | General | individual, interpersonal, and institutional | -- |
| Eapen et al. 2023 [15] | USA | Qualitative | Contraception and reproductive health care | 81 | 53 | -- | 33.3 | General | individual, interpersonal and community | -- |
| Adedze et al. 2022 [16] | Ghana | Qualitative | Contraception and reproductive health care | 30 | 50 | -- | -- | General | individual, interpersonal, and community | -- |
| Kennedy et al. 2014 [17] | USA | Qualitative | Contraception and reproductive health care | 22 | 0 | 32 | 100 | General | individual, interpersonal, and community | community |
| Kachingwe et al. 2019 [18] | USA | Qualitative | Contraception and reproductive health care | 11 | 0 | 15.6; 1.29 | -- | General | individual | individual, interpersonal, community and policy/structural |
| Vickery et al. 2023 [19] | USA | Qualitative and open trial | D-HOMES program | 10 | 60 | 57.4; 4.1 | -- | Type 2 diabetes | individual, interpersonal, community, institutional, and policy/structural | individual, interpersonal and institutional |
| Weber et al. 2019 [20] | USA | Qualitative | Emergency care | 34 | 100 | 56 | -- | Veterans | individual and interpersonal | individual |
| McCallum et al. 2020 [21] | Canada | Qualitative | Emergency care | 16 | 43.8 | 45 | 81.25 | General | individual, interpersonal, and institutional | interpersonal |
| Chinchilla et al. 2024 [22] | USA | Qualitative | Emergency care | 16 | 81.2 | -- | -- | Veterans | individual, community and institutional | individual, interpersonal, community and institutional |
| Moczygemba et al. 2021 [23] | USA | pre-post | GPS-mHealth vs emergency care | 30 | 67 | 44.1; 9.7 | 7 | Chronic conditions | individual, interpersonal, and policy/structural | individual and interpersonal |
| Partida et al. 2022 [24] | USA | Prospective cohort | HCV education/care | 155 | 74.8 | 56.1 | 100 | Hepatitis C | individual | individual |
| Masson et al. 2020 [25] | USA | Qualitative | HCV testing and treatment | 20 | 50 | -- | -- | HCV | individual, interpersonal, community and institutional | individual, interpersonal, community and policy/structural |
| Radó et al. 2024 [26] | Hungary | Qualitative | Healthcare | 10 | 60 | -- | 100 | General | individual and policy/structural | Policy/structural |
| Oliveira et al. 2021 [27] | Brazil | Qualitative | Healthcare | 10 | 90 | -- | 0 | General | individual, interpersonal, institutional and policy/structural | -- |
| Christiani et al. 2008 [28] | USA | Qualitative | Healthcare | 54 | 68.5 | 20.5 | -- | People with Substance use disorders | individual, interpersonal, community and institutional | interpersonal, community, institutional and policy/structural |
| Ensign et al. 2002 [29] | USA | Qualitative | Healthcare | 20 | 0 | 18.2 | -- | General | individual, interpersonal, community, institutional and policy/structural | interpersonal |
| Darbyshire et al. 2006 [30] | Australia | Qualitative | Healthcare | 10 | 30 | -- | -- | Mental illness | individual, interpersonal, community and institutional | interpersonal |
| Henderson, et al. 2022 [31] | USA | Qualitative | Healthcare | 16 | 100 | -- | 100 | General | individual, interpersonal, community and institutional | individual, community and policy/structural |
| Ensign et al. 1998 [32] | USA | Qualitative | Healthcare | 31 | 35.5 | -- | 100 | General | interpersonal, community, institutional and policy/structural | -- |
| Lewis et al. 2019 [33] | USA | Cross-sectional | Healthcare | 974 | 0 | -- | 67 | General | individual, interpersonal, community and institutional | individual, interpersonal, community and policy/structural |
| Ramsay et al. 2019 [34] | Canada | Qualitative | Healthcare | 16 | 81.3 | 42.9 | -- | General | individual, interpersonal, community and institutional | interpersonal, community, institutional and policy/structural |
| Hudson et al. 2010 [35] | USA | Qualitative | Healthcare | 24 | 75 | -- | -- | General | interpersonal, community, institutional and policy/structural | -- |
| Nickasch et al. 2009 [36] | USA | Qualitative | Healthcare | 9 | 44.4 | 40.5 | -- | General | interpersonal, community and policy/structural | -- |
| Mnkandla et al. 2023 [37] | South Africa | Qualitative | Healthcare | 25 | -- | 32 | -- | People with Substance use disorders | interpersonal | interpersonal and community |
| Mc Conalogue et al. 2021 [38] | United Kingdom | Qualitative | Healthcare | 28 | 71 | -- | 54 | General | Interpersonal and policy/structural | Institutional and policy/structural |
| Prado et al. 2021 [39] | Brazil | Qualitative | Healthcare | 10 | 70 | -- | -- | General | interpersonal | -- |
| Wen et al. 2007 [40] | Canada | Qualitative | Healthcare | 17 | 76.5 | 40 | 100 | General | interpersonal | interpersonal |
| Woith et al. 2017 [41] | USA | Qualitative | Healthcare | 15 | 66 | 34.3 | -- | General | interpersonal, community and policy/structural | interpersonal |
| Haley et al. 2007 [42] | USA | Qualitative | Healthcare | 49 | 79.6 | 41 | 0 | General | interpersonal, and policy/structural | Interpersonal, institutional and policy/structural |
| Eapen et al. 2022 [43] | USA | Qualitative | Healthcare | 81 | 53 | -- | -- | Pregnancy | individual, community, institutional and policy/structural | -- |
| Kneck et al. 2021 [44] | Sweden | Qualitative | Healthcare | 26 | 0 | 46.6; 11.2 | -- | General | individual, interpersonal, and policy/structural | interpersonal |
| McGonigle et al. 2018 [45] | USA | Cohort prospective | Healthcare | 509 | 91.2 | 42.7 | 100 | General | individual, interpersonal, and community | -- |
| Ecthin et al. 2021 [46] | USA | Qualitative | Healthcare | 21 | 100 | -- | -- | Veterans | Interpersonal, community, and institutional | Individual, interpersonal, and policy/structural |
| Bell et al. 2024 [47] | United Kingdom | Qualitative | Nurse-led outreach Healthcare | 18 | 72.2 | -- | -- | General | -- | interpersonal, community and policy/structural |
| Ramirez et al. 2022 [48] | USA | Qualitative | Healthcare (shelter clinic site vs based clinic site) | 31 | 75/80 | 59/49 | -- | General | individual, community and institutional | individual, interpersonal and institutional |
| Doroshenko et al. 2012 [49] | Canada | Qualitative | Immunization | 29 | 55.2 | -- | 100 | General | individual, interpersonal, and community | -- |
| Jenkinson et al. 2024 [50] | Canada | Qualitative | Immunization (COVID) | 31 | 55 | -- | -- | General | individual | -- |
| Grune et al. 2023 [51] | Germany | Qualitative | Immunization (COVID) | 20 | 75 | 55 | 100 | General | Individual, interpersonal and policy/structural | individual, interpersonal, and institutional |
| Coe et al. 2015 [52] | USA | Retrospective cohort | Medication | 426 | 48.4 | 44.7 | -- | General | individual, community, Institutional and policy/structural | -- |
| Richler et al. 2019 [53] | Canada | Qualitative | Medication | 12 | 83.3 | 48.5 | 66.7 | General | individual | individual and community |
| Nyamathi et al. 1989 [54] | USA | Retrospective cohort | Medication | 61 | 100 | 39.2 | -- | General | individual and community | individual, interpersonal, and policy/structural |
| Paudyal et al. 2017 [55] | United Kingdom | Qualitative | Medications | 25 | 60 | 40.7 | -- | General | individual, community, institutional and policy/structural | individual |
| Adkins et al. 2017 [56] | USA | Qualitative | Mental health services | 24 | 37.5 | 18.8; 0.8 | 100 | General | interpersonal | interpersonal |
| Guenzel et al. 2020 [57] | USA | Qualitative and quantitative | Mental health services | 127 | -- | 41.7 | 100 | General | individual, interpersonal, community and institutional | -- |
| Adams et al. 2022 [58] | United Kingdom | Qualitative | Mental health services | 26 | 61.5 | 40.7 | -- | General | individual, community, institutional and policy/structural | individual |
| La Mar et al. 2023 [59] | USA | Qualitative | Mental health services | 13 | 0 | 38 | 100 | Serious mental illness | Individual | Individual, interpersonal, and policy/structural |
| Eshtehardi et al. 2021 [60] | USA | Cross-sectional | Mental health (depression vs anxiety medication) | 381 | 58 | 44; 12.08/43.5; 1.02 | 0 | Depression and anxiety | individual and community | -- |
| Acorda et al. 2021 [61] | USA | Qualitative | MY- RID app for HIV prevention | 16 | 50 | 20 | -- | General | community and policy/structural | community |
| Paradis-Gagné et al. 2023 [62] | Canada | Qualitative | Nurse-led mobile clinic vs usual care | 12 | 83.3 | -- | -- | General | community and institutional | interpersonal, community and institutional |
| DiMarco et al. 2010 [63] | USA | Quasi-experimental | Oral Health Care | 120 | 0 | 30 | 100 | General | individual, community and policy/structural | -- |
| Csikar et al. 2019 [64] | United Kingdom | Qualitative | Oral health care | 16 | 87.5 | -- | -- | General | individual, interpersonal, community and institutional | individual, interpersonal, institutional and policy/structural |
| Mago et al. 2018 [65] | Canada | Qualitative | Oral health care | 25 | 72 | 51 | -- | General | individual, interpersonal, community and institutional | -- |
| Cairns, D et al. 2024 [66] | United Kingdom | Qualitative | Oral health care | 12 | -- | -- | -- | General | individual, interpersonal, and policy/structural | -- |
| Patel et al. 2024 [67] | USA | Retrospective cohort | Otolaryngology care | 93 | -- | -- | -- | Cancer | community | community |
| Hwang et al. 2011 [68] | Canada | Cross-sectional | Pain management | 152 | 79.6 | -- | 100 | Chronic pain | individual, interpersonal, community, institutional and policy/structural | -- |
| Bloom et al. 2004 [69] | USA | Qualitative | Prenatal care | 47 | 0 | 24.96;6.14 | -- | Pregnancy | individual, interpersonal, community, institutional and policy/structural | -- |
| Gordon et al. 2019 [70] | USA | Qualitative | Prenatal care | 11 | 0 | -- | -- | Pregnancy | interpersonal | interpersonal |
| Santa Maria et al. 2019 [71] | USA | Qualitative and quantitative | PrEP | 45 | 40.9 | 20.7; 2.1 | -- | General | individual, interpersonal, community and policy/structural | individual, interpersonal, community and policy/structural |
| Gunner et al. 2019 [72] | United Kingdom | Qualitative | Primary healthcare | 22 | 68 | -- | 63.6 | General | individual, interpersonal, community, institutional and policy/structural | individual, interpersonal, community and policy/structural |
| Hamilton, et al. 2012 [73] | USA | Qualitative | Psychosocial services | 29 | 0 | 48 | 47 | Veterans | individual, interpersonal, community and policy/structural | -- |
| Johnston et al. 2020 [74] | South Africa | Qualitative | THS | 18 | 83.3 | -- | 100 | General | individual, interpersonal, and policy/structural | individual and policy/structural |
| Saharan et al. 2021 [75] | USA | Qualitative | Treatment navigation | 5 | 0 | -- | -- | General | individual, interpersonal, and community | community |
| Dave et al. 2024 [76] | United Kingdom | Qualitative | Tuberculosis-screening | 28 | 64 | 42 | 50 | Tuberculosis | individual and interpersonal | interpersonal |
| Hino et al. 2022 [77] | Brazil | Qualitative | Tuberculosis treatment | 24 | 91.6 | -- | 0 | Tuberculosis | individual, interpersonal, institutional and policy/structural | interpersonal |
| Gärden et al. 2013 [78] | Russia | Prospective cohort | Tuberculosis treatment + food incentives vs tuberculosis treatment | 142 | 94 | 48.6 | -- | Tuberculosis | -- | policy/structural |
| Garvin et al. 2022 [79] | USA | Qualitative | Video telehealth | 210 | -- | -- | -- | People with Substance use disorders (veterans) | individual, interpersonal, community, and policy/structural | interpersonal and community |

*hostels; BFO: Breaking Free Online; CAT: Computer-Assisted Therapy; HAAT: Highly Active Antiretroviral Therapy; H-PACT: Homeless-Patient Aligned Care Teams; MAT: Maximally Assisted Therapy; MMT: Methadone maintenance treatment; NCMI: Nurse Case Management with Incentives; PrEP: pre-exposure prevention; RCT: Randomized clinical trials; THS Trinity Healthcare; USA: United States of America

**TABLE S6. Barriers and facilitators reported by the included studies**

| **Authors** | **Interventions (summarized)** | **Population** | **Individual Level Barriers** | **Interpersonal Level**  **Barriers** | **Community Level Barriers** | **Institutional level barriers** | **Policy/**  **Structural Level Barriers** | **Individual Level Facilitators** | **Interpersonal Level Facilitators** | **Community Level Facilitators** | **Policy/**  **Structural Level Facilitators** | **Institutional level facilitators** |
| --- | --- | --- | --- | --- | --- | --- | --- | --- | --- | --- | --- | --- |
| Chen et al. 2016 [1] | Substance use disorders treatment | People with Substance use disorders (Smokers) | - Abstinence - Treatment-related beliefs/concerns (safety) | - Peer pressure | - Access to services | -- | -- | -- | -- | -- | -- | -- |
| Nicholls et al. 2022 [2] | Substance use disorders treatment | People with Substance use disorders | - Treatment-related beliefs/concerns (safety) | -- | - Payment - Insurance | -- | - Resources | - Knowledge on treatment | - Peer and professional support - Access to treatment | - Access to social services | -- | -- |
| Rubin et al. 2021 [3] | Substance use disorders treatment | People with Substance use disorders (Smokers) | - Cognitive, behavioral and health challenges (chronic condition) - Treatment-related beliefs/concerns (efficacy) | - Distrust of peers - Fear of negative influence | - Transport - Access to other locations | - Same health professional - Time | -- | -- | - Peer and professional relationships - Empathetic attitude | -- | -- | -- |
| Swartz et al. 2022 [4] | Substance use disorders treatment | People with Substance use disorders | - Emotional challenges - No fear of dying - Treatment-related beliefs/ concerns (efficacy) | - Unsympathetic attitudes - Negligence - Stigma - Living in high-use environment | - No opening clinics - Incarceration | - Restrictions on take-home doses | -- | - Fear of death after overdose - Knowledge on treatment - Insurance | - Peer and professional support | -- | - Care located on-site |  |
| Sestito et al. 2017 [6] | Substance use disorders treatment | People with Substance use disorders (Veterans) | -- | - Family and friend support - Professionals’ relationship | -- | -- | -- | -- | - Building trust - Support network | -- | - Care located on-site | -- |
| Linnemayr et al. 2021 [7] | Substance use disorders treatment | People with Substance use disorders (Smokers) | -- | -- | - Phone charging access | -- | - Security | -- | - Language | -- | -- |  |
| Salem et al. 2020 [8] | Adherence model for LTBI treatment | LTBI | - Use of illicit substances - Healthcare services and institutions beliefs/concerns - Treatment-related beliefs/concerns (safety) - Self-care | - Distrust of peers - Language - Stigma | - Access to services - Unstable situation | -- | - Incentives - Time needed for intervention - Knowledge on treatment | - Knowledge on treatment | - Communication with health professionals - Peer and professional support - Comprehensive approach | - Content of the intervention | - Service location | - Care integration |
| Chan et al. 2024 [9] | Ambulatory Intensive Care | General | -- | -- | -- | -- | -- | -- | - Communication with health professionals - Peer and professional support - Destigmatized attitude - Respectful approach - Nonjudgmental attitudes | - Access to services | -- | -- |
| Asgary et al. 2015 [10] | Cancer screening | General | - Treatment-related beliefs/concerns (safety) - Fear of bad news - Cognitive, behavioral, and health challenges (embarrassment) | -- | -- | -- | -- | -- | -- | -- | - Shelter | -- |
| Moravac, C. C. 2018 [11] | Cancer screening | Severe mental illness | - Healthcare services and institutions beliefs/concerns | - Discrimination - Disinterested attitudes | - Access to physicians - Transport | - Navigate the system | - Skills to fill in documents - Personal document | - Experience with the service - Knowledge on treatment | - Support network | - Access to services | -- |  |
| Neale et al. 2014A [12] | CAT | People with Substance use disorders | - Digital literacy | -- | - Access to computers - Internet | -- | -- | - Digital literacy | -- | - User-friendly interface | -- | - Flexibility |
| Neale et al. 2014B [13] | CAT using the BFO platform | People with Substance use disorders | - Emotions - Digital literacy | - Mentor absence | - Privacy - Access to computers |  | -- | -- | - Peer and professional support | - Convenience - User-friendly interface - Virtual care | - Accessibility - Flexibility - No forms - No waiting list |  |
| Gelberg et al. 2004 [14] | Contraception | General | - Lack of prioritization of health due to survival needs - Knowledge on treatment/disease | - Partner' influence - Disrespectful attitudes - Stigma | -- | - Waiting times - Bureaucracy - Same health professional - Restrictions on doses | -- | -- | -- | -- | -- |  |
| Eapen et al. 2023 [15] | Contraception | General | - Treatment-related beliefs/concerns (efficacy/safety) - Adherence - Cognitive, behavioral, and health challenges (Lifestyle/habits) - Knowledge on treatment/disease | - Partner' influence - Stigma | - Access to medication and devices | -- | -- | -- | -- | -- | -- | -- |
| Adedze et al. 2022 [16] | Contraception | General | - Personal beliefs (religious) | - Providers’ attitude | - Payment | -- | -- | -- | -- | -- | -- | -- |
| Kennedy et al. 2014 [17] | Contraception | General | - Treatment-related beliefs/concerns (safety) - Fear of bad news - Lack prioritization of health due to survival needs | - Partner' influence - Distinguish treatment | - Store the medications | -- | -- | -- | -- | - Free medication | -- | -- |
| Kachingwe et al. 2019 [18] | Contraception | General | - Cognitive, behavioral, and health challenges (fear of devices and procedures) - Fear from previous experiences - Knowledge on treatment | -- | -- | -- | -- | - Sense of autonomy - Access to basic needs | - Listened by the professionals - Building trust | - Low-maintenance treatments | - Access to social services - Incentive (cell phone) | -- |
| Vickery et al. 2023 [19] | D-HOMES program | Type 2 diabetes | - Cognitive, behavioral, and health challenges (mental health) - Trauma history - Use of illicit substances | - Trust in providers - Stigma | - Tailored interventions - Unstable situation | - Health system navigation | - Research inclusion | - Motivation to improve self-care | - Personal characteristics | -- | -- | Integration between support institutions and health services |
| Weber et al. 2019 [20] | Emergency care | General | - Fear from previous experiences - Healthcare services and institutions beliefs/concerns | - Trust in providers - No space to expression | -- | -- | -- | - Feelings of value and self-worth | -- | -- | -- | -- |
| McCallum et al. 2020 [21] | Emergency care | General | - Use of illicit substances - Fear from previous experiences | - Discrimination - Stigma | -- | - Waiting times | -- | -- | - Compassion for professionals | -- | -- | -- |
| Chinchilla et al. 2024 [22] | Emergency care | Veterans | - Lack prioritization of health due to survival needs - Cognitive, behavioral, and health challenges (physical) - Healthcare services and institutions beliefs/concerns | -- | - Transport | - Appointments | -- | - Access to basic needs | - Communication with health professionals - Peer and professional support | - Virtual services | -- | - Appointment reminders |
| Moczygemba et al. 2021 [23] | GPS-mHealth vs emergency care | Chronic conditions | - Digital literacy | - Prejudice | -- | -- | - Security | - Well-being | - Support network - self-management support | -- | -- | -- |
| Partida et al. 2022 [24] | HCV care | Hepatitis C | - Use of illicit substances - Alcohol abuse - Self-blame | -- | -- | -- | -- | - Knowledge on healthcare | -- | -- | -- | -- |
| Masson et al. 2020 [25] | HCV testing and treatment | HCV | - Healthcare services and institutions beliefs/concerns - Use of illicit substances - Cognitive, behavioral, and health challenges (chronic condition) - Knowledge on treatment | - Trust in providers - Stigma | -- | - Bureaucracy | -- | - Prior experiences with services - Healthcare knowledge | - Prevent transmission to others | - Access to treatment | - Financial incentives | -- |
| Radó et al. 2024 [26] | Health technology | General | - Lack of interest - Fear from previous experiences - Fear of making mistakes - Trust in the service | -- | -- | -- | - Lack of security | -- | -- | -- | Free Wi-Fi | -- |
| Oliveira et al. 2021 [27] | Healthcare | General | - Use of illicit substances | - Lack empathy - Stigma - Prejudice - Discrimination | -- | - Institutional requirements - Response delays | - Lack proximity to services | -- | -- | -- | -- | -- |
| Christiani et al. 2008 [28] | Healthcare | People with Substance use disorders | - Use of illicit substances - Frustration with health services | - Discrimination; - Uncomfortable around peers | - Payment - Appropriate services | - Appointments - Bureaucracy | -- | -- | - Peer and professional support - Nonjudgemental approach | - Culturally competent services | - Free services | - Timely services |
| Ensign et al. 2002 [29] | Healthcare | General | - Cognitive, behavioral, and health challenges (physical) | - Lack of support - Aggressive attitudes - Language - Trust in providers | - Payment - Transport - Insurance | - Too many forms | - Personal document | -- | - Relationship with healthcare professionals - Nonjudgemental approach - Personal characteristics | -- | -- |  |
| Darbyshire et al. 2006 [30] | Healthcare | Mental illness | - Personal control - Resources for basic needs (food) | - Stigma - Space for patients to expression - Explanations and information - Busy providers | - Transport | - Coordination between services | -- | -- | - Nonjudgemental approach - Labelling identity - Welcoming attitude, trust | -- | -- |  |
| Henderson, et al. 2022 [31] | Healthcare | General | - Fear from previous experiences - Knowledge on treatment | - Providers negative attitudes - Disrespectful attitudes - Stereotypes | - Transport - Insurance | - Appointment - Coordination between services - Wanting specific providers | -- | - Personal characteristics | -- | - Personalized services | -- | -- |
| Ensign et al. 1998 [32] | Healthcare | General | -- | - Judgment - Parental consent | - Payment - Insurance | - Waiting times | - Cleanliness in health facilities | -- | -- | -- | -- | -- |
| Lewis et al. 2019 [33] | Healthcare | General | - Cognitive, behavioral, and health challenges (too sick) - Lack of prioritization of health due to survival needs - Healthcare services and institutions beliefs/concerns - Fear of bad news | - Fear of judgment - Personal characteristics of providers | - Payment - Transport | - Waiting times - Same health professional | -- | - Housing conditions | - Help from shelters and soup kitchens | - Transportation support | - Centralized services - Care located on-site | -- |
| Ramsay et al. 2019 [34] | Healthcare | General | - Negative experiences with medications | - Empathy - Trust in providers | - Payment - Transport - Access to services - Intervention content | - Waiting times   Coordination | -- | -- | - Relationship with healthcare professionals | - Transportation support | - Centralized services | - Integration between services - Continuity of care |
| Hudson et al. 2010 [35] | Healthcare | General | -- | - Discrimination - Stigma - Judgment | - Insurance - Access to services | - Waiting times - Bureaucracy | - No free services | -- | -- | -- | -- | -- |
| Nickasch et al. 2009 [36] | Healthcare | General | -- | - Lack compassion of providers - Stereotypes - Presumptions | - Payment - Transport - Insurance - Physical needs | -- | - Available resources | -- | -- | -- | -- | -- |
| Mnkandla et al. 2023 [37] | Healthcare | People with Substance use disorders | - Fear of bad news | - Negligence - Relationship with professionals - Stigma | -- | -- | -- | -- | - Empathetic attitude - Personal characteristics - Comprehensive approach | - Mobile strategies | -- | -- |
| Mc Conalogue et al. 2021 [38] | Healthcare | General | -- | - Judgment | -- | -- | - Transition/unstable situation | -- | -- | -- | - Location | - Waiting time |
| Prado et al. 2021 [39] | Healthcare | General | -- | - Stigma - Discrimination - Aggressive attitudes - Distinguish treatment | -- | -- | -- | -- | -- | -- | -- | -- |
| Wen et al. 2007 [40] | Healthcare | General | -- | - Discrimination - Dehumanized attitudes - Explanations and information - Trust in providers | -- | -- | -- | -- | - Listened by the professionals - Comprehensive approach - Communication with health professionals | -- | -- | -- |
| Woith et al. 2017 [41] | Healthcare | General | -- | - Listened by the professionals - Empathy - Compassion - Disinterested attitudes | - Payment | -- | - Unstable situation | -- | - Attitude of civility - Professional characteristics - Empathetic attitude | -- | -- | -- |
| Haley et al. 2007 [42] | Healthcare | General | -- | - Stigma | -- | -- | - Knowledge on treatment | -- | - Building trust | -- | - Accessible location | - Time |
| Eapen et al. 2022 [43] | Healthcare | Pregnancy | - Knowledge on treatment | -- | - Payment - Transport - Insurance | - Eligibility | - Unstable situation | -- | -- | -- | -- | -- |
| Kneck et al. 2021 [44] | Healthcare | General | - Lack prioritization of health due to survival need - Treatment-related beliefs/concerns (safety) - Use of illicit substances - Cognitive, behavioral, and health challenges (physical, mental) | - Trust in providers - Negative perception of professionals - Judgment | -- |  | - Same health professional | -- | - Relationship with healthcare professionals - Respect | -- | -- |  |
| McGonigle et al. 2018 [45] | Healthcare | General | - Incarceration - Use of illicit substances - Alcohol abuse | - Trust in providers | - Payment - Transport | -- | -- | -- | -- | -- | -- | -- |
| Ramirez et al. 2022 [48] | Healthcare | General | - Cognitive, behavioral, and health challenges (physical, mental) | -- | - Transport - Costs | - Appointment | -- | - Willingness to recover - Knowledge on treatment | - Listened by the professionals - Peer and professional support - Learning support | -- | -- | - Care integration |
| Ecthin et al. 2021 [46] | Healthcare | Veterans | -- | - Disinterested attitudes - Lack of communication - Judgement | - Transport | - Bureaucracy - Appointment - Services Time - Treatment with multiples providers | -- | - Providers attitudes | - providers to be worthy - Same provriders on all treatment - Good communication with providers | -- | - monetary benefit | -- |
| Doroshenko et al. 2012 [49] | Immunization | General | - Personal beliefs - Healthcare services and institutions beliefs/concerns - Knowledge on treatment | - Explanations and information | - Access to services - Payment - Information on services - Vaccination policy | -- | -- | -- | -- | -- | -- | -- |
| Jenkinson et al. 2024 [50] | Immunization (COVID) | General | - Treatment-related beliefs/concerns (efficacy, safety) | -- | -- | -- | -- | -- | -- | -- | -- | -- |
| Grune et al. 2023 [51] | Immunization (COVID) | General | - Treatment-related beliefs/concerns (safety) - Healthcare services and institutions beliefs/concerns | - Language | -- | -- | - Personal document | - Perceptions about safety and efficacy - Familiarity with intervention - High-risk perception of the condition | - Protecting others from transmission - Facilities support for PEH | -- | -- | - De-bureaucratization |
| Coe et al. 2015 [52] | Medication | General | - Treatment-related beliefs/concerns (efficacy, safety) - Forgetting the appointment - Self-management - Cognitive, behavioral, and health challenges (stress) | -- | - Access to medication and devices - Regimen complexity - Transport - Payment | - Coordination between services | - Thefts - Unstable situation | -- | -- | -- | -- | -- |
| Richler et al. 2019 [53] | Medication | General | - Treatment-related beliefs/concerns (need, safety) | -- | -- | -- | -- | - Medication education | -- | - Hospital services | -- | -- |
| Nyamathi et al. 1989 [54] | Medication | General | - Forgetting to take medication - Treatment-related beliefs/concerns (need) - Resources for basic needs (water) | -- | - Privacy - Medication storage - Access to medication and devices - Payment | -- | -- | - Not carrying medication - Knowledge on how to take medications - Religions | - support network; staying away from people who drink | -- | - Accessible location - Proximity location | -- |
| Paudyal et al. 2017 [55] | Medications | General | - Unstable situation - Resources for basic needs - Violent past | -- | - Medication storage | - Requirement to withdraw the drug on time | - Unstable situation | - Prescribed medicines - Beliefs about consequences of non-adherence | -- | -- | -- | -- |
| Adkins et al. 2017 [56] | Mental health | General | -- | - Relationship with professionals - Disinterested attitudes | -- | -- | -- | -- | - Relationship with professionals | -- | -- | -- |
| Guenzel et al. 2020 [57] | Mental health | General | - Treatment-related beliefs/concerns (efficacy, safety) - History of misdiagnosis - Knowledge on treatment | - Provider empathy - Stigma | - Payment - Transport - Access to services - Insurance | - Waiting time | -- | -- | -- | -- | -- | -- |
| Adams et al. 2022 [58] | Mental health | General | - Lack prioritization of health | -- | - Access to services | - Disjointed care - Coordination between services | - Unstable situation | - Knowledge about the services | -- | -- | -- | -- |
| Eshtehardi et al. 2021 [60] | Mental health | Depression and anxiety | - Treatment-related beliefs and concerns (efficacy, safety, needs) - Forgetting to take the medication | -- | - Access to medication - Insurance | -- | -- | -- | -- | -- | -- | -- |
| La Mar et al. 2023 [59] | Mental health | Seriou mental illness | - personal beliefs (feelings of brokenness) - beliefs about healthcare services - Negative perception of professionals | -- | -- | -- | -- | - feelings of love and trust in oneself | - feelings of welcome | -- | - centralized healthcare services | -- |
| Acorda et al. 2021 [61] | MY- RID app for HIV prevention | General | -- | -- | - Intervention structure and content | -- | - Thefts | -- | -- | - User-friendly interface | -- | -- |
| Paradis-Gagné et al. 2023 [62] | Nurse-led mobile clinic | General | -- | -- | - Payment - Transport - Insurance | - Bureaucracy - Waiting times - Continuity and integration between services | -- | -- | - Peer and professional support - Listened by the professionals - Building trust - Peer and professional support - Holistic approach | - Social visibility of clinic - Access to medications | -- | - De-bureaucratization - Time |
| Bell et al. 2024 [47] | Nurse-led outreach healthcare | General | -- | -- | -- | -- | -- | -- | - Support with medication - Friendly approach | - Access to medications | - Care located on-site | -- |
| DiMarco et al. 2010 [63] | Oral health care | General | - Use of licit substances - Health-related beliefs and concerns - Cognitive, behavioral, and health challenges (victimization) | -- | - Insurance | -- | -- | -- | -- | -- | -- | -- |
| Csikar et al. 2019 [64] | Oral health care | General | - Healthcare services and institutions beliefs/concerns - Lack prioritization of health due to survival needs | - Prejudice - Stigma - Disrespectful attitudes - Judgment | - Payment | - Bureaucracy | -- | - Knowledge on healthcare | - Destigmatized attitude | -- | - Accessible location - Proximity location | - Single appointment |
| Mago et al. 2018 [65] | Oral health care | General | - Cognitive, behavioral, and health challenges (anxiety) - Fear from previous experiences | - Disrespectful attitudes - Stigma | - Payment - Information on services | - Appointments | -- | -- | -- | -- | -- | -- |
| Cairns, D et al. 2024 [66] | Oral health care | General | - Use of illicit substances - Resources for basic needs - Fear of the devices and procedures - Knowledge on treatment | - Trust in providers - Negative perception of professionals - Judgment | -- | -- | - Unstable situation | -- | -- | -- | -- | -- |
| Patel et al. 2024 [67] | Otolaryngology care | Cancer | -- | -- | - Transport | -- | -- | -- | -- | - Virtual services - Transportation support | -- | -- |
| Hwang et al. 2011 [68] | Pain management | Chronic pain | - Treatment-related beliefs/concerns (efficacy, safety) - Cognitive, behavioral, and health challenges (physical, mental) - Use of illicit substances - Stress - Self-management | - Negligence - Relationship with professionals - Support | - Transport - Payment - Access to services - Access to medications | - Appointment - Waiting time | - Personal document - Resources for basic needs (sleep) | -- | -- | -- | -- | -- |
| Bloom et al. 2004 [69] | Prenatal care | Pregnancy | - Fear from previous experiences | - Relationship with professionals | - Transport - Payment - Travel time | - Waiting times - Appointments | - Proximity to services | -- | -- | -- | -- | -- |
| Gordon et al. 2019 [70] | Prenatal care | Pregnancy | -- | - Stigma - Trust in providers | -- | -- | -- | -- | - Building trust | -- | -- | -- |
| Santa Maria et al. 2019 [71] | PrEP | General | - Low perceived risk - Treatment-related beliefs/concerns (efficacy) - Forgetting to take the medication - Knowledge on treatment | - Stigma - Fear of judgment | - Privacy - Medication storage - Insurance - Payment | -- | - Unstable situation | - Knowledge on treatment | - Empathy - Support for medication use - Social networks - Peer and professional support - Group-based adherence | - Low-maintenance treatments - Free medication and service | - Care located on-site | -- |
| Gunner et al. 2019 [72] | Primary Healthcare | General | - Healthcare services and institutions beliefs/concerns - Cognitive, behavioral, and health challenges (embarrassment) | - Discrimination - Stigma - Trust in providers | - Access services - Access to medications | - Navigate the system - Eligibility - Bureaucracy - Coordination between services | - Personal document - Unstable situation | - Beliefs on the consequences of not receiving health care - Knowledge on services - Integration with rules | - Proactive attitude - Professionals’ knowledge - Social network - Building trust - Family/friends support - Facilities support | - Access to services - Signposting to appropriate services | - Care located on-site - Location near hostels | -- |
| Hamilton, et al. 2012 [73] | Psychosocial Services | General | -- | -- | - Access services - Information on services |  | - Coordination between services | -- | -- | -- | -- |  |
| Johnston et al. 2020 [74] | THS | General | - Fear from previous experiences | - Stigma - Discrimination | -- |  | - Security | -- | - Respectful approach - Compassion - Professionalism | -- | - Expanding the services |  |
| Saharan et al. 2021 [75] | Treatment navigation | General | - Use of illicit substances | - Disregard and exclusion - Distrust in peers - Navigation assistance | - Insurance |  | -- | -- | -- | - Nontraditional healthcare - Alternative treatments | -- |  |
| Dave et al. 2024 [76] | TB-screening | General | - No trust in health technology | - Stigma - Language | -- |  | -- | -- | - Individuals as translators | -- | -- |  |
| Hino et al. 2022 [77] | Tuberculosis treatment | Tuberculosis | - Use of illicit substances - Treatment-related beliefs/concerns (safety) | - Dialogue - Prejudice - Disrespect - Negligence | -- | - Same health professional | - Unstable situation | -- | - Family/friends support - Peer and professional support - Relationship with professionals | -- | -- | -- |
| Gärden et al. 2013 [78] | Tuberculosis treatment + food incentives | Tuberculosis | -- | -- | -- | -- | -- | -- | -- | -- | - Food incentives | -- |
| Garvin et al. 2022 [79] | Video Telehealth | People with Substance use disorders (veterans) | - Cognitive, behavioral, and health challenges (cognitive) - Digital literacy | - Overall support | - Privacy - Access to computers and phones | -- | - Internet | -- | - Training on technology use | - Virtual care | -- | -- |

BFO: Breaking Free Online; CAT: Computer-Assisted Therapy; HAAT: Highly Active Antiretroviral Therapy; H-PACT: Homeless-Patient Aligned Care Teams; MAT: Maximally Assisted Therapy; MMT: Methadone maintenance treatment; NCMI: Nurse Case Management with Incentives; PrEP: pre-exposure prevention; RCT: Randomized clinical trials; THS Trinity Healthcare; USA: United States of America

**References**

1. Chen JS, Nguyen AH, Malesker MA, Morrow LE: High-Risk Smoking Behaviors and Barriers to Smoking Cessation Among Homeless Individuals. Respir Care 2016, 61(5):640-645.

2. Nicholls MJ, Urada LA: Homelessness and polysubstance use: A qualitative study on recovery and treatment access solutions around an urban library in Southern California, USA. Health Soc Care Community 2022, 30(1):e175-e183.

3. Rubin SB, Vijayaraghavan M, Weiser SD, Tsoh JY, Cohee A, Delucchi K, Riley ED: Homeless women's perspectives on smoking and smoking cessation programs: A qualitative study. Int J Drug Policy 2021, 98103377.

4. Swartz N, Adnan T, Perea F, Baggett TP, Chatterjee A: "Sick and tired of being sick and tired": Exploring initiation of medications for opioid use disorder among people experiencing homelessness. J Subst Abuse Treat 2022, 138108752.

5. Baggett TP, McGlave C, Kruse GR, Yaqubi A, Chang Y, Rigotti NA: SmokefreeTXT for Homeless Smokers: Pilot Randomized Controlled Trial. JMIR Mhealth Uhealth 2019, 7(6):e13162.

6. Sestito SF, Rodriguez KL, Saba SK, Conley JW, Mitchell MA, Gordon AJ: Homeless veterans' experiences with substance use, recovery, and treatment through photo elicitation. Subst Abus 2017, 38(4):422-431.

7. Linnemayr S, Zutshi R, Shadel W, Pedersen E, DeYoreo M, Tucker J: Text Messaging Intervention for Young Smokers Experiencing Homelessness: Lessons Learned From a Randomized Controlled Trial. JMIR Mhealth Uhealth 2021, 9(4):e23989.

8. Salem BE, Klansek E, Morisky DE, Shin SS, Yadav K, Chang AH, Nyamathi AM: Acceptability and Feasibility of a Nurse-Led, Community Health Worker Partnered Latent Tuberculosis Medication Adherence Model for Homeless Adults. Int J Environ Res Public Health 2020, 17(22).

9. Chan B, Hulen E, Edwards ST, Geduldig A, Devoe M, Nicolaidis C, Korthuis PT, Saha S: Perceptions of Medically Complex Patients Enrolled in an Ambulatory Intensive Care Unit at a Healthcare-for-the-Homeless Clinic. J Am Board Fam Med 2025, 37(5):888-899.

10. Asgary R, Sckell B, Alcabes A, Naderi R, Ogedegbe G: Perspectives of cancer and cancer screening among homeless adults of New York City shelter-based clinics: a qualitative approach. Cancer Causes Control 2015, 26(10):1429-1438.

11. Moravac CC: Reflections of Homeless Women and Women with Mental Health Challenges on Breast and Cervical Cancer Screening Decisions: Power, Trust, and Communication with Care Providers. Front Public Health 2018, 630.

12. Neale J, Stevenson C: Positive and negative features of a computer assisted drug treatment program delivered by mentors to homeless drug users living in hostels. J Subst Abuse Treat 2014, 47(4):258-264.

13. Neale J, and Stevenson C: The use of computer-assisted therapy by homeless drug users living in hostels: An explorative qualitative study. Drugs: Education, Prevention and Policy 2014, 21(1):80-87.

14. Gelberg L, Browner CH, Lejano E, Arangua L: Access to women's health care: a qualitative study of barriers perceived by homeless women. Women Health 2004, 40(2):87-100.

15. Eapen DJ, Bergh R, Lucas S, Narendorf SC, Begun S, Santa Maria D: Experiences of pregnancy prevention among youth experiencing homelessness. Children and Youth Services Review 2023, 153107115.

16. Adedze M, Osei-Yeboah R, Morhe ESK, Ngambouk VP: Exploring Sexual and Reproductive Health Needs and Associated Barriers of Homeless Young Adults in Urban Ghana: A Qualitative Study. Sex Res Social Policy 2022, 19(3):1006-1019.

17. Kennedy S, Grewal M, Roberts EM, Steinauer J, Dehlendorf C: A qualitative study of pregnancy intention and the use of contraception among homeless women with children. J Health Care Poor Underserved 2014, 25(2):757-770.

18. Kachingwe ON, Anderson K, Houser C, Fleishman JL, Novick JG, Phillips DR, Aparicio EM: “She was there through the whole process:” Exploring how homeless youth access and select birth control. Children and Youth Services Review 2019, 101277-284.

19. Vickery KD, Ford BR, Gelberg L, Bonilla Z, Strother E, Gust S, Adair E, Montori VM, Linzer M, Evans MD, et al: The development and initial feasibility testing of D-HOMES: a behavioral activation-based intervention for diabetes medication adherence and psychological wellness among people experiencing homelessness. Front Psychol 2023, 141225777.

20. Weber JJ, Lee RC, Martsolf D: Experiences of Care in the Emergency Department Among a Sample of Homeless Male Veterans: A Qualitative Study. J Emerg Nurs 2020, 46(1):51-58.

21. McCallum R, Medved MI, Hiebert-Murphy D, Distasio J, Sareen J, Chateau D: Fixed Nodes of Transience: Narratives of Homelessness and Emergency Department Use. Qual Health Res 2020, 30(8):1183-1195.

22. Chinchilla M, Preston-Suni K, Jacobo E, Gabrielian S: Increasing Primary Care Engagement Among Homeless-Experienced Veterans Following an Emergency Department Visit: Qualitative Insights From Los Angeles County. J Prim Care Community Health 2024, 1521501319241296603.

23. Moczygemba LR, Thurman W, Tormey K, Hudzik A, Welton-Arndt L, Kim E: GPS Mobile Health Intervention Among People Experiencing Homelessness: Pre-Post Study. JMIR Mhealth Uhealth 2021, 9(11):e25553.

24. Partida D, Powell J, Ricco M, Naugle J, Magee C, Zevin B, Masson CL, Konadu Fokuo J, Gonzalez D, Khalili M: Formal Hepatitis C Education Increases Willingness to Receive Therapy in an On-site Shelter-Based HCV Model of Care in Persons Experiencing Homelessness. Open Forum Infect Dis 2022, 9(4):ofac103.

25. Masson CL, Fokuo JK, Anderson A, Powell J, Zevin B, Bush D, Khalili M: Clients' perceptions of barriers and facilitators to implementing hepatitis C virus care in homeless shelters. BMC Infect Dis 2020, 20(1):386.

26. Rado N, Bekasi S, Gyorffy Z: Health Technology Access and Peer Support Among Digitally Engaged People Experiencing Homelessness: Qualitative Study. JMIR Hum Factors 2024, 11e55415.

27. Oliveira MA, Boska GA, Oliveira MAF, Barbosa GC: Access to health care for people experiencing homelessness on Avenida Paulista: barriers and perceptions. Rev Esc Enferm USP 2021, 55e03744.

28. Christiani A, Hudson AL, Nyamathi A, Mutere M, Sweat J: Attitudes of homeless and drug-using youth regarding barriers and facilitators in delivery of quality and culturally sensitive health care. J Child Adolesc Psychiatr Nurs 2008, 21(3):154-163.

29. Ensign J, Panke A: Barriers and bridges to care: voices of homeless female adolescent youth in Seattle, Washington, USA. J Adv Nurs 2002, 37(2):166-172.

30. Darbyshire P, Muir-Cochrane E, Fereday J, Jureidini J, Drummond A: Engagement with health and social care services: perceptions of homeless young people with mental health problems. Health Soc Care Community 2006, 14(6):553-562.

31. Henderson MD, McCurry IJ, Deatrick JA, Lipman TH: Experiences of Adult Men Who Are Homeless Accessing Care: A Qualitative Study. J Transcult Nurs 2022, 33(2):199-207.

32. Ensign J, Gittelsohn J: Health and access to care: perspectives of homeless youth in Baltimore City, U.S.A. Soc Sci Med 1998, 47(12):2087-2099.

33. Lewis JH, Andersen RM, Gelberg L: Health care for homeless women. J Gen Intern Med 2003, 18(11):921-928.

34. Ramsay N, Hossain R, Moore M, Milo M, Brown A: Health Care While Homeless: Barriers, Facilitators, and the Lived Experiences of Homeless Individuals Accessing Health Care in a Canadian Regional Municipality. Qual Health Res 2019, 29(13):1839-1849.

35. Hudson AL, Nyamathi A, Greengold B, Slagle A, Koniak-Griffin D, Khalilifard F, Getzoff D: Health-seeking challenges among homeless youth. Nurs Res 2010, 59(3):212-218.

36. Nickasch B, Marnocha SK: Healthcare experiences of the homeless. J Am Acad Nurse Pract 2009, 21(1):39-46.

37. Mnkandla MM, Tshitangano TG, Mudau AG: Healthcare-Seeking Behaviors of Homeless Substance Users During the COVID-19 Lockdowns in Gauteng, South Africa: A COREQ-Based Report. In Social Sciences, vol. 12; 2023.

38. Mc Conalogue D, Maunder N, Areington A, Martin K, Clarke V, Scott S: Homeless people and health: a qualitative enquiry into their practices and perceptions. J Public Health (Oxf) 2021, 43(2):287-294.

39. Prado M, Goncalves M, Silva SSD, Oliveira PS, Santos KDS, Fortuna CM: Homeless people: health aspects and experiences with health services. Rev Bras Enferm 2021, 74(1):e20190200.

40. Wen CK, Hudak PL, Hwang SW: Homeless people's perceptions of welcomeness and unwelcomeness in healthcare encounters. J Gen Intern Med 2007, 22(7):1011-1017.

41. Woith WM, Kerber C, Astroth KS, Jenkins SH: Lessons from the Homeless: Civil and Uncivil Interactions with Nurses, Self-Care Behaviors, and Barriers to Care. Nurs Forum 2017, 52(3):211-220.

42. Haley RJ, Woodward KR: Perceptions of Individuals Who Are Homeless: Healthcare Access and Utilization in San Diego. Advanced Emergency Nursing Journal 2007, 29(4).

43. Eapen DJ, Bergh R, Narendorf SC, Santa Maria DM: Pregnancy and parenting support for youth experiencing homelessness. Public Health Nurs 2022, 39(4):728-735.

44. Kneck A, Mattsson E, Salzmann-Erikson M, Klarare A, in collaboration with Women Advisory Board for Inclusion H: "Stripped of dignity" - Women in homelessness and their perspectives of healthcare services: A qualitative study. Int J Nurs Stud 2021, 120103974.

45. McGonigle K, Carley T, Hoff C: Assessing Racial Disparities in HCV Infection and Care Outcomes in a Southern Urban Population. J Racial Ethn Health Disparities 2018, 5(5):1052-1058.

46. Etchin AG, LaCoursiere-Zucchero T, McDannold SE, McInnes DK: Dual use of Department of Veterans Affairs and community healthcare: Homeless veterans' experiences, perspectives, and perceptions. J Am Assoc Nurse Pract 2021, 33(11):991-998.

47. Bell L, Whelan M, Lycett D, Fernandez E, Khera-Butler T, Kehal I, Patel R: Healthcare and housing provision for a UK homeless community: a qualitative service evaluation. Public Health 2024, 2291-6.

48. Ramirez J, Petruzzi LJ, Mercer T, Gulbas LE, Sebastian KR, Jacobs EA: Understanding the primary health care experiences of individuals who are homeless in non-traditional clinic settings. BMC Prim Care 2022, 23(1):338.

49. Doroshenko A, Hatchette J, Halperin SA, MacDonald NE, Graham JE: Challenges to immunization: the experiences of homeless youth. BMC Public Health 2012, 12338.

50. Jenkinson JIR, Wigle J, Richard L, Tibebu T, Orkin AM, Thulien NS, Kiran T, Gogosis E, Crichlow F, Dyer AP, et al: Structural violence as a driver of COVID-19 vaccine hesitancy and low vaccine uptake among people experiencing homelessness in Toronto, Canada: A qualitative study. Soc Sci Med 2025, 365117588.

51. Grune J, Savelsberg D, Kobus M, Lindner AK, Herrmann WJ, Schuster A: Determinants of COVID-19 vaccine acceptance and access among people experiencing homelessness in Germany: A qualitative interview study. Front Public Health 2023, 111148029.

52. Coe AB, Moczygemba LR, Gatewood SB, Osborn RD, Matzke GR, Goode JV: Medication adherence challenges among patients experiencing homelessness in a behavioral health clinic. Res Social Adm Pharm 2015, 11(3):e110-120.

53. Richler MJ, Yousaf S, Hwang SW, Dewhurst NF: Descriptive study of homeless patients' perceptions that affect medication adherence. Am J Health Syst Pharm 2019, 76(17):1288-1295.

54. Nyamathi A, Shuler P: Factors Affecting Prescribed Medication Compliance of the Urban Homeless Adult. The Nurse Practitioner 1989, 14(8).

55. Paudyal V, MacLure K, Buchanan C, Wilson L, Macleod J, Stewart D: 'When you are homeless, you are not thinking about your medication, but your food, shelter or heat for the night': behavioural determinants of homeless patients' adherence to prescribed medicines. Public Health 2017, 1481-8.

56. Adkins EC, Zalta AK, Boley RA, Glover A, Karnik NS, Schueller SM: Exploring the potential of technology-based mental health services for homeless youth: A qualitative study. Psychol Serv 2017, 14(2):238-245.

57. Guenzel N, Jerreed I, Patrick H, Leeza S, and Hinrichsen S: Mental health, stigma, and barriers to care in a Midwestern sample of homeless individuals. Journal of Social Distress and Homelessness 2020, 29(2):102-109.

58. Adams EA, Parker J, Jablonski T, Kennedy J, Tasker F, Hunter D, Denham K, Smiles C, Muir C, O'Donnell A, et al: A Qualitative Study Exploring Access to Mental Health and Substance Use Support among Individuals Experiencing Homelessness during COVID-19. Int J Environ Res Public Health 2022, 19(6).

59. Mar KL, Mizock L, Veazey C, Nelson A: Mental healthcare barriers and facilitators experienced by homeless women with serious mental illness. Journal of Social Distress and Homelessness 2023, 32(1):59-68.

60. Eshtehardi SS, Taylor AA, Chen TA, de Dios MA, Correa-Fernandez V, Kendzor DE, Businelle MS, Reitzel LR: Sociodemographic Determinants of Nonadherence to Depression and Anxiety Medication among Individuals Experiencing Homelessness. Int J Environ Res Public Health 2021, 18(15).

61. Acorda D, Torres J, Santa Maria D: Acceptability of a Just-in-Time Adaptive Intervention for HIV Prevention among Youth Experiencing Homelessness: A Qualitative Analysis. Journal of Health Care for the Poor and Underserved 2021, 32.

62. Paradis-Gagne E, Jacques MC, Pariseau-Legault P, Ben Ahmed HE, Stroe IR: The perspectives of homeless people using the services of a mobile health clinic in relation to their health needs: a qualitative study on community-based outreach nursing. J Res Nurs 2023, 28(2):154-167.

63. DiMarco MA, Ludington SM, Menke EM: Access to and utilization of oral health care by homeless children/families. J Health Care Poor Underserved 2010, 21(2 Suppl):67-81.

64. Csikar J, Vinall-Collier K, Richemond JM, Talbot J, Serban ST, Douglas GVA: Identifying the barriers and facilitators for homeless people to achieve good oral health. Community Dent Health 2019, 36(2):137-142.

65. Mago A, MacEntee MI, Brondani M, Frankish J: Anxiety and anger of homeless people coping with dental care. Community Dent Oral Epidemiol 2018, 46(3):225-230.

66. Cairns D, Rodriguez A: A stakeholder co-design approach to designing a dental service for adults experiencing homelessness. Front Oral Health 2024, 51355429.

67. Patel EA, Shah SV, Poulson TA, Jagasia AA: An Integrative Model of ENT Healthcare for the Homeless Population. Laryngoscope 2024, 134(6):2705-2709.

68. Hwang SW, Wilkins E, Chambers C, Estrabillo E, Berends J, MacDonald A: Chronic pain among homeless persons: characteristics, treatment, and barriers to management. BMC Fam Pract 2011, 1273.

69. Bloom KC, Bednarzyk MS, Devitt DL, Renault RA, Teaman V, Van Loock DM: Barriers to prenatal care for homeless pregnant women. J Obstet Gynecol Neonatal Nurs 2004, 33(4):428-435.

70. Gordon AC, Lehane D, Burr J, Mitchell C: Influence of past trauma and health interactions on homeless women's views of perinatal care: a qualitative study. Br J Gen Pract 2019, 69(688):e760-e767.

71. Santa Maria D, Gallardo KR, Narendorf S, Petering R, Barman-Adhikari A, Flash C, Hsu HT, Shelton J, Ferguson K, Bender K: Implications for PrEP Uptake in Young Adults Experiencing Homelessness: A Mixed Methods Study. AIDS Educ Prev 2019, 31(1):63-81.

72. Gunner E, Chandan SK, Marwick S, Saunders K, Burwood S, Yahyouche A, Paudyal V: Provision and accessibility of primary healthcare services for people who are homeless: a qualitative study of patient perspectives in the UK. Br J Gen Pract 2019, 69(685):e526-e536.

73. Hamilton AB, Poza I, Hines V, Washington DL: Barriers to Psychosocial Services among Homeless Women Veterans. J Soc Work Pract Addict 2012, 12(1):52-68.

74. Johnston D, McInerney P, Thurling H: Experiences of the homeless accessing an inner-city pharmacy and medical student-run clinic in Johannesburg. Health SA 2020, 251358.

75. Saharan A, Balachander M, Sparke M: Sharing the burden of treatment navigation: social work and the experiences of unhoused women in accessing health services in Santa Cruz. Soc Work Health Care 2021, 60(8-9):581-598.

76. Dave M, Thakrar S, Bagnall H, Kumbang J: Real-time evaluation of a multi-agency TB-screening event for persons experiencing homelessness in a town with a low incidence of TB in England. Epidemiol Infect 2024, 152e73.

77. Hino P, Almeida I, Monroe A, Bertolozzi M, Taminato M, Fornari L, Rosa A: Percepção de pessoas em situação de rua sobre o tratamento da tuberculose. Medicina (Ribeirão Preto) 2022, 55.

78. Garden B, Samarina A, Stavchanskaya I, Alsterlund R, Ovregaard A, Taganova O, Shpakovskaya L, Zjemkov V, Ridell M, Larsson LO: Food incentives improve adherence to tuberculosis drug treatment among homeless patients in Russia. Scand J Caring Sci 2013, 27(1):117-122.

79. Garvin LA, Greenan MA, Edelman EJ, Slightam C, McInnes DK, Zulman DM: Increasing Use of Video Telehealth Among Veterans Experiencing Homelessness with Substance Use Disorder: Design of A Peer-Led Intervention. J Technol Behav Sci 20221-12.
